# Double Porphyrin Cage Compounds

**DOI:** 10.1002/ejoc.202001211

**Published:** 2020-11-16

**Authors:** Kathleen Stout, Theo P. J. Peters, Mathijs F. J. Mabesoone, Fabian L. L. Visschers, Eline M. Meijer, Joëlle‐Rose Klop, Jeroen van den Berg, Paul B. White, Alan E. Rowan, Roeland J. M. Nolte, Johannes A. A. W. Elemans

**Affiliations:** ^1^ Institute for Molecules and Materials Radboud University Heyendaalseweg 135 6525 AJ Nijmegen The Netherlands; ^2^ Australian Institute for Bioengineering and Nanotechnology (AIBN), Corner College and Cooper Rds (Bldg 75) The University of Queensland 4072 Brisbane Qld Australia

**Keywords:** Porphyrin compounds, Host–guest chemistry, Axial ligands

## Abstract

The synthesis and characterization of double porphyrin cage compounds are described. They consist of two porphyrins that are each attached to a diphenylglycoluril‐based clip molecule via four ethyleneoxy spacers, and are linked together by a single alkyl chain using “click”‐chemistry. Following a newly developed multistep synthesis procedure we report three of these double porphyrin cages, linked by spacers of different lengths, i.e. 3, 5, and 11 carbon atoms. The structures of the double porphyrin cages were fully characterized by NMR, which revealed that they consist of mixtures of two diastereoisomers. Their zinc derivatives are capable of forming sandwich‐like complexes with the ditopic ligand 1,4‐diazabicyclo[2,2,2]octane (**dabco**).

## Graphical abstract

The synthesis of double porphyrin cage compounds based on glycoluril is described. Through a multistep synthesis, three double cage compounds linked by spacers of variable length are obtained. The binding and conformational properties of their zinc derivatives towards the ditopic ligand dabco have been investigated into detail.

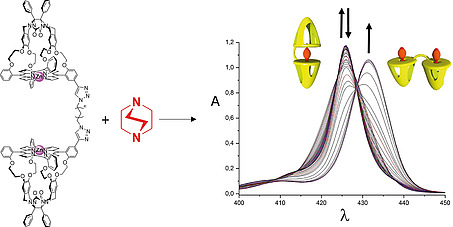

## Introduction

In the past decades, nature has served as a source of inspiration for scientists working on the design of new molecular systems that are capable of mimicking the action of enzymes, e.g. with respect to rate and substrate selectivity. A class of enzymes that stands at the basis of life are the DNA‐polymerases, which, together with exonucleases, replicate and break down the DNA present in organisms.^[^ ^1^ ^]^ During DNA replication, the information stored in the pattern of nucleotides on the encoding (mother) strand is transferred to the new daughter strand. One of the factors contributing to the high replication fidelity of DNA‐polymerases is the fact that they make use of sequential processive catalysis, meaning that the enzyme stays bound to the substrate while it performs multiple rounds of consecutive reactions.^[^ ^2^ ^]^ During these reactions the movement of the polymerase along the biopolymer chain is discrete and unidirectional, as it repeatedly moves one nucleotide further to carry out the next reaction. In this way, information is reliably copied and stored again by making use of the four nucleobases present in the DNA chain.

**Figure 1:**
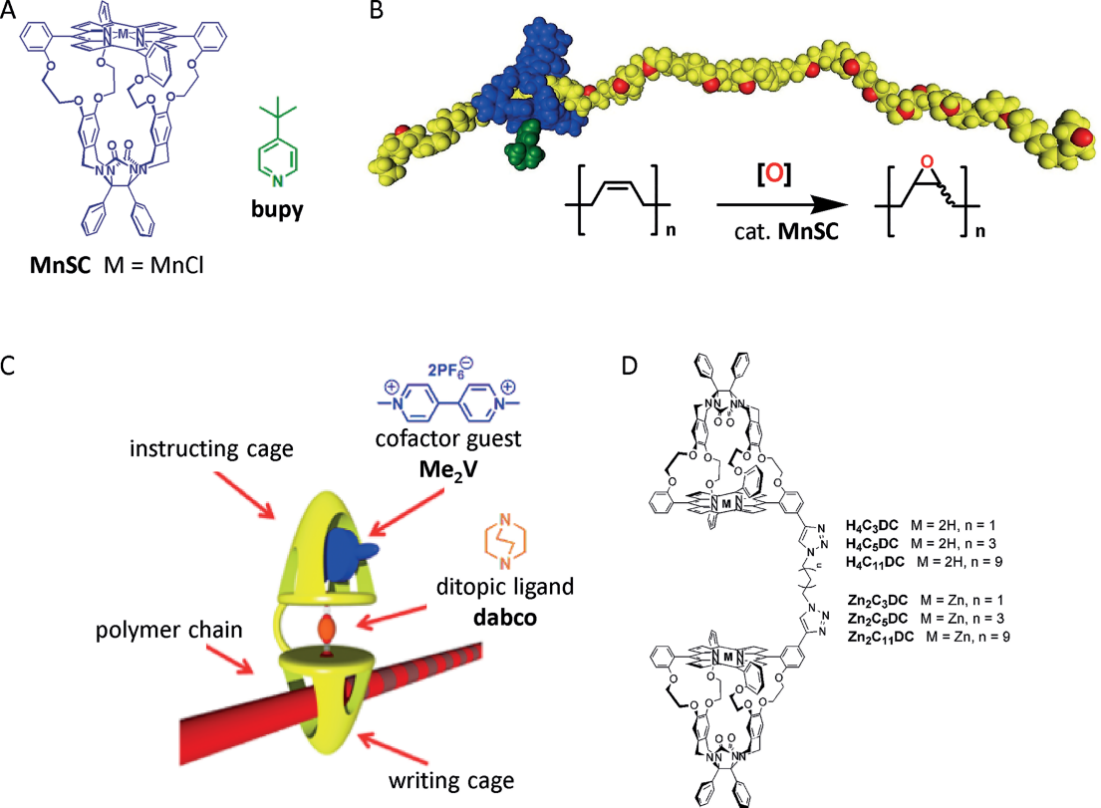
(A) Molecular structure of catalytic porphyrin cage compound **MnSC** and the axial ligand **bupy**. (B) Schematic representation of the processive catalytic conversion of *cis*‐polybutadiene (yellow) into its polyepoxide, carried out by **MnSC** (blue), to which a **bupy** ligand (green) is axially coordinated. (C) Schematic representation of a double porphyrin cage compound (yellow) in which the “writing” cage serves as a processive catalyst for the epoxidation of a polymer chain (red) and the “instructing” cage binds a **Me_2_V** cofactor (blue), which transfers information via the ditopic ligand **dabco** (orange) to the “writing cage”. (D) Molecular structures of the double porphyrin cage compounds described in this paper.

Taking the natural processive enzymes as a blueprint, our group has developed synthetic processive catalysts that are based on a glycoluril‐based manganese porphyrin cage, **MnSC** (Figure 1A).^[^ ^3^ ^]^ This compound is capable of threading an alkene‐containing polymer chain and catalytically converting it to its polyepoxide in an efficient, processive fashion (Figure 1B).^[^ ^4^ ^]^ Our current research focuses on achieving more control over the processive epoxidation catalysis, i.e., in terms of the directionality of performing the catalytic reactions along the polymer chain, and the ability to stereoselectively epoxidize *trans*‐double bonds in the polymers into (*R,R*)‐ or (*S,S*)‐epoxides. When such control can be realized, we envisage the use of the epoxidized polymers as a new type of data storage material,^[^ ^5^ ^]^ with the two mirror image epoxides acting as the digits 0 and 1 in a binary code.

In order to be able to manipulate the reactivity and/or selectivity of the manganese porphyrin cage catalyst, we have designed double porphyrin cage molecules, in which one of the cages is meant to serve as the “writing cage” and the other as the “instructing cage” (Figure 1C‐D). Here, the “writing cage” threads a polymer chain and catalytically converts its double bonds into epoxides. It receives its instructions via an external stimulus, e.g., a light‐induced binding event, coming from the “instructing cage”. This stimulus is transferred via a ditopic ligand, e.g. **dabco** (1,4‐diazabicyclo[2,2,2]octane) that binds between the two cages via their porphyrin metal centers. An example of such an external stimulus could be the binding of a cofactor guest,^[^ ^6^ ^]^ such as **Me_2_V** (*N,N'*‐dimethyl‐4,4‐bipyridinium dihexafluorophosphate). **Me_2_V** is known to have a high affinity for the receptor cavity of the porphyrin cages.^[^ ^7^ ^]^ In this paper we report our synthetic efforts to covalently link two porphyrin cage compounds in a geometry that ensures close proximity of the metal centers in the two porphyrin planes (Figure 1D). We previously showed that the coordination of axial ligands such as **dabco** to the metal center in the porphyrin cage is allosterically influenced by the binding of a **Me_2_V** guest inside the cavity.^[^ ^8^ ^]^ We here describe the synthesis of 3 double porphyrin cages, which differ in the length of the linker that connects the two separate porphyrin rings. Furthermore, we report the ditopic coordination of **dabco** to the zinc derivatives of the double cage compounds, yielding stable 1:1 sandwich complexes.

## Results and Discussion

**Scheme 1:**
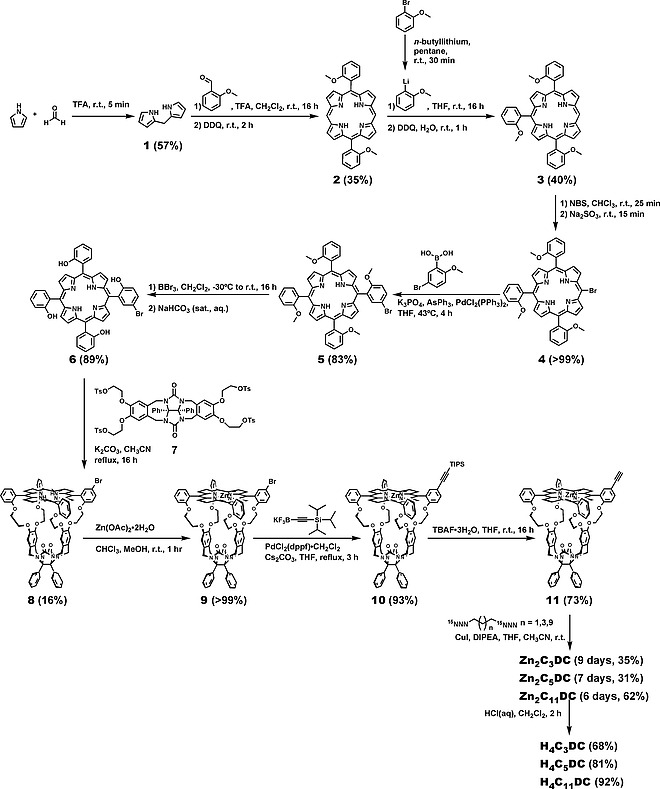
Synthesis of double porphyrin cage compounds.

**Synthesis**. Conventionally, porphyrin cage compounds are prepared via a fourfold nucleophilic substitution reaction of tetratosyl‐functionalized molecular clip **7** (Scheme 1) with tetrakis‐*meso*‐*ortho*‐hydroxyphenyl porphyrin.^[^ ^9^ ^]^ For the synthesis of the double porphyrin cages, the latter compound needed to be equipped on one of the *meso*‐aryl substituents with a functional group that would allow further conversion to a linker between the two cages. To synthesize such a mono‐functionalized porphyrin, we designed the synthetic route that is depicted in Scheme 1. It starts with an acid‐catalyzed condensation of paraformaldehyde with pyrrole to provide dipyrromethane **1** in 57 % yield.^[^ ^10^ ^]^ A MacDonald [2+2] condensation of this compound with 2‐methoxybenzaldehyde and subsequent oxidation of the porphyrinogen gave 5,15‐bis(2‐methoxyphenyl)porphyrin **2** in 35 % yield.^[^ ^11^ ^]^ The attachment of the third *meso*‐aryl substituent was accomplished by nucleophilic addition of *o*‐methoxyphenyllithium (obtained by a reaction of 2‐bromoanisole with *n*‐butyllithium) to one of the available *meso*‐positions of **2**.^[^ ^11^, ^12^ ^]^ After oxidation with DDQ, compound **3** was obtained in 40 % yield. The remaining free *meso*‐position of this compound is unsuitable for a similar reaction with an appropriate lithium compound to prepare the desired mono‐functionalized porphyrin, because a reaction of the lithium salt with the free *meso*‐position yields a Meisenheimer‐type complex. The negative charge of this complex is localized and stabilized at the opposite *meso*‐position of the porphyrin, which therefore should be unsubstituted.^[^ ^13^ ^]^ As an alternative, we selected a Suzuki cross‐coupling reaction to introduce the final *meso*‐aryl ring. To this end, the free *meso*‐position of **3** was first regioselectively brominated with NBS, giving compound **4** in quantitative yield.^[^ ^14^ ^]^ A subsequent cross‐coupling of **4** with 5‐bromo‐2‐methoxyphenyl boronic acid, in the presence of a palladium catalyst, gave porphyrin **5** in 83 % yield.^[^ ^15^ ^]^ To prevent additional cross‐coupling reactions at the bromo‐functionalized phenyl ring, product formation of **5** was monitored with the help of mass spectrometry (MS), and the reaction was carried out at a moderate temperature. At the moment that MS showed full conversion of **4**, the reaction was stopped. As a final step, the methoxy groups of **5** were deprotected using boron tribromide to give the desired mono‐bromo‐functionalized tetrahydroxy porphyrin **6** in 89 % yield. Compound **6** was then coupled to clip **7** ^[^ ^9^ ^]^ to give the mono‐bromo‐substituted porphyrin cage compound **8** in 16 % yield. To protect the porphyrin of **8** from undesired metal insertion in following synthesis steps, it was metallated by inserting a zinc(II) center, providing compound **9** in quantitative yield. To convert **9** into a suitable precursor for a copper‐catalyzed azide‐alkyne 1,3‐dipolar cycloaddition (CuAAC) reaction, the bromide substituent was converted into an alkyne via a Suzuki–Miyaura cross coupling reaction. Using potassium triisopropylsilylacetylene trifluoroborate as the alkyne source,^[^ ^16^ ^]^ compound **10** was obtained in 93 % yield. Subsequent deprotection of the alkyne with tetrakis‐*n*‐butylammonium fluoride provided mono‐acetylene‐functionalized porphyrin cage **11** in 73 % yield. Using CuAAC, this compound was subsequently reacted with α,ω‐diazidoalkane linkers of different length to obtain a series of three double cage compounds with various spacers lengths between the two cages. To facilitate the characterization of the new compounds by NMR, ^15^N‐enriched α,ω‐diazidoalkanes were synthesized according to a literature procedure using ^15^N‐enriched sodium azide, in which the ^15^N isotope is located at one of the two terminal azide positions.^[^ ^17^ ^]^ CuAAC reactions of **11** with the respective diazides provided zinc double porphyrin cages **Zn_2_C_3_DC**, **Zn_2_C_5_DC** and **Zn_2_C_11_DC** in yields of 35, 31 and 62 %, respectively. The rather low yields of the double click reactions are attributed to steric hindrance between the two approaching porphyrin cages. During purification by column chromatography, it was possible to retrieve unreacted **11**, which could be re‐employed in subsequent click reactions. After treatment of the **Zn_2_C_x_DC** (**x** = 3, 5 or 11) compounds with aqueous hydrochloric acid, free base double porphyrin cage compounds **H_4_C_3_DC**, **H_4_C_5_DC** and **H_4_C_11_DC** were obtained in yields of 68, 81, and 92 %, respectively.

**Figure 2:**
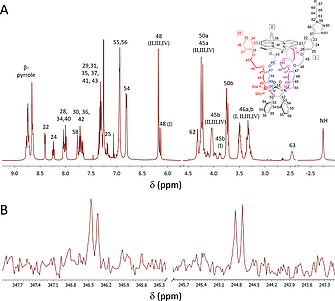
(A) ^1^H NMR spectrum of **H_4_C_3_DC** in CDCl_3_ and proton/carbon numbering of the compound. The Roman numbers I, II, III and IV and the related colors indicate the four symmetry quandrants within the porphyrin cage structure. (B) ^15^N spectrum of **H_4_C_3_DC** in CDCl_3_.

**Structural characterization**. The double porphyrin cage compounds were characterized with the help of ^1^H, ^13^C and ^15^N NMR spectroscopy in chloroform solution. All proton and carbon resonances could be readily assigned with the help of 2D techniques (see Figure 2 for the assignment of **H_4_C_3_DC**). The NMR measurements revealed the presence of several structural isomers of the double porphyrin cage compounds. The ^15^N spectrum of **H_4_C_3_DC** showed the presence of four singlets for the ^15^N‐labeled nitrogen atoms present in the triazole rings of the spacer between the two porphyrin cages (Figure 2B). The fact that two sets of two singlets are observed for the two different types of ^15^N‐labeled nitrogen atoms indicates that two different species of **H_4_C_3_DC** must be present. A ^1^H‐^15^N HBMC spectrum revealed couplings of both the nitrogen signals ^15^N‐61 at 346.54 and 346.45 ppm with triazole proton signal H‐58 at 7.77 ppm. Also the signals of ^15^N‐59 at 244.89 and 244.77 ppm coupled with this triazole signal, as well as to proton signals at 4.32 and 2.47 ppm belonging to CH_2_‐62 and CH_2_‐63, respectively. Via a ROE contact between H‐58 and the phenyl proton H‐22 in the 2D ROESY spectrum, the resonance of the latter proton in the ^1^H NMR spectrum could be assigned (8.40 ppm). C‐22 displays a ^13^C‐HMBC coupling to H‐24 (8.28–8.21 ppm), which in turn displays *two* COSY cross coupling peaks with H‐25. The fact that for this proton two well‐separated resonances are observed confirms the abovementioned existence of two different species. The two signals integrate equally, meaning that the two species are present in a 1:1 ratio. In addition to H‐25, also H‐45, H‐46, and H‐48 in the same quadrant of the molecule (denoted “I” in Figure 2) give two distinct signals in the NMR spectrum, all confirming the existence of two different species. Conversely, the analogous proton signals in the other three quadrants of the molecule (II, III and IV) do not show such a splitting of signals.

**Figure 3:**
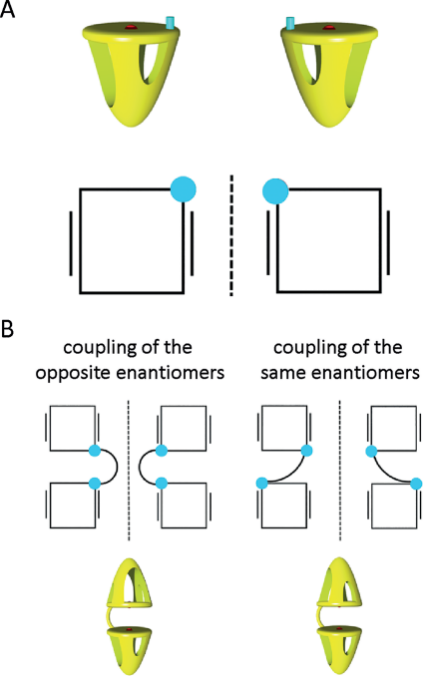
(A) Representations of the two enantiomers of monofunctionalized single cage compounds **8–11**. Top: 3D representations. Bottom: schematic view of the porphyrin (black square) seen from the top. The black lines left and right from the square indicate the location of the o‐xylylene side walls of the cage, the cyan dot the functional group at the top. (B) Representations (top views and 3D views) of the two diastereoisomers that are obtained by the coupling of the enantiomers of monofunctionalized single cage compound **11**.

The observation that **H_4_C_3_DC** exists as two equally abundant species is the result of the fact that this compound, as well as the other double porphyrin cage compounds, are mixtures of two diastereoisomers. The monofunctionalized single porphyrin cage compounds **8–11** are all racemates of two enantiomers. Although their chiral nature is at first sight not easily recognizable, their macrocyclic three‐dimensional (3D) structure gives rise to the existence of two non‐superimposable mirror‐image structures, i.e., enantiomers (Figure 3A). The compounds exhibit planar chirality,^[^ ^18^ ^]^ and the 3D chiral morphology leads in this specific case to the emergence of two chiral centers, i.e., the two quaternary bridgehead carbon atoms in the diphenylglycoluril framework (C‐52, see Figure 2A).^[^ ^19^ ^]^ The subsequent connection of two molecules of (*rac*)‐**11** via click chemistry leads to the expected formation of two diastereoisomers in equal quantities (Figure 3B). So far, we have not succeeded in separating the diastereoisomers via chromatographic methods. Besides **H_4_C_3_DC**, also **H_4_C_5_DC** displayed two sets of signals for protons H‐25, H‐45, H‐46 and H‐48 in its ^1^H NMR spectrum. In contrast, **H_4_C_11_DC** did not display double resonance sets for these protons. This is presumably the result of the spacer length in this double cage compound. The longer spacer increases the distance between the two cages so much that they no longer experience chemical environments that are different enough to cause double resonances for the same protons in the NMR spectrum.

Insertion of a zinc center in the porphyrin led to broadening of the proton signals of the double zinc porphyrin cage compounds **Zn_2_C_x_DC** in their ^1^H NMR spectra in CDCl_3_. The addition of deuterated pyridine to these solutions caused a sharpening of the signals, which suggests that the signal broadening is caused by coordination interactions (intramolecular or intermolecular) between the triazole nitrogen or carbonyl oxygen atoms and the porphyrin zinc centers, which are broken by coordination of the deuterated pyridine to the zinc centers. To characterize the **Zn_2_C_x_DC** compounds, we recorded their NMR spectra at 60 °C in [D_6_]DMSO, a solvent that also prohibits such intramolecular or intermolecular coordination. The spectra indeed displayed sharp resonances, which could be completely assigned to the two diastereoisomers that were also observed for the **H_4_C_x_DC** compounds.

**Figure 4:**
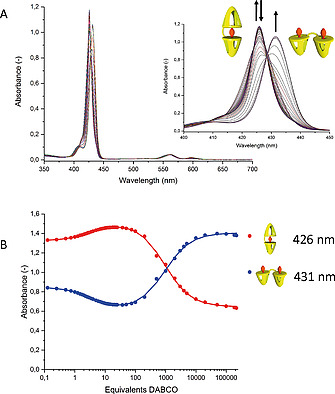
(A) Changes in UV/Vis spectra of **Zn_2_C_3_DC** upon the addition of **dabco**, in CHCl_3_/CH_3_CN, 1:1 (v/v). (B) Titration curves extracted from the spectral changes in (A), monitoring the absorbance of the bands at 426 (red) and 431 nm (blue). The solid lines through the data points represent fits assuming a standard 1:2 binding model.

**Coordination complexes of the double zinc porphyrin cage compounds with dabco**. The ability of the double zinc porphyrin cages to coordinate the ditopic ligand **dabco** was investigated. The coordination of this ligand to zinc porphyrins and the typical spectral changes it induces in NMR and optical spectra have been reported extensively.^[^ ^20^ ^]^ To enable future follow‐up studies with chloroform‐insoluble viologen guests, the ligand coordination studies were carried out in a solvent mixture of chloroform and acetonitrile (1:1, v/v). While in chloroform the **Zn_2_C_x_DC** compounds all displayed a Soret band at 421 nm, in CHCl_3_/CH_3_CN this band shifts to 426 nm, which we attribute to axial coordination of acetonitrile to the zinc centers. When **dabco** is added to a solution of **Zn_2_C_3_DC**, the band at 426 nm initially does not shift, but sharpens and increases in intensity (Figure 4A), which indicates the formation of a more well‐defined species. After the addition of several equivalents of **dabco**, the spectral changes remain virtually unchanged up to the addition of several hundreds of equivalents of the ligand (Figure 4B). In the presence of a larger excess of **dabco**, the band at 426 nm again decreases in intensity, with a concomitant emergence of a new band at 431 nm, which increases in intensity as up to 5 × 10^5^ equivalents of **dabco** are added.

The initial sharpening of the Soret band is attributed to the formation of a 1:1 sandwich complex between **Zn_2_C_3_DC** and **dabco**.^[^ ^21^ ^]^ Such a complex is better defined and more rigid than the open‐folded complex between **Zn_2_C_3_DC** and axially coordinated acetonitrile molecules. Moreover, due to effective molarity and binding statistics effects, this complex is highly stable and only upon the addition of a large excess of the ligand, the emergence of a red‐shifted Soret band at 431 nm reflects the formation of a 1:2 **Zn_2_C_3_DC**:**dabco** complex, which is again open‐folded.^[^ ^20^ ^]^ UV/Vis titrations of **Zn_2_C_5_DC** and **Zn_2_C_11_DC** with **dabco** gave similar results (see Supporting Information).

**Figure 5:**
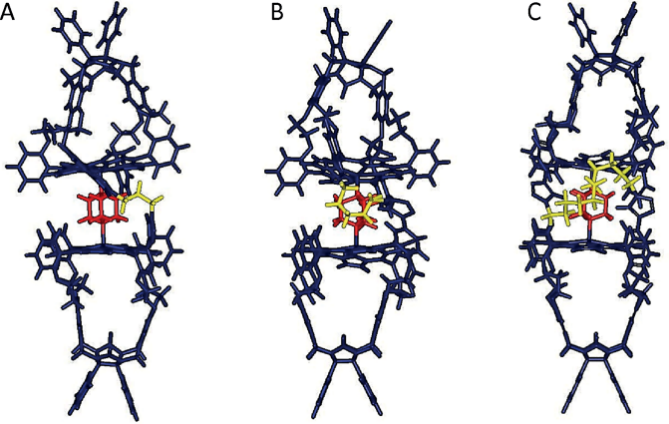
Computer‐modeled structures of the sandwich complexes of double zinc porphyrin cages (indigo) (A) **Zn_2_C_3_DC**, (B) **Zn_2_C_5_DC** and (C) **Zn_2_C_11_DC** with **dabco** (red). The alkyl spacers between the cages are indicated in yellow.

The obtained titration curves were fitted with a standard 1:2 binding model (Figure 4B). The association constants *K*_a_ and binding free energies Δ*G* ^θ^ were extracted from the fits and are summarized in Table 1.^[^ ^22^ ^]^ The *K*_a_‐values obtained for all the double zinc porphyrin cage compounds are comparable to those obtained previously for the coordination of **dabco** to other bis‐zinc porphyrin compounds, which range between 10^4^ ‐ 10^8^ m ^–^^1^ for *K*_a_ (1:1) and 10^2^ – 10^3^ m ^–^^1^ for *K*_a_ (1:2) in various solvents.^[^ ^20^ ^]^ Due to possible competition for axial ligation to the zinc centers between **dabco** and the acetonitrile solvent, the association constants determined here are on the lower end of the commonly reported range.^[^ ^23^ ^]^ While the *K*_a_(1:1)‐values of the complexes of **dabco** with **Zn_2_C_5_DC** and **Zn_2_C_11_DC** are quite similar, the *K*_a_(1:1)‐value of the complex with **Zn_2_C_3_DC** is significantly higher. We attribute this difference to a more favorable effective molarity effect for the second zinc porphyrin in the latter complex: once the first zinc porphyrin of **Zn_2_C_3_DC** has coordinated a **dabco** ligand, the second zinc porphyrin is in close proximity as a result of the relatively short spacer between the two porphyrins. This effect is favorable for 1:1 complexation and the generation of a high *K*_a_(1:1)‐value, but since the difference in *K*_a_(1:1)‐value between the complexes of **dabco** with **Zn_2_C_5_DC** and **Zn_2_C_11_DC** is much less prominent, probably also other factors play a role. Molecular modelling calculations of the complexes (Spartan™, PM3/semi‐empirical) revealed that the short C_3_‐spacer allows an almost perfect fit for the **dabco** ligand between the zinc porphyrins of **Zn_2_C_3_DC**. The longer C_5_ and C_11_ spacers of the other two double zinc porphyrin cages require progressive folding of their chain to still allow a ditopic binding of the ligand (Figure 5), which may be a factor that leads to a lowering of the binding strength of the ligand.

**Table 1:** Association constants *K*_a_ [M – 1] and binding free energies Δ*G* ^θ^ (kJ mol^–1^) for the 1:1 and 1:2 complexes between **Zn_2_C_x_DC** and **dabco** in CHCl_3_/CH_3_CN, 1:1 (v/v). The error represents the standard deviation (±1 S.D.) over a triplo of measurements

			1:1 Complex			2:1 Complex	
		*K*_a_		Δ *G* ^ θ ^	*K*_a_		Δ *G*
**Zn_2_C_3_DC**	1.95 ± 0.3 × 10^6^			–35.9 ± 0.8	130 ± 30		–12.1 ± 0.5
**Zn_2_C_5_DC**	2.34 ± 0.3 × 10^5^			–30.6 ± 0.2	740 ± 90		–16.4 ± 0.3
**Zn_2_C_11_DC**	3.31 ± 0.3 × 10^5^			–31.5 ± 0.2	350 ± 25		–14.5 ± 0.2

**Figure 6:**
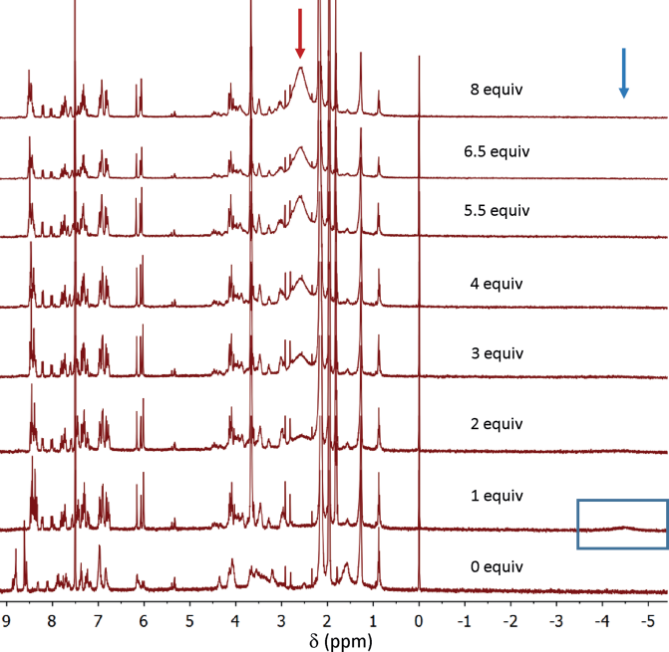
^1^H NMR titration of **Zn_2_C_3_DC** with 0 to 8 equiv. of **dabco** (500 MHz, 298 K, CDCl_3_/CD_3_CN, 1:1 (v/v)). The blue box and arrow indicate the location of the signal of **dabco** in the 1:1 sandwich complex, and the red arrow the signal of non‐coordinated **dabco**.

To corroborate the formation of the 1:1 and 1:2 complexes identified by the UV/Vis titration experiments, NMR studies were carried out for the complex formation between **dabco** and **Zn_2_C_3_DC**, at various temperatures in CDCl_3_/CD_3_CN, 1:1 (v/v). Figure 6 shows the changes in the ^1^H NMR spectra at 298 K when up to 8 equivalents of **dabco** were added to a 1 mm solution of **Zn_2_C_3_DC**. The addition of one equivalent of the ligand caused an upfield shift of the porphyrin β‐pyrrole signals from 8.9–8.5 to 8.5–8.3 ppm, while a new, very broad signal appeared at –4.5 ppm. The latter signal is characteristic for a **dabco** ligand bound in a sandwich‐like geometry between two zinc porphyrins.^[^ ^20^ ^]^ Its broadness is probably caused by rapid exchange between the bound and unbound components on the NMR timescale. In the presence of two or more equivalents of **dabco**, a broad signal corresponding to uncomplexed ligand (at 2.6 ppm) appeared, while the signal at –4.5 ppm disappeared. Remarkably, no signal corresponding to a **dabco** ligand in an open‐folded 1:2 complex was observed. In such a complex, the signal of the methylene‐protons of bound **dabco** are expected around –3 ppm. This absence may again be due to signal broadening as a result of rapid exchange.^[^ ^20^, ^24^ ^]^

**Figure 7:**
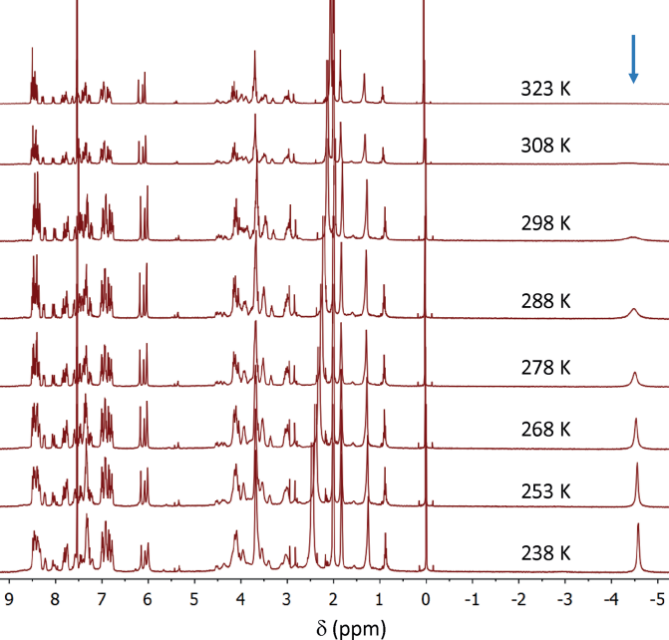
Variable temperature ^1^H NMR spectra of the 1:1 sandwich complex between **Zn_2_C_3_DC** and **dabco** (400 MHz, CDCl_3_/CD_3_CN, 1:1 (v/v)). The arrow indicates the location of the signal of **dabco** in the 1:1 sandwich complex.

To decrease the exchange dynamics, NMR studies were carried out at lower temperature (Figure 7). At temperatures lower than 278 K the broad resonance at –4.5 ppm sharpened into a well‐defined peak at –4.46 ppm, which shifted slightly upfield to –4.6 ppm at 238 K, while simultaneously the signals of **Zn_2_C_3_DC** gradually broadened.

**Figure 8:**
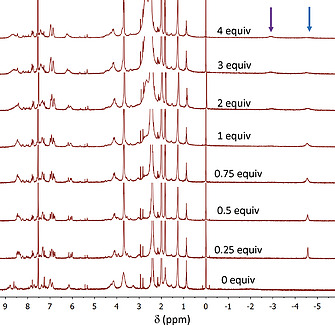
^1^H NMR titration of **Zn_2_C_3_DC** with 0 to 4 equiv. of **dabco** (500 MHz, 248 K, CDCl_3_/CD_3_CN, 1:1 (v/v)). The blue and purple arrows indicate the locations of the signals of **dabco** in the 1:1 sandwich complex (–4.58 ppm) and in the 2:1 open‐folded complex (–2.92 ppm), respectively.

To investigate the binding of **dabco** into further detail, up to 4 equivalents of the ligand were added to the solution of **Zn_2_C_3_DC** and NMR spectra were recorded at 248 K (Figure 8). In the presence of 0.25 equivalents of **dabco**, the signals of **Zn_2_C_3_DC** sharpened, indicating a decrease in dynamics of the cage molecule. When the amount of **dabco** was increased to 1 equivalent, the resonance of the sandwiched ligand at –4.58 ppm slightly broadened. The addition of more equivalents of **dabco** led to the emergence of a signal of the mono‐coordinated ligand in the open‐folded 1:2 complex at –2.92 ppm (α‐protons of the ligand), while simultaneously the resonance at –4.58 ppm decreased in intensity. With the use of 2D NMR techniques we were unable to locate the signals of the β‐protons of the mono‐coordinated **dabco** ligand or the signal of the free ligand, which is probably due to the broadness of the signals and the poor signal‐to‐noise ratio under these conditions. Due to the gradual broadening of the **dabco** resonances, no reliable baseline correction could be applied and, therefore, no reliable ratio of the 1:1 and 1:2 complexes could be determined by integration of the signals. The signals of **Zn_2_C_3_DC** broadened and became less defined upon the addition of more equivalents of **dabco**, which indicates an increase in the dynamics of the double cage compound. Although the NMR signals become better resolved at temperatures lower than 288 K, it can be expected that the complexes also exist at 298 K, albeit more dynamic and in abundancies that are governed by the association constants at that temperature. Thus, the NMR results corroborate the binding geometries as proposed by the UV/Vis experiments.

## Conclusions

We have presented a multistep route for the synthesis of covalently linked double porphyrin cage compounds. For the synthesis of these compounds, a mono‐bromo‐substituted porphyrin was synthesized in 6 steps starting from paraformaldehyde and pyrrole. Upon connection of the porphyrin to a cavity molecule based on diphenylglycoluril, a racemate of two planar‐chiral porphyrin cage molecules was obtained. After converting the bromo‐substituent of these cage compounds into an acetylene function, double porphyrin cage compounds could be prepared via “click” reactions with α,ω‐diazidoalkanes of different lengths. With the help of extensive NMR spectroscopy experiments the double cage compounds could be fully characterized. The presence of multiple resonances for several protons of the double cages with C_3_ and C_5_ spacers proved their existence as two diastereoisomers, while the absence of such multiple resonances in the case of the double cage compound with the C_11_ spacer indicated that the two cavities of that compound are so remote that they are no longer affected by each other's chirality. The ability of the zinc double porphyrin cage compounds to form sandwich complexes with the ditopic ligand **dabco** was confirmed by UV/Vis and NMR titration studies. These revealed that the zinc double cage with the short C_3_ spacer formed the strongest 1:1 sandwich complex with the ligand, probably as a result of a favourable effective molarity effect and binding geometry. As expected, the 1:2 open complexes displayed association constants of about 3 orders of magnitude lower than those of the respective 1:1 complexes. The formation of the 1:1 and 1:2 complexes were corroborated by variable temperature ^1^H NMR titrations. Future research will focus on the possibilities to transfer information between the receptor cavities of the sandwich complexes during catalytic reactions. We will equip the double porphyrin cage compounds with one zinc and one catalytic manganese center, and investigate the influence of the binding of guests in the zinc porphyrin cage, via the coordinated **dabco** ligand, on the processive epoxidation of threaded polyalkenes by the manganese cage of the system.

## Experimental Section

**Figure 9:**
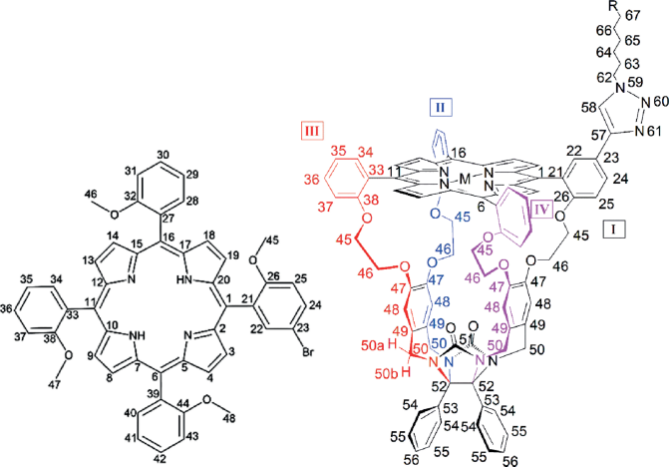
Carbon, proton, and nitrogen numbering of 5‐(5‐bromo‐2‐methoxyphenyl)‐10,15,20‐tris(2‐methoxyphenyl) porphyrin **5** (left) and the double cage compounds **M_2_C_n_DC** (**M** = 2H or Zn, ***n*** = 3, 5 or 11) (right) used for all NMR analyses. For the double cage compounds, R represents the mirror image of the cage molecule, with the restriction that for **Zn_2_C_3_DC** the attachment starts at carbon number 63, for **Zn_2_C_5_DC** at carbon atom 64, and for **Zn_2_C_11_DC** at carbon atom 67 (the latter is shown). The CH_2_‐groups of the diphenylglycoluril framework (carbon atoms 45, 46, 48 and 50) all have two inequivalent geminal protons, marked as a and b. The location of the signals of these protons could only be identified for CH_2_‐50, with the help of ROESY experiments.

**Materials and methods**. All commercially obtained chemicals were used without further purification, unless stated otherwise. Dry *n*‐pentane was stored under argon in a glovebox, THF was distilled under nitrogen from potassium, CH_2_Cl_2_ was distilled under nitrogen from calcium hydride, and MeCN was distilled under argon from calcium chloride. For TLC analysis, TLC Silicagel 60 F_254_ (Merck) and for column chromatography, Silica gel 0.035–0.070 mm 60A (Acros), SilicaFlash® P60 40–63 µm (SiliCycle) or Silicagel 60 H (Merck) were used. ^1^H, ^13^C NMR and ^15^N NMR spectra were recorded on Bruker Avance III 400 or 500 MHz spectrometers at 25 °C unless stated otherwise. Chemical shifts are reported in parts per million (ppm) relative to tetramethylsilane (TMS, 0.00 ppm) as the internal reference. NMR data are presented as follows: chemical shift (∂) in ppm, multiplicity (s = singlet, bs = broad singlet, d = doublet, t = triplet, q=quartet, td = triplet of doublets, m = multiplet and/or multiple resonances), integration, assignment and coupling constant (*J*) in Hertz (Hz). All NMR signals were assigned on the basis of ^1^H, ^13^C NMR, ^15^N NMR, COSY, ROESY, HSQC, and HMBC experiments. The numbering of the proton, carbon, and nitrogen atoms used in the assignments is depicted in Figure 9. Phase and baseline correction was applied to all NMR spectra. As several porphyrin compounds were obtained as a mixture of atropisomers that were inseparable by column chromatography, the reported chemical shifts represent the averaged shifts over all of these atropisomers. Assigning all inequivalent ^13^C‐signals was not possible due to the limited resolution of the 2D spectra and overlapping proton signals, therefore ranges are reported for several almost identical ^13^C atoms. LCQ mass spectra were recorded in methanol on a Thermo Finnigan LCQ Advantage Max mass spectrometer, and MALDI‐TOF mass spectra were measured in reflective mode with dithranol as matrix on a Bruker Microflex LRF MALDI‐TOF mass spectrometer. Accurate MALDI‐TOF mass spectra were obtained on a Brukerdaltonics autoflex (ST‐A2130) MALDI‐TOF mass spectrometer with cesium triiodide as matrix in reflective mode. Accurate masses were obtained from a solution of the compound in methanol on a JEOL AccuTOF CS JMS‐T100CS. Compound **7** was synthesized according to a literature procedure.^[^ ^9^ ^]^

**Syntheses**. For the NMR assignments of proton, carbon and nitrogen signals, the atom numbering depicted in Figure 9 will be used.

**Di(1*H*‐pyrrol‐2‐yl)methane (1)**. This compound was synthesized according to modified literature procedures.^[^ ^10^ ^]^ To distilled pyrrole (153 mL, 2.21 mol) a 37 % aq. formaldehyde solution (9 mL, 90.42 mmol) was added. Argon was led through the solution for 15 min, after which TFA was added (0.82 mL, 10.21 mmol). The reaction mixture was stirred at r.t. for 5 min. The orange solution was diluted with CH_2_Cl_2_ (150 mL) and washed with sat. aq. Na_2_CO_3_ (3 × 100 mL) and water (100 mL). The organic layer was concentrated in vacuo. The excess of pyrrole was removed by vacuum distillation at 35 °C. Silicagel column chromatography using DCM/*n*‐heptane (60:40 (v/v)) as the eluent afforded **1** (7.469 g, 51 mmol, 57 %) as a white fluffy solid.

^1^H‐NMR (CDCl_3_, 400 MHz): ∂ 7.91 (bs, 2H, N*H*), 6.68 (td, 2H, Ar*H*‐5, *J* = 2.6, 1.5 Hz), 6.15 (q, 2H, Ar*H*‐4, *J* = 2.9 Hz), 6.05–6.02 (m, 2H, Ar*H*‐3), 3.99 (s, 2H, C*H*_2_‐1); ^13^C{^1^H}‐NMR (CDCl_3_, 100 MHz): ∂ 129.03 (Ar*C*‐2), 117.24 (Ar*C*‐5), 108.38 (Ar*C*‐4), 106.36 (Ar*C*‐3), 26.38 (*C*H_2_‐1).

**5,15‐Bis(2‐methoxyphenyl)porphyrin (2)**. Argon was led through CH_2_Cl_2_ (1.85 L) for 30 min and compound **1** (1.205 g, 8.24 mmol), 2‐methoxybenzaldehyde (1.122 g, 8.24 mmol) and TFA (0.140 mL, 1.82 mmol) were added. The reaction mixture was stirred in the dark at r.t. for 16 h. Then a solution of DDQ (2.685 g, 11.83 mmol) in CH_2_Cl_2_ (70 mL) was added. After stirring for 2 h the mixture was quenched with Et_3_N (6 mL) and concentrated in vacuo. The residue was purified over a silica gel column using chloroform as the eluent. The crude product was dissolved in CH_2_Cl_2_, filtered, and precipitated in *n*‐heptane. After washing the precipitate with *n*‐pentane (4×), drying in vacuo yielded **2** (749.1 mg, 1.43 mmol, 35 %) as a purple solid containing 2 atropisomers.

^1^H‐NMR (CDCl_3_, 500 MHz): ∂ 10.23 (s, 2H, Ar*H*‐1,11), 9.33 (d, 4H, β‐pyrrole‐*H*‐3,9,13,19, *J* = 4.5 Hz), 8.97 (d, 4H, β‐pyrrole‐*H*‐4,8,14,18, *J* = 4.5 Hz), 8.06 (d, 2H, Ar*H*‐28,40, *J* = 7.2 Hz), 7.80 (t, 2H, Ar*H*‐30,42, *J* = 7.9 Hz), 7.41 (t, 2H, Ar*H*‐29,41, *J* = 7.5 Hz), 7.38 (d, 2H, Ar*H*‐31,43, *J* = 8.0 Hz), 3.61 (s, 6H, O*Me*‐46,48), –3.07 (s, 2H, N*H*); ^13^C{^1^H}‐NMR (CDCl_3_, 126 MHz): ∂ 159.45 (Ar*C*‐32,44), 147.28 (Ar*C*‐5,7,15,17), 145.18 (Ar*C*‐2,10,12,20), 135.80 (Ar*C*‐28,40), 131.33 (Ar*C*‐3,9,13,19), 130.68 (Ar*C*‐4,8,14,18), 130.29 (Ar*C*‐27,39), 129.89 (Ar*C*‐30,42), 119.61 (Ar*C*‐29,41), 115.01 (Ar*C*‐6,16), 111.07 (Ar*C*‐31,43), 104.78 (Ar*C*‐1,11), 55.81 (O*Me*‐46,48); UV/Vis (CHCl_3_): λ/nm (log (ε/M^–1^ cm^–1^)) 408 (5.53), 502 (4.21); Accurate mass: *m/z* 523.21361 [M + H]^+^; calcd. for C_34_H_27_N_4_O_2_ 523.213040; Anal. Calcd for C_34_H_26_N_4_O_2_: C, 78.14; H, 5.01; N, 10.72; found C, 77.99; H, 4.92; N, 10.68.

**5,10,15‐Tris(2‐methoxyphenyl)porphyrin (3)**. 2‐Bromoanisole (2.763 g, 14.77 mmol) was added to dry *n*‐pentane (50 mL). *n*‐Butyllithium (1.6 m in hexanes, 8 mL, 12.79 mmol) was added and the reaction mixture was stirred for 30 min at r.t. until a white precipitate was formed. The mixture was filtered under Schlenk conditions and the white residue was washed with dry *n*‐pentane (4 × 40 mL), after which it was dissolved in distilled THF (150 mL). Compound **2** (390 mg, 0.75 mmol) was added and the reaction mixture was stirred at r.t. for 16 h, after which water (3.5 mL) was added, followed by DDQ (761.32 mg, 3.35 mmol). After stirring for 1 h at r.t. the reaction mixture was evaporated to dryness and the residue was purified over a silica gel column using chloroform as the eluent. The crude product was subsequently purified by silica gel column chromatography using DCM/*n*‐heptane (75:25, (v/v)) as the eluent to yield **3** (187.7 mg, 0.29 mmol, 40 %) as a purple solid containing 3 atropisomers.

^1^H‐NMR (CDCl_3_, 500 MHz): ∂ 10.11 (s, 1H, Ar*H*‐1), 9.25 (d, 2H, β‐pyrrole‐*H*‐3,19, *J* = 4.6 Hz), 8.90 (d, 2H, β‐pyrrole‐*H*‐4,18, *J* = 4.4 Hz), 8.79–8.75 (m, 4H, β‐pyrrole‐*H*‐8,9,13,14), 8.04 (d, 1H, Ar*H*‐34, *J* = 7.3 Hz), 8.02–7.92 (m, 2H, Ar*H*‐28,40), 7.80–7.70 (m, 3H, Ar*H*‐30,36,42), 7.39–7.35 (m, 3H, Ar*H*‐29,35,41), 7.35–7.26 (m, 3H, Ar*H*‐31,37,43), 3.61–3.51 (m, 9H, O*Me*‐46,47,48), –2.89 (s, 2H, N*H*); ^13^C{^1^H}‐NMR (CDCl_3_, 126 MHz): ∂ 159.56–159.37 (Ar*C*‐32,38,44), 149.00–144.00 (broad, Ar*C*‐2,5,7,10,12,15,17,20), 135.78–135.50 (Ar*C*‐28,34,40), 131.49–130.77 (Ar*C*‐27,33,39), 131.20–130.10 (broad, Ar*C*‐3,4,8,9,13,14,18,19), 129.76–129.68 (Ar*C*‐30,36,42), 119.46–119.26 (Ar*C*‐29,35,41), 115.87–115.19 (Ar*C*‐6,11,16), 111.05–110.81 (Ar*C*‐31,37,43), 104.40 (Ar*C*‐1), 55.81 (O*Me*‐46,47,48); UV/Vis (CHCl_3_): λ/nm (log (ε/M^–1^ cm^–1^)) 413 (5.74), 508 (4.25); Accurate mass: *m/z* 629.25449 [M + H]^+^; calcd. for C_41_H_33_N_4_O_3_: 629.25526.

**5‐Bromo‐10,15,20‐tris(2‐methoxyphenyl)porphyrin (4)**. Compound **3** (385.4 mg, 0.61 mmol) was dissolved in chloroform (25 mL) and NBS (128.15 mg, 0.72 mmol) was added. After stirring at r.t. for 25 min the reaction mixture was quenched by the addition a solution of Na_2_SO_3_ (177.33 mg, 1.41 mmol) in water (10 mL). After 15 min of stirring the reaction mixture was diluted with chloroform (50 mL) and washed with water. The organic layer was dried with Na_2_SO_4_, filtered and the solvents evaporated to dryness. The crude product was purified by silica gel column chromatography using DCM/*n*‐heptane (50:50 going to 75:25, (v/v)) as an eluent to yield **4** (467.7 mg, 0.66 mmol, 100 %) as a purple solid containing 3 atropisomers.

^1^H‐NMR (CDCl_3_, 500 MHz): ∂ 9.60 (d, 2H, β‐pyrrole‐*H*‐3,19, *J* = 4.8 Hz), 8.80 (bs, 2H, β‐pyrrole‐*H*‐4,18), 8.68 (bs, 4H, β‐pyrrole‐*H*‐8,9,13,14), 8.03–7.89 (m, 3H, Ar*H*‐28,34,40), 7.79–7.70 (m, 3H, Ar*H*‐30,36,42), 7.38–7.33 (m, 3H, Ar*H*‐29,35,41), 7.33–7.26 (m, 3H, Ar*H*‐31,37,43), 3.62–3.52 (m, 9H, O*Me*‐46,47,48), –2.63 (s, 2H, N*H*); ^13^C{^1^H}‐NMR (CDCl_3_, 126 MHz): ∂ 159.50–159.20 (Ar*C*‐32,38,44), 135.62–135.38 (Ar*C*‐28,34,40), 130.74 (Ar*C*‐27,33,39), 129.91–129.84 (Ar*C*‐30,36,42), 119.41 (Ar*C*‐29,35,41), 116.53–116.29 (Ar*C*‐6,11,16), 110.97–110.77 (Ar*C*‐31,37,43), 102.43 (Ar*C*‐1), 55.79 (O*Me*‐46,47,48); UV/Vis (CHCl_3_): λ/nm (log (ε/M^–1^ cm^–1^)) 421 (5.54), 517 (4.21); Accurate mass: *m/z* 707.16436 (M(^79^Br)+H)^+^, 709.16321 (M(^81^Br)+H)^+^; calcd. for C_41_H_32_BrN_4_O_3_ 707.16578 (^79^Br); 709.16373 (^81^Br); Anal. Calcd for C_41_H_31_BrN_4_O_3_: C, 69.59; H, 4.42; N, 7.92; found C, 69.16; H, 4.34; N, 7.76.

**5‐(5‐Bromo‐2‐methoxyphenyl)‐10,15,20‐tris(2‐methoxyphenyl)porphyrin (5)**. Compound **4** (690 mg, 0.98 mmol), (5‐bromo‐2‐methoxyphenyl)boronic acid (1.125 g, 4.88 mmol), triphenylarsine (119.9 mg, 0.39 mmol), bis(triphenyl‐phosphine)palladium (II) dichloride (137 mg, 0.20 mmol), and tripotassium phosphate (1.035 g, 4.88 mmol) were dissolved in distilled THF (175 mL). The reaction mixture was stirred at 42.5 °C for 4 h (reaction progress monitored with the help of MALDI‐TOF). After cooling to r.t., the mixture was evaporated to dryness and the residue purified by a silica gel column using DCM as the eluent. Subsequently, the crude product was purified by silica gel column chromatography using DCM/*n*‐heptane (60:40 going to 80:20 (v/v)) as the eluent to yield **5** (660.5 mg, 0.81 mmol, 83 %) as a purple solid containing 8 atropisomers.

^1^H‐NMR (CDCl_3_, 500 MHz): ∂ 8.78–8.68 (m, 8H, β‐pyrrole‐*H*‐3,4,8,9,13,14,18,19), 8.20–8.07 (m, 1H, Ar*H*‐22), 8.06–7.91 (m, 3H, Ar*H*‐28,34,40), 7.84 (d, 1H, Ar*H*‐24, *J* = 9.0 Hz), 7.75 (t, 3H, Ar*H*‐30,36,42, *J* = 7.9 Hz), 7.36–7.32 (m, 3H, Ar*H*‐29,35,41), 7.32–7.28 (m, 3H, Ar*H*‐31,37,43), 7.21–7.14 (m, 1H, Ar*H*‐25), 3.63–3.50 (m, 12H, O*Me*‐45,46,47,48), –2.65 (s, 2H, N*H*); ^13^C{^1^H}‐NMR (CDCl_3_, 126 MHz): ∂ 159.46 (Ar*C*‐32,38,44), 158.73 (Ar*C*‐26), 137.96–137.64 (Ar*C*‐22), 135.83–135.40 (Ar*C*‐28,34,40), 133.37 (Ar*C*‐21), 132.38 (Ar*C*‐24), 131.11 (Ar*C*‐27,33,39), 129.72 (Ar*C*‐30,36,42), 119.33 (Ar*C*‐29,35,41), 115.71 (Ar*C*‐6,11,16), 113.44 (Ar*C*‐1), 112.51 (Ar*C*‐25), 111.77 (Ar*C*‐23), 110.91 (Ar*C*‐31,37,43), 56.09 (O*Me*‐45,[46,47,48]), 55.85 (O*Me*‐[45],46,47,48); UV/Vis (CHCl_3_) λ/nm (log (ε/M^–1^ cm^–1^)) 419 (5.62), 514 (4.28); Accurate mass: *m/z* 813.20482 (M(^79^Br)+H)^+^, 815.20403 (M(^81^Br)+H)^+^; calcd. for C_48_H_38_BrN_4_O_4_: 813.20764 (^79^Br); 815.20560 (^81^Br); Anal. Calcd for C_48_H_37_BrN_4_O_4_: C, 70.85; H, 4.58; N, 6.89; found C, 70.55; H, 4.45; N, 6.80.

**5‐(5‐Bromo‐2‐hydroxyphenyl)‐10,15,20‐tris(2‐hydroxyphenyl)porphyrin (6)**. Compound **5** (660.5 mg, 0.81 mmol) was dissolved in distilled DCM (40 mL) and this solution was cooled to –30 °C. Then, BBr_3_ (1.55 mL, 16.2 mmol) was added. The reaction mixture was warmed up to r.t. overnight before it was poured into an ice‐water mixture (100 mL). Ethyl acetate (110 mL) and sat. aq. NaHCO_3_ (250 mL) were added upon which the color of the mixture turned from green to red. The organic layer was washed with sat. aq. NaHCO_3_ (2 × 70 mL) and dried with MgSO_4_, filtered, and the solvents evaporated to dryness. The crude product was purified by silica gel column chromatography using ethyl acetate/chloroform (50:50 (v/v)) as an eluent, yielding **6** (549.4 mg, 0.73 mmol, 89 %) as a purple solid containing 8 atropisomers.

^1^H‐NMR (CDCl_3_, 500 MHz): ∂ 8.96–8.88 (m, 8H, β‐pyrrole‐*H*‐3,4,8,9,13,14,18,19), 8.13–8.08 (m, 1H, Ar*H*‐22), 7.98–7.94 (m, 3H, Ar*H*‐28,34,40), 7.83 (t, 1H, Ar*H*‐24, *J* = 8.8 Hz), 7.73 (t, 3H, Ar*H*‐30,36,42, *J* = 8.1 Hz), 7.37–7.32 (m, 6H, Ar*H*‐29,31,35,37,41,43), 7.23 (d, 1H, Ar*H*‐25, *J* = 2.5 Hz), 4.92 (bs, 4H, O*H*), –2.78 (s, 2H, N*H*); ^13^C{^1^H}‐NMR (CDCl_3_, 126 MHz): ∂ 155.40 (Ar*C*‐32,38,44), 154.72 (Ar*C*‐26), 137.07 (Ar*C*‐22), 135.01 (Ar*C*‐28,34,40), 133.57 (Ar*C*‐24), 133.00–131.00 (broad Ar*C*‐3,4,8,9,13,14,18,19), 130.76 (Ar*C*‐30,36,42), 129.35 (Ar*C*‐21), 127.22 (Ar*C*‐27,33,39), 119.77–119.73 (Ar*C*‐29,35,41), 117.36 (Ar*C*‐25), 115.71–115.55 (Ar*C*‐31,37,43), 114.01–113.73 (Ar*C*‐6,11,16), 111.95–111.91 (Ar*C*‐23), 111.53–111.30 (Ar*C*‐1); UV/Vis (CHCl_3_): λ/nm (log (ε/M^–1^ cm^–1^)) 419 (5.32), 513 (4.20); Accurate mass: *m/z* 757.14343 (M(^79^Br)+H)^+^, 759.14233 (M(^81^Br)+H)^+^; calcd. for C_44_H_30_BrN_4_O_4_: 757.14504 (^79^Br), 759.14300 (^81^Br); Anal. Calcd for C_44_H_29_BrN_4_O_4_: C, 69.75; H, 3.86; N, 7.40; found C, 69.31; H, 3.91; N, 7.13.

**Mono‐bromo porphyrin cage compound (8)**. To compound **7** (549.09 mg, 0.41 mmol), potassium carbonate (1.406 g, 10.19 mmol), compound **6** (306.97 mg, 0.41 mmol), and acetonitrile (750 mL) were added. Argon was led through the reaction mixture for 30 min, and the solution was subsequently refluxed for 16 h. After cooling, the mixture was filtered through Celite and the filtrate was evaporated to dryness. The crude product was purified by column chromatography (alumina Brockmann III) using chloroform as the eluent. Precipitation from DCM/*n*‐heptane followed by washing with *n‐*pentane (4 ×) and drying in vacuo yielded **8** (92.6 mg, 0.07 mmol, 16 %, racemate of two enantiomers) as a purple solid.

^1^H‐NMR (CDCl_3_, 500 MHz): ∂ 8.80–8.74 (m, 4H, β‐pyrrole‐*H*‐3,4,13,14), 8.70–8.64 (m, 4H, β‐pyrrole‐*H*‐8,9,18,19), 8.21 (d, 1H, Ar*H*‐22, *J* = 2.5 Hz), 8.10–8.03 (m, 3H, Ar*H*‐28,34,40), 7.86 (dd, 1H, Ar*H*‐24, *J* = 8.9, 2.5 Hz), 7.78–7.73 (m, 3H, Ar*H*‐30,36,42), 7.41–7.36 (m, 3H, Ar*H*‐29,35,41), 7.35–7.32 (m, 3H, Ar*H*‐31,37,43), 7.21 (d, 1H, Ar*H*‐25, *J* = 8.9 Hz), 6.98–6.91 (m, 6H, Ar*H*‐55,56), 6.83–6.80 (m, 4H, Ar*H*‐54), 6.19 (s, 3H, Ar*H*‐48(II, III, IV)), 6.18 (s, 1H, Ar*H*‐48(*I*)), 4.31–4.23 (m, 4H, C*H*_2_‐45a), 4.23 (d, 4H, C*H*_2_‐50a, *J* = 15.9 Hz), 4.10–4.00 (m, 4H, C*H*_2_‐45b), 3.74 (d, 4H, C*H*_2_‐50b, *J* = 15.6 Hz), 3.55–3.48 (m, 4H, C*H*_2_‐46a), 3.39–3.30 (m, 4H, C*H*_2_‐46b), –2.75 (s, 2H, N*H*); ^13^C{^1^H}‐NMR (CDCl_3_, 126 MHz): ∂ 158.72 (Ar*C*‐32,38,44), 158.01 (Ar*C*‐26), 156.99 (C=O‐51), 146.58–146.49 (Ar*C*‐47), 138.01 (Ar*C*‐22), 135.77 (Ar*C*‐28,34,40), 134.03 (Ar*C*‐21), 133.64 (Ar*C*‐53), 132.34 (Ar*C*‐24), 131.80–131.76 (Ar*C*‐27,33,39), 129.93–129.83 (Ar*C*‐49), 129.64 (Ar*C*‐30,36,42), 128.52–128.45 (Ar*C*‐55,56), 128.11 (Ar*C*‐54), 119.88–119.84 (Ar*C*‐29,35,41), 115.59–115.39 (Ar*C*‐6,11,16), 115.18 (Ar*C*‐48), 113.51 (Ar*C*‐25), 113.16 (Ar*C*‐1), 112.33 (Ar*C*‐23), 111.93–111.87 (Ar*C*‐31,37,43), 84.77 (*C*‐52), 67.43 (*C*H_2_‐46), 66.86 (*C*H_2_‐45), 44.41 (*C*H_2_‐50); UV/Vis (CHCl_3_): λ/nm (log (ε/M^–1^ cm^–1^)) 421 (5.46), 516 (4.18); Accurate mass: *m/z* 1423.39108 (M(^79^Br)+H)^+^, 1425.39106 (M(^81^Br)+H)^+^; calcd. for C_84_H_64_BrN_8_O_10_: 1423.39288 (^79^Br), 1425.39083 (^81^Br).

**Zinc mono‐bromo porphyrin cage compound (9)**. The mono‐bromo porphyrin cage compound (**8**, 220 mg, 0.15 mmol, 1 equiv.) was dissolved in chloroform (15 mL) and methanol (7.5 mL). Zinc(II) acetate dihydrate (119.60 mg, 0.54 mmol, 3.5 equiv.) was added and the reaction mixture was stirred at r.t. for 1 h. After evaporating the mixture to dryness, the residue was purified by silica gel column chromatography using chloroform as the eluent. Precipitation from DCM/*n*‐heptane followed by washing with *n*‐pentane (4 ×) and drying in vacuo yielded the product (**9**, 229.4 mg, 0.15 mmol, 100 %, racemate of two enantiomers) as a pink/purple solid.

^1^H‐NMR (CDCl_3_, 500 MHz): ∂ 8.94–8.87 (m, 4H, β‐pyrrole‐*H*‐3,4,13,14), 8.80–8.73 (m, 4H, β‐pyrrole‐*H*‐8,9,18,19), 8.23 (d, 1H, Ar*H*‐22, *J* = 2.5 Hz), 8.12–8.03 (m, 3H, Ar*H*‐28,34,40), 7.85 (dd, 1H, Ar*H*‐24, *J* = 8.9, 2.6 Hz), 7.78–7.72 (m, 3H, Ar*H*‐30,36,42), 7.40–7.35 (m, 3H, Ar*H*‐29,35,41), 7.35–7.31 (m, 3H, Ar*H*‐31,37,43), 7.21 (d, 1H, Ar*H*‐25, *J* = 8.9 Hz), 6.99–6.90 (m, 6H, Ar*H*‐55,56), 6.77–6.72 (m, 4H, Ar*H*‐54), 6.11 (s, 3H, Ar*H*‐48(II, III, IV)), 6.10 (s, 1H, Ar*H*‐48(*I*)), 4.26–4.16 (m, 4H, C*H*_2_‐45a), 4.09 (d, 4H, C*H*_2_‐50a, *J* = 15.6 Hz), 4.06–3.94 (m, 4H, C*H*_2_‐45b), 3.66 (d, 4H, C*H*_2_‐50b, *J* = 15.8 Hz), 3.55–3.46 (m, 4H, C*H*_2_‐46a), 3.32–3.23 (m, 4H, C*H*_2_‐46b); ^13^C{^1^H}‐NMR (CDCl_3_, 126 MHz): ∂ 158.75 (Ar*C*‐32,38,44), 158.04 (Ar*C*‐26), 156.84 (O=C‐51), 150.23–149.45 (Ar*C*‐2,5,7,10,12,15,17,20), 146.51–146.42 (Ar*C*‐47), 137.89 (Ar*C*‐22), 135.56 (Ar*C*‐28,34,40), 134.78 (Ar*C*‐21), 133.58 (Ar*C*‐53), 132.53 (Ar*C*‐27,33,39), 132.10 (Ar*C*‐24), 131.70–130.50 (Ar*C*‐3,4,8,9,13,14,18,19), 129.89–129.79 (Ar*C*‐49), 129.41 (Ar*C*‐30,36,42), 128.54–128.46 (Ar*C*‐55,56), 128.05 (Ar*C*‐54), 119.85 (Ar*C*‐29,35,41), 116.51–116.28 (Ar*C*‐6,11,16), 115.19 (Ar*C*‐48), 114.11 (Ar*C*‐1), 113.68 (Ar*C*‐25), 112.33 (Ar*C*‐23), 112.11–112.04 (Ar*C*‐31,37,43), 84.68 (*C*‐52), 67.45 (*C*H_2_‐46), 66.94 (*C*H_2_‐45), 44.26 (*C*H_2_‐50); UV/Vis (CHCl_3_) λ/nm (log (ε/M^–1^ cm^–1^)) 422 (5.64), 549 (4.29); MALDI‐TOF; *m/z*: 1485.3 (M(^79^Br)+H)^+^, 1487.3 (M(^81^Br)+H)^+^; calcd. for C_84_H_62_BrN_8_O_10_Zn 1485.3 (^79^Br); 1487.3 (^81^Br).

**Potassium triisopropylsilylacetylene trifluoroborate (TIPSA BF_3_K)**. This compound was synthesized according to a modified literature procedure.^[^ ^16^ ^]^A solution of triisopropylsilylacetylene (2.0 mL, 8.9 mmol) was dissolved in degassed THF (20 mL) and cooled to –78 °C. *n*‐Butyllithium (5.6 mL, 8.9 mmol) was added dropwise and the reaction mixture was stirred for 1 hour. Trimethoxyborate (1.5 mL, 13 mmol) was added dropwise, the mixture was stirred for 1 hour and then warmed up to –20 °C over the course of 1 hour. Subsequently, a saturated solution of potassium bifluoride (4.30 g, 55.1 mmol) in water was added under vigorous stirring. The mixture was stirred at –20 °C for 1 hour, after which it was warmed to room temperature. The solvent was evaporated and the resulting white residue was dried under vacuum for 2 hours. The residue was washed with subsequently acetone and hot acetone and the filtrate was evaporated to yield a white solid, which was re‐precipitated from hot acetone/petroleum ether (80–100). After cooling the suspension to –20 °C, the precipitate was filtered off and the residue was washed with *n*‐pentane. After drying in air and under vacuum, the product was obtained as a white, fluffy solid (1.0 g, 3.6 mmol, 40 %).

^1^H‐NMR ([D_6_]Acetone, 400 MHz): ∂ 1.08–1.10 (m, 18H, CH(C*H*_3_)_2_), 0.93–1.03 (m, 3H, C*H*(CH_3_)_2_); ^19^F‐NMR ([D_6_]acetone, 380 MHz): ∂ 135.5 (1:1:1:1, *J* = 34 Hz); ^29^Si‐NMR ([D_6_]acetone, 80 MHz): ∂ (ppm) –5.63 (s); ^11^B‐NMR ([D_6_]acetone, 133 MHz) δ –2.15 (q, *J* = 36 Hz).

**TIPS‐protected acetylene functionalized zinc porphyrin cage compound 10**. Compound **9** (288 mg, 0.19 mmol), potassium triisopropylsilylacetylene trifluoroborate (111.7 mg, 0.39 mmol), cesium carbonate (315.8 mg, 0.97 mmol), and [1,1'‐bis(diphenylphosphino) ferrocene]dichloropalladium(II) (39.8 mg, 0.05 mmol) were dissolved in THF (42.75 mL) and water (2.25 mL) under an argon atmosphere. The reaction mixture was refluxed for 16 h. After cooling to r.t., the mixture was diluted with DCM and the solution was subsequently washed with water (4 × 50 mL). The organic layer was dried with MgSO_4_, filtered, and the solvents evaporated to dryness. The crude product was purified by silica gel column chromatography using 1 % MeOH in CHCl_3_ (v/v) as the eluent yielding **10** (285.4 mg, 0.18 mmol, 93 %, racemate of two enantiomers) as a pink/purple solid.

^1^H‐NMR (CDCl_3_, 500 MHz): ∂ 8.93–8.85 (m, 4H, β‐pyrrole‐H‐3,4,13,14), 8.74–8.67 (m, 4H, β‐pyrrole‐H‐8,9,18,19), 8.19 (d, 1H, Ar*H*‐22, *J* = 2.1 Hz), 8.01–7.96 (m, 2H, Ar*H*‐28,40), 7.95–7.91 (m, 1H, Ar*H*‐34), 7.87 (dd, 1H, Ar*H*‐24, *J* = 8.5, 2.1 Hz), 7.74–7.68 (m, 3H, Ar*H*‐30,36,42), 7.33–7.29 (m, 3H, Ar*H*‐31,37,43), 7.29–7.24 (m, 3H, Ar*H*‐29,35,41), 7.22 (d, 1H, Ar*H*‐25, *J* = 8.6 Hz), 6.97–6.91 (m, 6H, Ar*H*‐55,56), 6.44 (bs, 4H, Ar*H*‐54), 5.86–5.73 (m, 4H, Ar*H*‐48), 4.17–4.07 (m, 4H, C*H*_2_‐45a), 3.95–3.84 (m, 4H, C*H*_2_‐45b), 3.55 (bs, 4H, C*H*_2_‐50a), 3.47–3.39 (m, 4H, C*H*_2_‐46a), 3.39–3.29 (m, 4H, C*H*_2_‐50b), 3.17–3.05 (m, 4H, C*H*_2_‐46b), 1.07 (bs, 21H, TIPS); ^13^C{^1^H}‐NMR (CDCl_3_, 126 MHz): ∂ 159.05 (Ar*C*‐26), 158.84–158.81 (Ar*C*‐32,44), 158.76 (Ar*C*‐38), 156.40–156.37 (C=O‐51), 150.10–149.60 (Ar*C*‐2,5,7,10,12,15,17,20), 146.32–146.08 (Ar*C*‐47), 138.60 (Ar*C*‐22), 135.54 (Ar*C*‐28,40), 135.39 (Ar*C*‐34), 133.51 (Ar*C*‐24), 133.23 (Ar*C*‐53), 132.85 (Ar*C*‐21), 132.79–132.75 (Ar*C*‐27,33,39), 131.50–130.50 (Ar*C*‐3,4,8,9,13,14,18,19), 129.37 (Ar*C*‐49), 129.31–129.22 (Ar*C*‐30,36,42), 128.51 (Ar*C*‐55,56), 127.86 (Ar*C*‐54), 119.94–119.82 (Ar*C*‐29,35,41), 116.07 (Ar*C*‐11), 115.91–115.88 (Ar*C*‐6,16), 114.87 (Ar*C*‐23), 114.78 (Ar*C*‐48), 114.38 (Ar*C*‐1), 112.39–112.27 (Ar*C*‐31,37,43), 111.92 (Ar*C*‐25), 107.10 (Ar*C*‐57), 88.95 (Ar*C*‐58), 84.37 (*C*‐52), 67.37–67.19 (*C*H_2_‐46), 67.02–66.96 (*C*H_2_‐45), 43.76 (*C*H_2_‐50), 18.69 (TIPS‐*C*), 11.33 (TIPS‐*C*); MALDI‐TOF: *m/z* 1587.5 [M + H]^+^; calcd. for C_95_H_83_N_8_O_10_SiZn: 1587.5.

**Acetylene functionalized zinc porphyrin cage compound 11**. Argon was led through THF (75 mL) for 30 min and compound **10** (372 mg, 0.23 mmol) and tetrabutylammonium fluoride trihydrate (739.5 mg, 2.34 mmol) were added. The reaction mixture was stirred in the dark at r.t. for 4 h and was then evaporated to dryness. The residue was dissolved in DCM and this solution was washed with water (3 × 50 mL), dried with Na_2_SO_4_, filtered and the solvents evaporated to dryness. The crude product was purified by silica gel column chromatography using 0.5 % MeOH in CHCl_3_ (v/v) as the eluent. Precipitation from DCM/*n*‐heptane followed by washing with *n*‐pentane (4 ×) and drying in vacuo yielded **11** (244 mg, 0.17 mmol, 73 %, racemate of two enantiomers) as a pink/purple solid.

^1^H‐NMR (CDCl_3_, 500 MHz): ∂ 8.91–8.87 (m, 4H, β‐pyrrole‐*H*‐3,4,13,14), 8.77–8.71 (m, 4H, β‐pyrrole‐*H*‐8,9,18,19), 8.24 (d, 1H, Ar*H*‐22, *J* = 2.2 Hz), 8.10–8.03 (m, 3H, Ar*H*‐28,34,40), 7.89 (dd, 1H, Ar*H*‐24, *J* = 8.6, 2.2 Hz), 7.77–7.72 (m, 3H, Ar*H*‐30,36,42), 7.38–7.34 (m, 3H, Ar*H*‐29,35,41), 7.34–7.32 (m, 3H, Ar*H*‐31,37,43), 7.27 (d, 1H, Ar*H*‐25, *J* = 8.7 Hz), 6.97–6.92 (m, 6H, Ar*H*‐55,56), 6.74–6.68 (m, 4H, Ar*H*‐54), 6.09–6.05 (m, 4H, Ar*H*‐48), 4.28–4.15 (m, 4H, C*H*_2_‐45a), 4.07–3.96 (m, 8H, C*H*_2_‐45b, C*H*_2_‐50a), 3.63 (d, 4H, C*H*_2_‐50b, *J* = 16.0 Hz), 3.54–3.47 (m, 4H, C*H*_2_‐46a), 3.32–3.22 (m, 4H, C*H*_2_‐46b), 3.05 (s, 1H, alkyne*H*‐58); ^13^C{^1^H}‐NMR (CDCl_3_, 126 MHz): ∂ 159.22 (Ar*C*‐26), 158.80–158.74 (Ar*C*‐32,38,44), 156.80 (C=O‐51), 150.18–149.55 (Ar*C*‐2,5,7,10,12,15,17,20), 146.49–146.35 (Ar*C*‐47), 139.07 (Ar*C*‐22), 135.58–135.59 (Ar*C*‐28,34,40), 133.54 (Ar*C*‐24), 133.50 (Ar*C*‐53), 132.81 (Ar*C*‐21), 132.67–132.59 (Ar*C*‐27,33,39), 131.60–130.56 (Ar*C*‐3,4,8,9,13,14,18,19), 129.83–129.67 (Ar*C*‐49), 129.36 (Ar*C*‐30,36,42), 128.53–128.47 (Ar*C*‐55,56), 128.04 (Ar*C*‐54), 119.85 (Ar*C*‐29,35,41), 116.32–116.13 (Ar*C*‐6,11,16), 115.16–114.97 (Ar*C*‐48), 114.46 (Ar*C*‐1), 113.37 (Ar*C*‐23), 112.18–112.07 (Ar*C*‐31,37,43), 111.70 (Ar*C*‐25), 84.66 (*C*‐52), 83.76 (alkyne*C*‐57), 76.12 (alkyne*C*‐58), 67.45–67.40 (*C*H_2_‐46), 66.95–66.93 (*C*H_2_‐45), 44.21 (*C*H_2_‐50); MALDI‐TOF: *m/z* 1431.3 [M + H]^+^; calcd. for C_86_H_63_N_8_O_10_Zn: 1431.4.

**1,3‐Diazidopropane**. This compound was synthesized according to a modified literature procedure.^[^ ^17^ ^]^ Sodium azide‐1‐^15^N (490 mg, 7.43 mmol) and 1,3‐dibromopropane (0.25 mL, 2.48 mmol) were dissolved in DMF (16 mL) and the reaction mixture was heated at 85 °C for 20 h. After cooling to r.t., water (100 mL) was added to the mixture and the resulting solution was extracted with diethyl ether. Subsequently the organic layer was washed with water (5 × 100 mL) and brine (2 × 100 mL), dried with Na_2_SO_4_, filtered, and evaporated to almost dryness to yield the product as a clear solution in diethyl ether (96 %, based on ^1^H NMR integrals). Due to its instability the product was directly used in the following synthesis step.

^1^H NMR (CDCl_3_, 500 MHz): ∂ 3.42 (t, 4H, N_3_C*H*_2_CH_2_C*H*_2_N_3_, *J* = 6.5 Hz), 1.87–1.80 (m, 2H, N_3_CH_2_C*H*_2_CH_2_N_3_); ^13^C NMR (CDCl_3_, 126 MHz): ∂ 48.58 (N_3_ *C*H_2_CH_2_ *C*H_2_N_3_), 28.47 (N_3_CH_2_ *C*H_2_CH_2_N_3_); ^15^N NMR^†^ (CDCl_3_, 51 MHz): ∂ 247.78 (s, N*NN*CH_2_CH_2_CH_2_N*NN*), 211.36 (s, N*NN*CH_2_CH_2_CH_2_N*NN*), 69.69 (s, N*NN*CH_2_CH_2_CH_2_ N*NN*); ^†^50 % of the indicated N atoms are ^15^N‐labeled.

**1,5‐Diazidopentane**. This compound was synthesized according to a modified literature procedure.^[^ ^17^ ^]^ Sodium azide‐1‐^15^N (486 mg, 7.37 mmol) and 1,5‐dibromopentane (0.35 mL, 2.57 mmol) were dissolved in DMF (15 mL) and the reaction mixture was heated at 80 °C for 16 h. After cooling to r.t., water (100 mL) was added to the mixture and the resulting solution was extracted with diethyl ether (2 × 150 mL). Subsequently the organic layer was washed with brine (2 × 100 mL), dried with Na_2_SO_4_, filtered and the evaporated to almost dryness to yield the product as a clear solution in diethyl ether (75 %, based on ^1^H NMR integrals). Due to its instability the product was directly used in the following synthesis step.

^1^H NMR (CDCl_3_, 500 MHz): ∂ 3.29 (t, 4H, N_3_C*H*_2_(CH_2_)_3_C*H*_2_N_3_, *J* = 6.8 Hz), 1.60–1.52 (m, 4H, N_3_CH_2_C*H*_2_CH_2_C*H*_2_CH_2_N_3_), 1.45–1.35 (m, 2H, N_3_(CH_2_)_2_C*H*_2_(CH_2_)_2_N_3_); ^15^N NMR^†^ (CDCl_3_, 51 MHz): ∂ 242.94 (s, N*NN*CH_2_(CH_2_)_3_CH_2_N*NN*), 205.87 (s, N*NN*CH_2_(CH_2_)_5_CH_2_N*NN*), 66.78 (s, N*NN*CH_2_(CH_2_)_3_CH_2_ N*NN*); ^†^50 % of the indicated N atoms are ^15^N‐labeled.

**1,11‐Diazidoundecane**. This compound was synthesized according to a modified literature procedure.^[^ ^17^ ^]^ Sodium azide‐1‐^15^N (500 mg, 7.58 mmol, 3 equiv.) and 1,11‐dibromoundecane (0.60 mL, 2.53 mmol, 1 equiv.) were dissolved in DMF (15 mL). The reaction mixture was stirred at 80 °C for 20 h. After cooling to r.t., water (150 mL) was added to the mixture and the resulting solution was extracted with diethyl ether (2 × 100 mL). Subsequently, the organic layer was washed with water (10 × 200 mL) and brine (2 × 200 mL), dried with Na_2_SO_4_, filtered and evaporated to almost dryness to yield the product as a clear solution in diethyl ether (45 %, based on ^1^H NMR integrals). Due to its instability the product was directly used in the following synthesis step.

^1^H‐NMR (CDCl_3_, 500 MHz): ∂ 3.26 (t, 4H, N_3_C*H*_2_(CH_2_)_9_C*H*_2_N_3_, *J* = 7 Hz), 1.62–1.56 (m, 4H, N_3_CH_2_C*H*_2_(CH_2_)_7_C*H*_2_CH_2_N_3_), 1.39–1.28 (m, 14H, N_3_(CH_2_)_2_ *(*C*H*_2_ *)*_7_(CH_2_)_2_N_3_); ^13^C NMR (CDCl_3_, 126 MHz): ∂ 51.49 & 51.46 (N_3_ *C*H_2_(CH_2_)_9_ *C*H_2_N_3_), 29.44 & 29.41 (N_3_(CH_2_)_3_(*C*H_2_)_5_(CH_2_)_3_N_3_),, 28.8 (N_3_CH_2_ *C*H_2_(CH_2_)_7_ *C*H_2_CH_2_N_3_), 26.7 (N_3_(CH_2_)_2_ *C*H_2_(CH_2_)_5_ *C*H_2_(CH_2_)_2_N_3_; ^15^N NMR^†^ (CDCl_3_, 51 MHz): *δ* = 248.5 (s, N*NN*CH_2_(CH_2_)_9_CH_2_N*NN*), 210.5 (s, N*NN*CH_2_(CH_2_)_9_CH_2_N*NN*), 71.9 (s, N*NN*CH_2_(CH_2_)_9_CH_2_ N*NN*); ^†^50 % of the indicated N atoms are ^15^N‐labeled.

**Double porphyrin cage compound Zn_2_C_3_DC**. To a mixture of compound **11** (135.5 mg, 0.09 mmol) and copper(I) iodide (9.36 mg, 0.05 mmol) in freshly distilled THF (19 mL) and freshly distilled MeCN (19 mL), DIPEA (15.80 µL, 0.09 mmol) was added. A solution of 1,3‐diazidopropane‐^15^N‐enriched in diethyl ether (74.3 µL, 0.57 m, 0.04 mmol) was added and the reaction mixture was stirred in the dark at r.t. for 9 days. On days 2 and 4 additional CuI (12.08 mg and 21.15 mg, resp.) and DIPEA (2 × 15.80 µL) were added. The mixture was diluted with DCM (50 mL) and washed with water (3 × 50 mL). The organic layer was evaporated and the crude product was purified by silica gel column chromatography using a gradient of 1–5 % of MeOH in CHCl_3_ (v/v) as the eluent. Precipitation from DCM/*n*‐heptane followed by washing with *n*‐pentane (4×) and drying in vacuo yielded **Zn_2_C_3_DC** (44.8 mg, 0.02 mmol, 35 %) as a pink/purple solid.

^1^H‐NMR ([D_6_]DMSO at 333.15 K, 500 MHz): ∂ 8.79 (dd, 2H, β‐pyrrole‐*H, J* = 4.6, 1.2 Hz), 8.75–8.70 (m, 6H, β‐pyrrole‐*H*), 8.58–8.49 (m, 8H, β‐pyrrole‐*H*) 8.50–8.47 (m, 2H, Triazole‐*H*‐58), 8.33 (t, 2H, Ar*H*‐22, *J* = 2.4 Hz), 8.19 (dd, 1H, Ar*H*‐24*, *J* = 8.5, 2.3 Hz), 8.18 (dd, 1H, Ar*H*‐24*, *J* = 8.5, 2.3 Hz) 7.92–7.82 (m, 6H, Ar*H*‐28, 34, 40), 7.79–7.66 (m, 6H, Ar*H*‐30, 36, 42), 7.56–7.48 (m, 6H, Ar*H*‐31, 37, 43), 7.46 (d, 1H, Ar*H*‐25*, *J* = 8.7 Hz), 7.39 (d, 1H, Ar*H*‐25*, *J* = 8.9 Hz), 7.37–7.19 (m, 6H, Ar*H*‐29, 35, 41), 7.05–6.94 (m, 12H, Ar*H*‐55, 56), 6.84–6.78 (m, 6H, Ar*H*‐48(II,III,IV)), 6.20 (s, 1H, Ar*H*‐48(*I*)*), 6.19 (s, 1H, Ar*H*‐48(*I*)*), 4.43 (t, 4H, C*H*_2_‐62, *J* = 6.8 Hz), 4.22–4.03 (m, 24H, C*H*_2_‐45, C*H*_2_‐50a), 3.65 (d, 8H, C*H*_2_‐50b, *J* = 15.7 Hz), 3.51–3.40 (m, 8H, C*H*_2_‐46a), 3.28–3.22 (m, 8H, C*H*_2_‐46b), 2.51–2.46 (m, 2H, C*H*_2_‐63); ^13^C NMR^‡^ ([D_6_]DMSO at 333.15 K, 126 MHz): ∂ 158.40 (Ar*C*‐32, 38, 44), 156.00 (*C*=O‐51), 155.86 (*C*=O‐51), 149.37 (Ar*C*‐2, 5, 7, 10, 12, 15, 17, 20), 148.57 (Ar*C*‐2, 5, 7, 10, 12, 15, 17, 20), 146.07 (*C*‐57), 145.69 (Ar*C*‐47), 134.53 (Ar*C*‐28, 34, 40), 133.06 (Ar*C*‐53), 132.40 (Ar*C*‐27, 33, 39), 131.70 (Ar*C*‐22), 130.21 (Ar*C*‐3, 4, 8, 9, 13, 14, 18, 19), 129.89 (Ar*C*‐3, 4, 8, 9, 13, 14, 18, 19), 129.81 (Ar*C*‐49), 128.83 (Ar*C*‐30, 36, 42), 127.79 (Ar*C*‐55, 56), 127.40 (Ar*C*‐54), 125.67 (Ar*C*‐24), 120.44 (*C*H‐58), 119.16 (Ar*C*‐29, 35, 41), 114.79 (Ar*C*‐48(II,III,IV)), 114.46 (Ar*C*‐48(*I*)), 112.62 (Ar*C*‐25), 112.60 (Ar*C*‐31, 37, 43), 84.00 (*C*‐52), 83.83 (*C*‐52), 67.06 (*C*H_2_‐46), 66.66 (*C*H_2_‐45), 46.44 (*C*H_2_‐62), 43.18 (*C*H_2_‐50), 29.28 (*C*H_2_‐63); ^15^N{^1^H} NMR^†^ ([D_6_]DMSO at 333.15 K, 51 MHz): ∂ 346.64 (Triazole‐*N*‐61*), 346.56 (Triazole‐*N*‐61*), 248.62 (Triazole‐*N*‐59*), 248.57 (Triazole‐*N*‐59*); UV/Vis (CHCl_3_/CH_3_CN 1:1, v/v) λ/nm (log (ε/M^–1^ cm^–1^)) 426 (5.71), 559 (4.34), 597 (3.91); Accurate MALDI‐TOF: *m/z* 2988.7613 (M)^+^; calcd. for C_175_H_130_N_20_^15^N_2_O_20_^64^Zn_2_: 2988.8355. *Peaks belonging to the two different diastereoisomers that could not be separated. ^‡13^C‐NMR shifts obtained via HSQC and HMBC spectra as no ^13^C{^1^H}‐NMR spectrum was recorded. ^†^50 % of the indicated N atoms are ^15^N‐labeled. For UV/Vis spectra, see text.

**Double porphyrin cage compound Zn_2_C_5_DC**. To a mixture of compound **11** (134.1 mg, 0.09 mmol) and copper(I) iodide (10.2 mg, 0.05 mmol) in freshly distilled THF (19 mL) and freshly distilled MeCN (19 mL), DIPEA (15.80 µL, 0.09 mmol) was added. A solution of 1,5‐diazidopentane‐^15^N‐enriched in diethyl ether (51.2 µL, 0.83 m, 0.04 mmol) was added and the reaction mixture was stirred in the dark at r.t. for 7 days. On days 2 and 4 additional CuI (20.26 mg and 10.44 mg, resp.) and DIPEA (2 × 15.80 µL) were added. The mixture was concentrated in vacuo and the crude product was purified by silica gel column chromatography using a gradient of 0.7–5 % MeOH in CHCl_3_ (v/v) as the eluent. Precipitation from DCM/*n*‐heptane followed by washing with *n*‐pentane (4 ×) and drying in vacuo yielded **Zn_2_C_5_DC** (39.7 mg, 0.01 mmol, 31 %) as a pink/purple solid.

^1^H‐NMR ([D_6_]DMSO at 333.15 K, 500 MHz): ∂ 8.80 (dd, 2H, β‐pyrrole‐*H, J* = 4.7, 3.5 Hz), 8.76–8.72 (m, 6H, β‐pyrrole‐*H*), 8.59 (d, 2H, β‐pyrrole‐*H, J* = 4.6 Hz) 8.55–8.52 (m, 6H, β‐pyrrole‐*H*), 8.48–8.45 (m, 2H, Triazole‐*H*‐58), 8.34 (t, 2H, Ar*H*‐22, *J* = 1.9 Hz), 8.20 (dd, 2H, Ar*H*‐24, *J* = 8.6, 2.2 Hz), 7.91–7.86 (m, 6H, Ar*H*‐28, 34, 40), 7.79–7.67 (m, 6H, Ar*H*‐30, 36, 42), 7.54–7.46 (m, 6H, Ar*H*‐31, 37, 43), 7.44 (dd, 2H, Ar*H‐*25*, *J* = 8.8, 5.9 Hz), 7.38–7.27 (m, 6H, Ar*H*‐29, 35, 41), 7.04–6.95 (m, 12H, Ar*H*‐55, 56), 6.83–6.78 (m, 8H, Ar*H*‐54), 6.27–6.23 (m, 6H, Ar*H*‐48(II,III,IV)), 6.18 (s, 1H, Ar*H*‐48(*I*)*), 6.16 (s, 1H, Ar*H*‐48(*I*)*), 4.41 (t, 4H, C*H*_2_‐62, *J* = 6.9 Hz), 4.20–4.00 (m, 24H, C*H*_2_‐45, C*H*_2_‐50a), 3.68–3.57 (m, 8H, C*H*_2_‐50b), 3.53–3.40 (m, 8H, C*H*_2_‐46a), 3.27–3.20 (m, 8H, C*H*_2_‐46b), 1.85 (p, 4H, C*H*_2_‐63, *J* = 7.3 Hz), 1.30–1.22 (m, 2H, C*H*_2_‐64); ^13^C NMR^‡^ ([D_6_]DMSO at 333.15 K, 126 MHz): ∂ 158.27 (Ar*C*‐32, 38, 44), 157.92 (Ar*C*‐26), 155.88 (*C*=O‐51), 155.72 (*C*=O‐51), 149.20 (Ar*C*‐2, 5, 7, 10, 12, 15, 17, 20), 148.47 (Ar*C*‐2, 5, 7, 10, 12, 15, 17, 20), 145.84 (*C*‐57), 145.55 (Ar*C*‐47(II, III, IV)), 145.42 (Ar*C*‐47(*I*)), 134.60 (Ar*C*‐28, 34, 40), 132.92 (Ar*C*‐53), 132.42 (Ar*C*‐21), 132.24 (Ar*C*‐27, 33, 39), 131.75 (Ar*C*‐22), 130.28 (Ar*C*‐3, 4, 8, 9, 13, 14, 18, 19), 129.91 (Ar*C*‐3, 4, 8, 9, 13, 14, 18, 19), 129.66 (Ar*C*‐49(II, III, IV)), 129.51 (Ar*C*‐49(*I*)), 128.89 (Ar*C*‐30, 36, 42), 127.84 (Ar*C*‐55, 56), 127.41 (Ar*C*‐54), 125.68 (Ar*C*‐24), 122.16 (Ar*C*‐23), 120.17 (*C*H‐58), 119.21 (Ar*C*‐29, 35, 41), 115.13 (Ar*C*‐6, 11, 16), 114.82 (Ar*C*‐48(II,III,IV)), 114.67 (Ar*C*‐1), 114.47 (Ar*C*‐48(*I*)*), 114.36 (Ar*C*‐48(*I*)*), 112.67 (Ar*C*‐25, 31, 37, 43), 83.83 (*C*‐52), 83.71 (*C*‐52), 67.07 (*C*H_2_‐46), 66.69 (*C*H_2_‐45), 48.68 (*C*H_2_‐62), 43.19 (*C*H_2_‐50), 28.33 (*C*H_2_‐64), 28.27 (*C*H_2_‐63); ^15^N{^1^H} NMR^†^ ([D_6_]DMSO at 333.15 K, 51 MHz): ∂ 345.74 (Triazole‐*N*‐61*), 345.72 (Triazole‐*N*‐61*), 250.74 (Triazole‐*N*‐59*), 250.66 (Triazole‐*N*‐59*); UV/Vis (CHCl_3_/CH_3_CN 1:1, v/v) λ/nm (log (ε/M^–1^ cm^–1^)) 426 (5.95), 558 (4.57), 598 (3.94); Accurate MALDI‐TOF: *m/z* 3016.8603 (M)^+^; calcd. for C_177_H_134_N_20_^15^N_2_O_20_^64^Zn_2_: 3016.8668. *Peaks belonging to the two different diastereoisomers that could not be separated. ^‡13^C‐NMR shifts obtained via HSQC and HMBC spectra as no ^13^C{^1^H}‐NMR spectrum was recorded. ^†^50 % of the indicated N atoms are ^15^N‐labeled.

**Double porphyrin cage compound Zn_2_C_11_DC**. To a solution of compound **11** (134.8 mg, 0.09 mmol) and copper(I) iodide (11.4 mg, 0.06 mmol) in freshly distilled THF (19 mL) and freshly distilled MeCN (19 mL), DIPEA (15.80 µL, 0.09 mmol) was added. A solution of 1,11‐diazidoundecane‐^15^N‐enriched in diethyl ether (304 µL, 0.14 m, 0.04 mmol) was added and the reaction mixture was stirred in the dark at r.t. for 6 days. On days 3 and 5 additional CuI (16.78 mg and 19.24 mg, resp.) and DIPEA (2 × 15.80 µL) were added. The mixture was concentrated in vacuo and the crude products was purified by silica gel column chromatography using a gradient of 0.5 %‐5 % MeOH in CHCl_3_ (v/v) as the eluent. Precipitation from DCM/*n*‐heptane followed by washing with *n*‐pentane (4 ×) and drying in vacuo yielded **Zn_2_C_11_DC** (81.9 mg, 0.03 mmol, 62 %) as a pink/purple solid.

^1^H‐NMR ([D_6_]DMSO at 333.15 K, 500 MHz): ∂ 8.80 (dd, 2H, β‐pyrrole‐*H, J* = 4.6, 2.1 Hz), 8.76–8.70 (m, 6H, β‐pyrrole‐*H*), 8.59 (t, 2H, β‐pyrrole‐*H, J* = 4.2 Hz) 8.56–8.49 (m, 6H, β‐pyrrole‐*H*), 8.47–8.44 (m, 2H, Triazole‐*H*‐58), 8.36–8.34 (m, 2H, Ar*H*‐22), 8.23 (dt, 2H, Ar*H*‐24, *J* = 8.7, 1.7 Hz), 7.92–7.88 (m, 6H, Ar*H*‐28, 34, 40), 7.79–7.68 (m, 6H, Ar*H*‐30, 36, 42), 7.56 (d, 2H, Ar*H*‐25, *J* = 8.8 Hz), 7.54–7.41 (m, 6H, Ar*H*‐31, 37, 43), 7.38–7.27 (m, 6H, Ar*H*‐29, 35, 41), 7.04–6.95 (m, 12H, Ar*H*‐55, 56), 6.83–6.79 (m, 8H, Ar*H*‐54), 6.26–6.20 (m, 8H, Ar*H*‐48), 4.24 (t, 4H, C*H*_2_‐62, *J* = 6.9 Hz), 4.21–4.04 (m, 24H, C*H*_2_‐45, C*H*_2_‐50a), 3.69–3.59 (m, 8H, C*H*_2_‐50b), 3.53–3.38 (m, 8H, C*H*_2_‐46a), 3.29–3.18 (m, 8H, C*H*_2_‐46b), 1.79–1.71 (m, 4H, C*H*_2_‐63), 1.19–1.11 (m, 14H, C*H*_2_‐64, 65, 66, 67); ^13^C NMR^‡^ ([D_6_]DMSO at 333.15 K, 126 MHz): ∂ 158.56 (Ar*C*‐32, 38, 44), 158.23 (Ar*C*‐26), 156.16 (*C*=O‐51), 156.03 (*C*=O‐51), 149.51 (Ar*C*‐2, 5, 7, 10, 12, 15, 17, 20), 148.75 (Ar*C*‐2, 5, 7, 10, 12, 15, 17, 20), 146.13 (*C*‐57), 145.80 (Ar*C*‐47), 134.57 (Ar*C*‐28, 34, 40), 133.23 (Ar*C*‐53), 132.81 (Ar*C*‐21), 132.56 (Ar*C*‐27, 33, 39), 131.70 (Ar*C*‐22), 130.23 (Ar*C*‐3, 4, 8, 9, 13, 14, 18, 19), 129.93 (Ar*C*‐49), 129.87 (Ar*C*‐3, 4, 8, 9, 13, 14, 18, 19), 128.84 (Ar*C*‐30, 36, 42), 127.79 (Ar*C*‐55, 56), 127.34 (Ar*C*‐54), 125.56 (ArC‐24), 122.52 (Ar*C*‐23), 120.10 (*C*H‐58), 119.16 (Ar*C*‐29, 35, 41), 115.52 (Ar*C*‐6, 11, 16), 114.70 (Ar*C*‐48), 112.66 (Ar*C*‐25, 31, 37, 43), 84.08 (*C*‐52), 67.02 (*C*H_2_‐46), 66.61 (*C*H_2_‐45), 48.93 (*C*H_2_‐62), 43.20 (*C*H_2_‐50), 28.84 (*C*H_2_‐63), 28.08 (*C*H_2_‐62), 28.02 (*C*H_2_‐64), 27.67 (*C*H_2_‐65), 25.18 (*C*H_2_‐66, 67); ^15^N{^1^H} NMR^†^ ([D_6_]DMSO at 333.15 K, 51 MHz): ∂ 345.55 (Triazole‐*N*‐61), 251.23 (Triazole‐*N*‐59); UV/Vis (CHCl_3_/CH_3_CN 1:1, v/v) λ/nm (log (ε/M^–1^ cm^–1^)) 426 (5.93), 559 (4.56), 597 (4.03); Accurate MALDI‐TOF: *m/z* 3100.9959 (M)^+^; calcd. for C_183_H_146_N_20_^15^N_2_O_20_^64^Zn_2_: 3100.9607. *Peaks belonging to the two different diastereoisomers that could not be separated. ^‡13^C‐NMR shifts obtained via HSQC and HMBC spectra as no ^13^C{^1^H}‐NMR spectrum was recorded. ^†^50 % of the indicated N atoms are ^15^N‐labeled.

**Double porphyrin cage compound H_4_C_3_DC**. Compound **Zn_2_C_3_DC** (65.93 mg, 22 µmol) was dissolved in CHCl_3_ (100 mL) after which aq. HCl (6 m, 200 mL) was added and the reaction mixture was stirred at r.t. for 2 h. Subsequently, the organic layer was washed with sat. aq. NaHCO_3_ (200 mL) and water (200 mL), dried with Na_2_SO_4_, filtered, and the solvents evaporated to dryness. The crude product was purified by silica gel column chromatography using 1.5 % MeOH in CHCl_3_ (v/v) as the eluent. Precipitation from DCM/*n*‐heptane followed by washing with *n*‐pentane (4 ×) and drying in vacuo yielded **H_4_C_3_DC** (43.16 mg, 15 µmol, 68 %) as a purple solid.

^1^H‐NMR (CDCl_3_, 500 MHz): ∂ 8.80–8.76 (m, 4H, β‐pyrrole‐*H*), 8.76–8.70 (m, 4H, β‐pyrrole‐*H*), 8.69–8.65 (m, 4H, β‐pyrrole‐*H*), 8.65–8.59 (m, 4H, β‐pyrrole‐*H*), 8.40 (dd, 2H, Ar*H*‐22, *J* = 4.3, 2.4 Hz), 8.28–8.21 (m, 2H, Ar*H*‐24), 8.08–7.97 (m, 6H, Ar*H*‐28, 34, 40), 7.79–7.75 (m, 2H, Triazole‐*H*‐58), 7.76–7.63 (m, 6H, ArH‐30, 36, 42), 7.38–7.22 (m, 12H, Ar*H*‐29, 31, 35, 37, 41, 43), 7.26 (1H, Ar*H*‐25^#^), 7.20 (1H, Ar*H*‐25^◊^), 7.01–6.90 (m, 12H, Ar*H*‐55, 56), 6.85–6.77 (m, 8H, Ar*H*‐54), 6.23–6.17 (m, 6H, Ar*H*‐48(II,III,IV)), 6.16 (s, 1H, Ar*H*‐48(*I*)^#^), 6.13 (s, 1H, Ar*H*‐48(*I*)^◊^), 4.32 (t, 4H, C*H*_2_‐62, *J* = 6.1 Hz), 4.28–4.20 (m, 6H, C*H*_2_‐45a(II, III, IV)), 4.23 (d, 8H, C*H*_2_‐50a, *J* = 16.2 Hz), 4.20 (1H, C*H*_2_‐45a(I)^#^), 4.12 (1H, C*H*_2_‐45a(I)^◊^), 4.08–4.00 (m, 6H, C*H*_2_‐45b(II, III, IV)), 3.96 (1H, C*H*_2_‐45b(I)^#^), 3.88 (1H, C*H*_2_‐45b(I)^◊^), 3.80–3.67 (m, 8H, C*H*_2_‐50b, *J* = 15.4 Hz), 3.55–3.42 (m, 6H, C*H*_2_‐46a(II, III, IV)), 3.47 (1H, C*H*_2_‐46a(I)^#^), 3.39–3.28 (m, 6H, C*H*_2_‐46b(II, III, IV)), 3.37 (1H, C*H*_2_‐46a(I)^◊^), 3.33 (1H, C*H*_2_‐46b(I)^#^), 3.27 (1H, C*H*_2_‐46b(I)^◊^), 2.52–2.41 (m, 2H, C*H*_2_‐63), –2.74 (s, 2H, N*H*); ^13^C NMR (CDCl_3_, 126 MHz): ∂ 158.86 (Ar*C*‐26), 158.76 (Ar*C*‐32, 38, 44), 157.07 (*C*=O‐51), 147.84 (*C*‐57), 146.62 (Ar*C*‐47), 135.78 (Ar*C*‐28, 34, 40), 133.21 (Ar*C*‐22), 131.90 (Ar*C*‐27, 33, 39), 132.20 (Ar*C*‐21), 129.85 (Ar*C*‐49), 129.63 (Ar*C*‐30, 36, 42), 128.53 (Ar*C*‐55, 56), 128.20 (Ar*C*‐54), 127.13 (Ar*C*‐24), 122.10 (Ar*C*‐23), 120.07 (*C*H‐58), 119.83 (Ar*C*‐29, 35, 41), 115.30 (Ar*C*‐6, 11, 16) 115.27 (Ar*C*‐48(II,III,IV)), 115.01 (Ar*C*‐48(I)), 114.50 (Ar*C*‐1), 112.20 (Ar*C*‐25), 111.92 (Ar*C*‐31, 37, 43), 84.78 (*C*‐52), 67.44 (*C*H_2_‐46(II, III, IV)), 67.20 (*C*H_2_‐46(I)^#^), 67.13 (*C*H_2_‐46(I)^◊^), 66.90 (*C*H_2_‐45(II, III, IV)), 66.87 (*C*H_2_‐45(I)^#^), 66.78 (*C*H_2_‐45(I)^◊^), 46.65 (*C*H_2_‐62), 44.43 (*C*H_2_‐50), 30.54 (*C*H_2_‐63); ^15^N{^1^H}‐NMR^†^ (CDCl_3_, 51 MHz): ∂ 346.55 (Triazole‐*N*‐61*), 346.44 (Triazole‐*N*‐61*), 244.89 (Triazole‐*N*‐59*), 244.77 (Triazole‐*N*‐59*); UV/Vis (CHCl_3_/CH_3_CN 1:1, v/v) λ/nm (log (ε/M^–1^ cm^–1^)) 418 (5.81), 514 (4.50), 546 (4.00), 590 (4.00), 6.43 (3.67); Accurate mass: *m/z* 2867.01780 (M+2H)^+^; calcd. for C_175_H_136_N_20_^15^N_2_O_20_: 2867.02419. ^◊^/^#^Peaks belonging to the two diastereoisomers. *Peaks belonging to the two different diastereoisomers that could not be separated. ^†^50 % of the indicated N atoms are ^15^N‐labeled.

**Double porphyrin cage compound H_4_C_5_DC**. This compound was synthesized as described for **H_4_C_3_DC**, using **Zn_2_C_5_DC** (45.53 mg, 15.1 µmol), CHCl_3_ (75 mL) and aq. HCl (6 m, 200 mL) Compound **H_4_C_5_DC** was obtained as a purple solid (35.66 mg, 12.3 µmol, 81 %).

^1^H‐NMR (CDCl_3_, 500 MHz): ∂ 8.80–8.76 (m, 4H, β‐pyrrole‐*H*), 8.76–8.70 (m, 4H, β‐pyrrole‐*H*), 8.69–8.60 (m, 8H, β‐pyrrole‐*H*), 8.36 (t, 2H, Ar*H*‐22, *J* = 2.0 Hz), 8.33–8.28 (m, 2H, Ar*H*‐24), 8.06–7.98 (m, 6H, Ar*H*‐28, 34, 40), 7.77–7.61 (m, 6H, Ar*H*‐30, 36, 42), 7.65 (2H, Triazole‐*H*‐58), 7.37–7.23 (m, 12H, Ar*H*‐29, 31, 35, 37, 41, 43), 7.28 (1H, Ar*H*‐25^#^), 7.25 (1H, Ar*H*‐25^◊^), 6.98 –6.91 (m, 12H, Ar*H*‐55, 56), 6.84–6.78 (m, 8H, Ar*H*‐54), 6.21–6.17 (m, 6H, Ar*H*‐48(II,III,IV)), 6.16 (s, 1H, Ar*H*‐48(I)^#^), 6.14 (s, 1H, Ar*H*‐48(I)^◊^), 4.30 (t, 4H, C*H*_2_‐62, *J* = 6.9 Hz), 4.27–4.15 (m, 6H, C*H*_2_‐45a(II, III, IV)), 4.23 (d, 8H, C*H*_2_‐50a, *J* = 16.0 Hz), 4.20 (1H, C*H*_2_‐45a(I)^#^), 4.18 (1H, C*H*_2_‐45a(I)^◊^), 4.08–3.93 (m, 6H, C*H*_2_‐45b(II, III, IV)), 4.00 (1H, C*H*_2_‐45b(I)^#^), 3.95 (1H, C*H*_2_‐45b(I)^◊^), 3.74 (d, 8H, C*H*_2_‐50b, *J* = 15.7 Hz), 3.53–3.41 (m, 6H, C*H*_2_‐46a(II, III, IV)), 3.47 (1H, C*H*_2_‐46a(I)^#^), 3.37–3.27 (m, 6H, C*H*_2_‐46b(II, III, IV)), 3.44 (1H, C*H*_2_‐46a(I)^◊^), 3.32 (2H, C*H*_2_‐46b(I)*), 1.94–1.87 (m, 4H, C*H*_2_‐63), 1.38–1.33 (m, 2H, C*H*_2_‐64), –2.74 (s, 2H, N*H*); ^13^C NMR (CDCl_3_, 126 MHz): ∂ 158.75 (Ar*C*‐26), 158.72 (Ar*C*‐32, 38, 44), 156.90 (*C*=O‐51), 147.70 (*C*‐57), 146.61 (Ar*C*‐47), 135.80 (Ar*C*‐28, 34, 40), 133.31 (Ar*C*‐22), 131.90 (Ar*C*‐27, 33, 39), 131.33 (Ar*C*‐21), 129.81 (Ar*C*‐49), 129.51 (Ar*C*‐30, 36, 42), 128.51 (Ar*C*‐55, 56), 128.12 (Ar*C*‐54), 126.80 (Ar*C*‐24), 122.30 (Ar*C*‐23), 119.79 (Ar*C*‐29, 35, 41), 119.14 (*C*H‐58), 115.31 (Ar*C*‐48(II,III,IV)), 115.30 (Ar*C*‐6, 11, 16), 114.99 (Ar*C*‐48(I)), 114.40 (Ar*C*‐1), 112.04 (Ar*C*‐25), 111.95 (Ar*C*‐31, 37, 43), 84.77 (*C*‐52), 67.45 (*C*H_2_‐46(II, III, IV)), 67.21 (*C*H_2_‐46(I)*), 66.87 (*C*H_2_‐45), 49.90 (*C*H_2_‐62), 44.44 (*C*H_2_‐50), 29.62 (*C*H_2_‐63), 23.41(*C*H_2_‐64); ^15^N{^1^H}‐NMR^†^ (CDCl_3_, 51 MHz): ∂ 344.66 (Triazole‐*N*‐61*), 344.61 (Triazole‐*N*‐61*), 247.82 (Triazole‐*N*‐59*); UV/Vis (CHCl_3_/CH_3_CN 1:1, v/v) λ/nm (log (ε/M^–1^ cm^–1^)) 420 (5.81), 515 (4.45), 5,46 (4.12), 590 (4.15), 642 (3.78); Accurate mass: *m/z* 2895.04174 (M+2H)^+^; calcd. for C_177_H_140_N_20_^15^N_2_O_20_: 2895.05549. ^◊^/^#^Peaks belonging to the two diastereoisomers. *Peaks belonging to the two different diastereoisomers that could not be separated. ^†^50 % of the indicated N atoms are ^15^N‐labeled.

**Double porphyrin cage compound H_4_C_11_DC**. This compound was synthesized as described for **H_4_C_3_DC**, using **Zn_2_C_11_DC** (39.03 mg, 12.6 µmol), CHCl_3_ (50 mL) and aq. HCl (6 m, 200 mL). Compound **H_4_C_11_DC** was obtained as a purple solid (34.59 mg, 11.6 µmol, 92 %).

^1^H‐NMR (CDCl_3_, 500 MHz): ∂ 8.80–8.77 (m, 4H, β‐pyrrole‐*H*), 8.76–8.72 (m, 4H, β‐pyrrole‐*H*), 8.71–8.61 (m, 8H, β‐pyrrole‐*H*), 8.35–8.33 (m, 2H, Ar*H*‐22), 8.33–8.31 (m, 2H, Ar*H*‐24), 8.07–8.01 (m, 6H, Ar*H*‐28, 34, 40), 7.75–7.66 (m, 6H, Ar*H*‐30, 36, 42), 7.56–7.52 (m, 2H, Triazole‐*H*‐58), 7.38–7.273 (m, 12H, Ar*H*‐29, 31, 35, 37, 41, 43), 7.35 (2H, Ar*H*‐25), 6.99 –6.91 (m, 12H, Ar*H*‐55, 56), 6.84–6.78 (m, 8H, Ar*H*‐54), 6.21–6.15 (m, 8H, Ar*H*‐48), 4.29 (2H, C*H*_2_‐45a(I)), 4.23 (6H, C*H*_2_‐45a(II, III, IV)), 4.23 (d, 8H, C*H*_2_‐50a, *J* = 15.6 Hz), 4.16 (t, 4H, C*H*_2_‐62, *J* = 7.2 Hz), 4.12–3.97 (m, 6H, C*H*_2_‐45b(II, III, IV)), 4.09 (2H, C*H*_2_‐45b(I)), 3.74 (d, 8H, C*H*_2_‐50b, *J* = 15.8 Hz), 3.57–3.44 (m, 6H, C*H*_2_‐46a(II, III, IV)), 3.55 (2H, C*H*_2_‐46a(I)), 3.39 (2H, C*H*_2_‐46b(I)), 3.36–3.28 (m, 6H, C*H*_2_‐46b(II, III, IV)), 1.73 (4H, C*H*_2_‐63), 1.19–1.08 (m, 14H, C*H*_2_‐64, 65, 66, 67), –2.73 (s, 2H, N*H*); ^13^C NMR (CDCl_3_, 126 MHz): ∂ 158.77 (Ar*C*‐32, 38, 44), 158.72 (Ar*C*‐26), 157.03 (*C*=O‐51), 147.48 (*C*‐57), 146.68 (Ar*C*‐47), 135.88 (Ar*C*‐28, 34, 40), 133.20 (Ar*C*‐22), 131.99 (Ar*C*‐21), 131.82 (Ar*C*‐27, 33, 39), 129.82 (Ar*C*‐49), 129.73 (Ar*C*‐30, 36, 42), 128.66 (Ar*C*‐55, 56), 128.25 (Ar*C*‐54), 127.20 (Ar*C*‐24), 122.54 (Ar*C*‐23), 119.96 (Ar*C*‐29, 35, 41), 119.04 (*C*H‐58), 115.47 (Ar*C*‐48(II,III,IV)), 115.46 (Ar*C*‐48(I)), 115.29 (Ar*C*‐6, 11, 16) 114.54 (Ar*C*‐1), 112.28 (Ar*C*‐25), 112.15 (Ar*C*‐31, 37, 43), 84.80 (*C*‐52), 67.62 (*C*H_2_‐46(II, III, IV)), 67.37 (*C*H_2_‐46(I)), 67.20 (*C*H_2_‐45(I)), 67.04 (*C*H_2_‐45(II, III, IV)), 50.40 (*C*H_2_‐62), 44.42 (*C*H_2_‐50), 30.34 (*C*H_2_‐63), 26.60 (*C*H_2_‐64, 65, 66, 67); ^15^N{^1^H}‐NMR^†^ (CDCl_3_, 51 MHz): ∂ 343.68 (Triazole‐*N*‐61), 249.14 (Triazole‐*N*‐59); UV/Vis (CHCl_3_/CH_3_CN 1:1, v/v) λ/nm (log (ε/M^–1^ cm^–1^)) 420 (5.90), 515 (4.58), 548 (4.08), 590 (4.08), 644 (3.78); Accurate mass: *m/z* 2979.14800 (M+2H)^+^; calcd. for C_183_H_152_N_20_^15^N_2_O_20_: 2979.14939. ^†^50 % of the indicated N atoms are ^15^N‐labeled.

## Supporting information

Supporting Information
