# Supplementary material for: Double Porphyrin Cage Compounds
Source: European J Org Chem. 2020 Nov 16;2020(45):7087–100. doi: 10.1002/ejoc.202001211 (PMC7756431; doi:10.1002/ejoc.202001211)
Supplement: Supplementary file 1 — Supporting Information [file EJOC-2020-7087-s001.pdf]

European Journal of Organic Chemistry

Supporting Information

Double Porphyrin Cage Compounds

Kathleen Stout, Theo P. J. Peters, Mathijs F. J. Mabesoone, Fabian L. L. Visschers, Eline M. Meijer, Joëlle-Rose Klop, Jeroen van den Berg, Paul B. White, Alan E. Rowan, Roeland J. M. Nolte,* Johannes A. A. W. Elemans*

Index

1. NMR spectral data of new compounds 2

2. Titration methods 29

    1.1 UV-vis titrations 29

    1.2 NMR titrations 35

1. NMR spectral data of new compounds

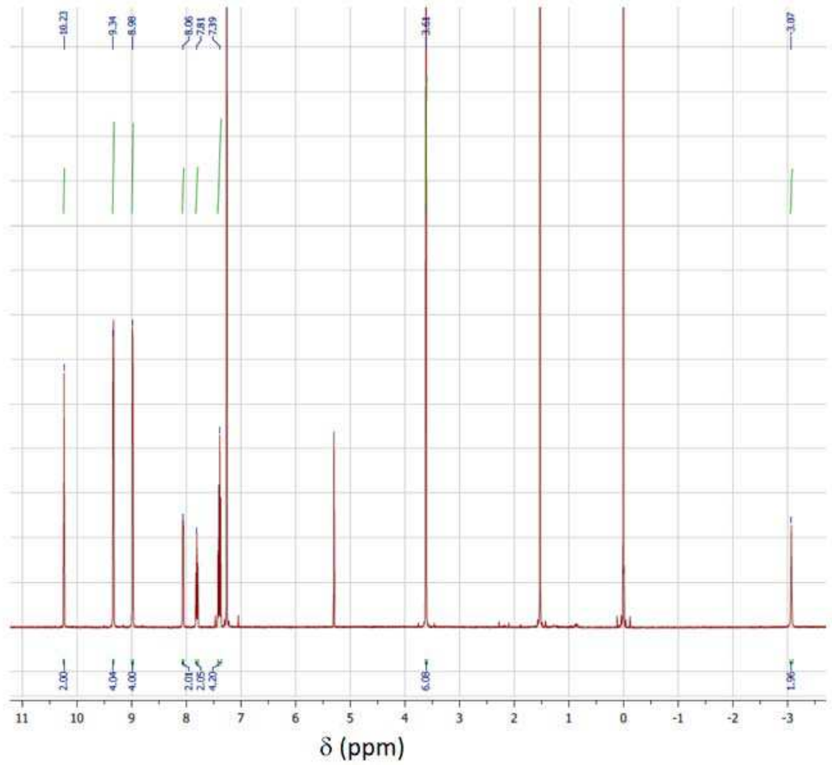

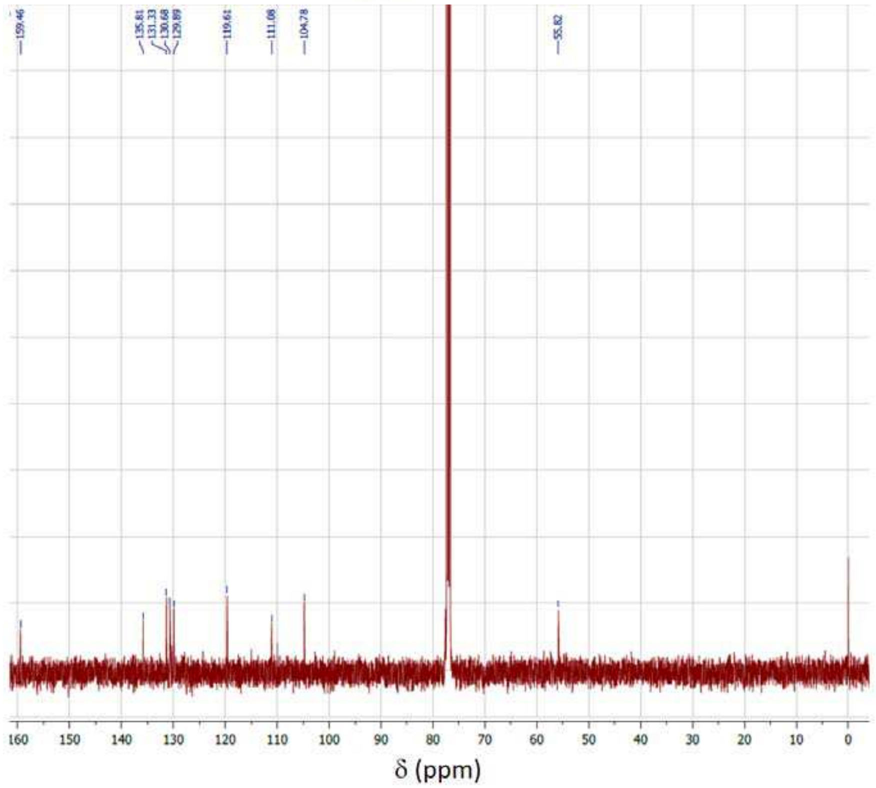

Figure S1 ^{1}H NMR (500 MHz, top) and ^{13}C NMR (126 MHz, bottom) spectra of compound **2** in $CDCl_3$.

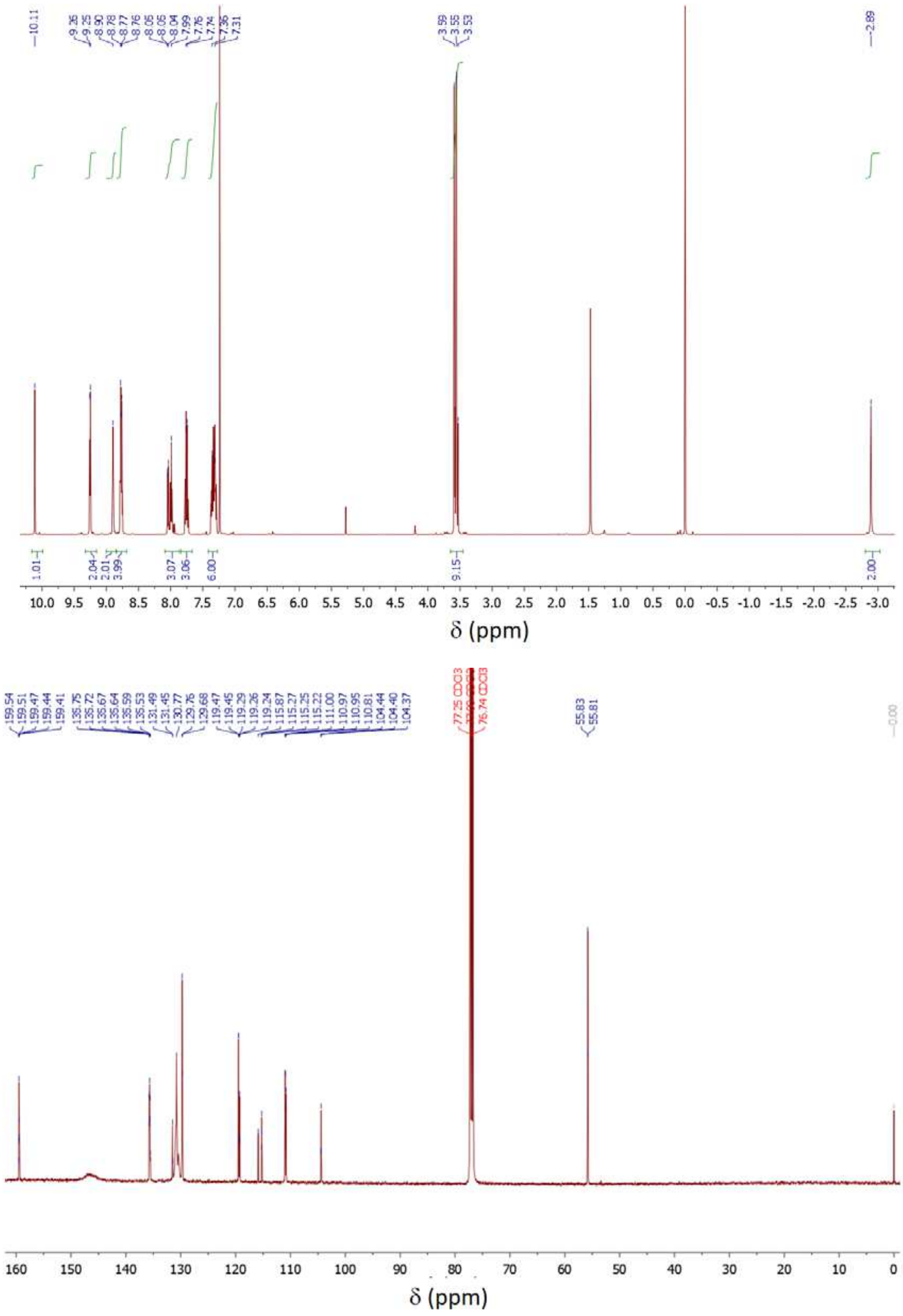

Figure S2 ^{1}H NMR (500 MHz, top) and ^{13}C NMR (126 MHz, bottom) spectra of compound **3** in $CDCl_3$.

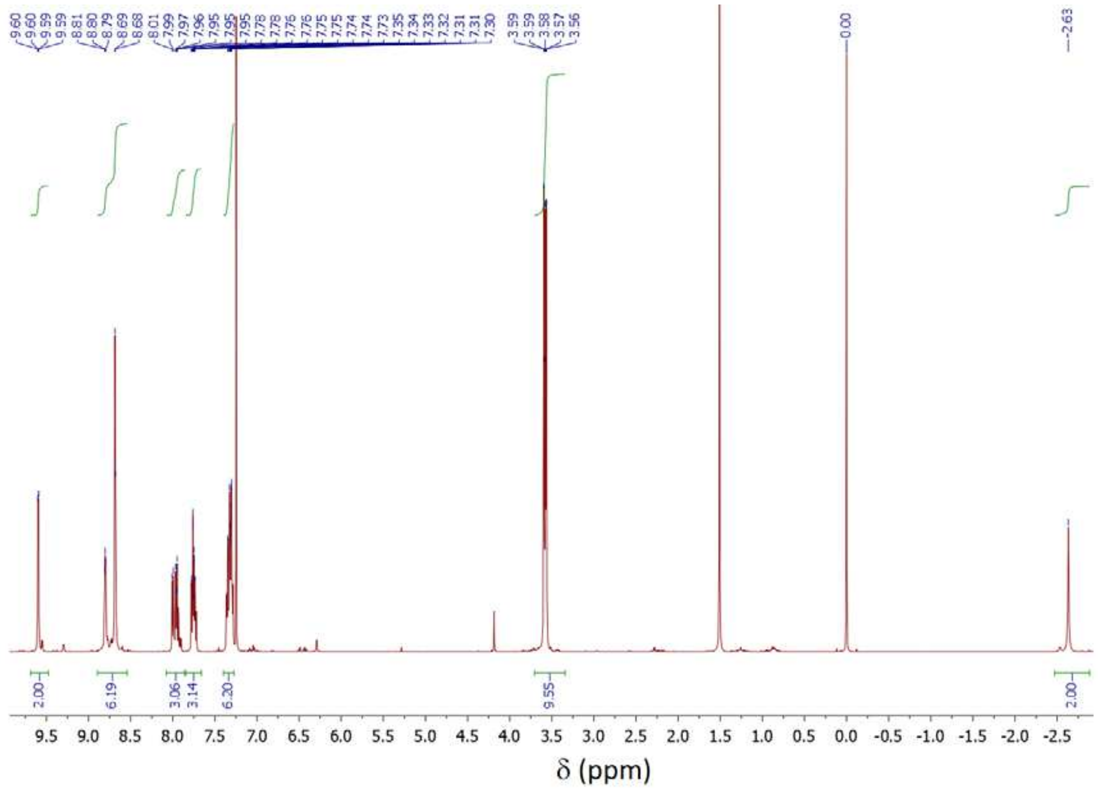

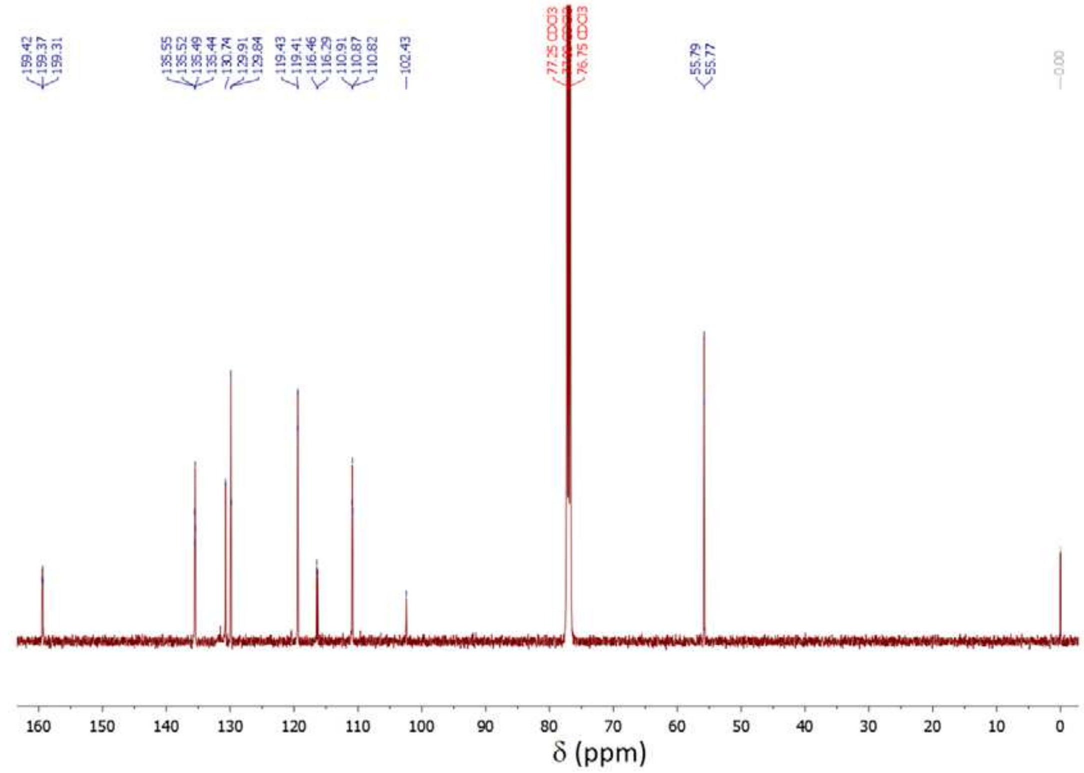

Figure S3 ^{1}H NMR (500 MHz, top) and ^{13}C NMR (126 MHz, bottom) spectra of compound **4** in $CDCl_3$.

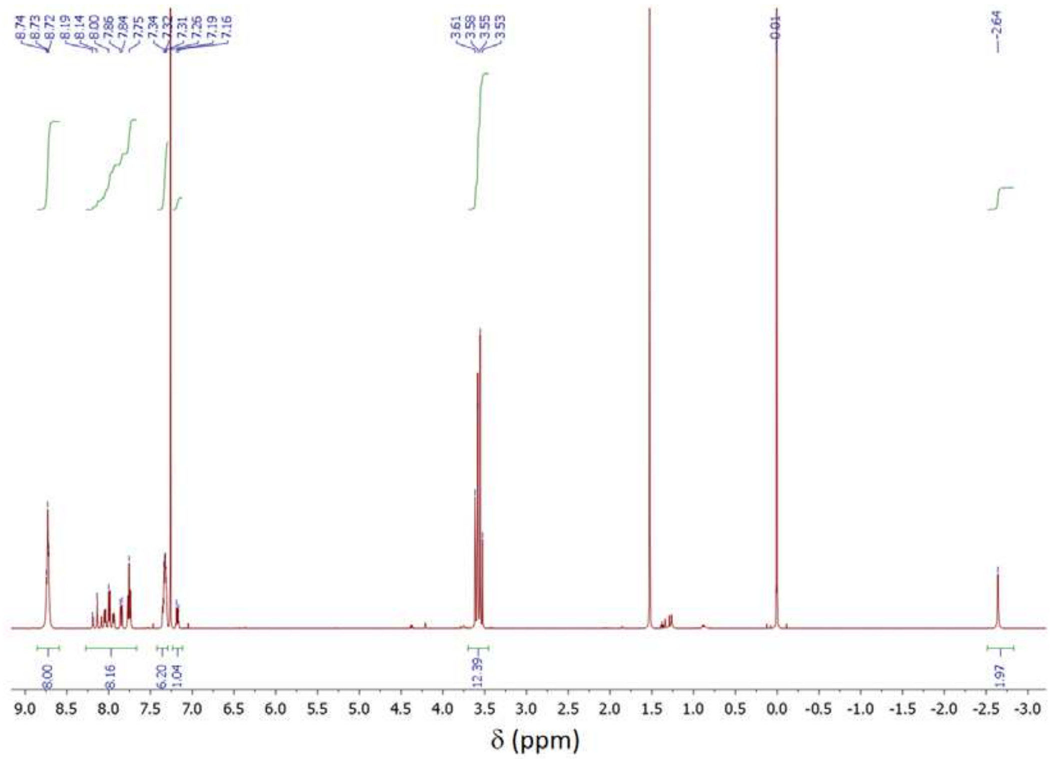

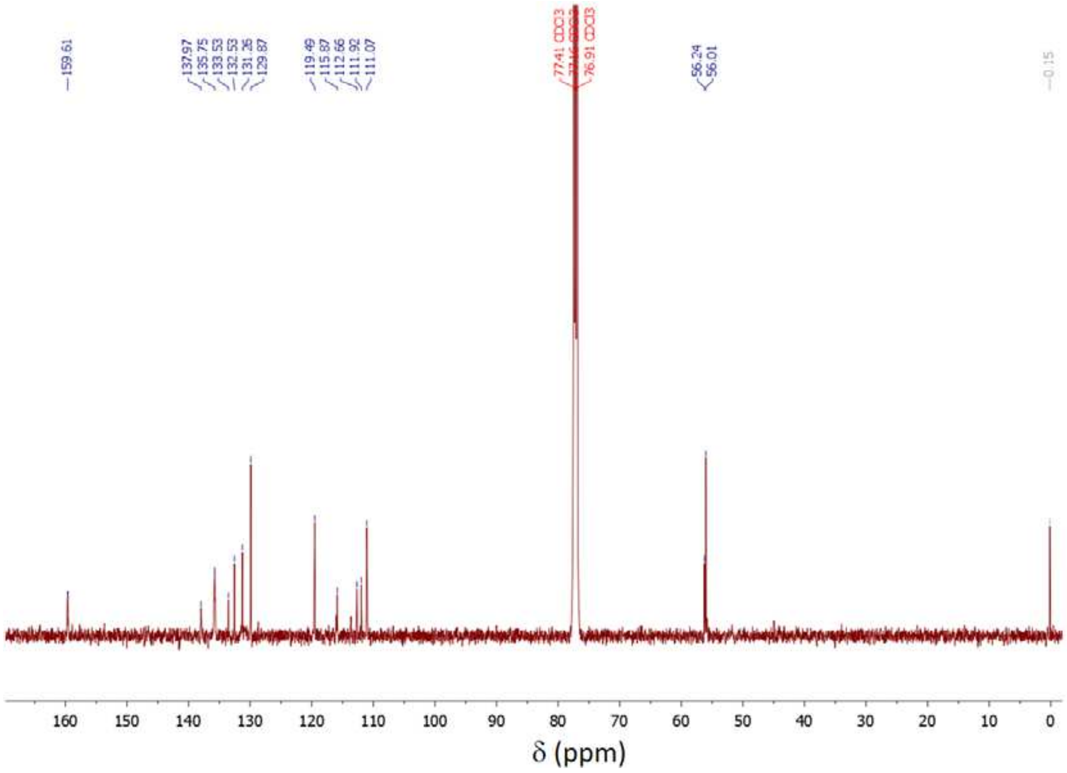

Figure S4 ^{1}H NMR (500 MHz, top) and ^{13}C NMR (126 MHz, bottom) spectra of compound **5** in $CDCl_3$.

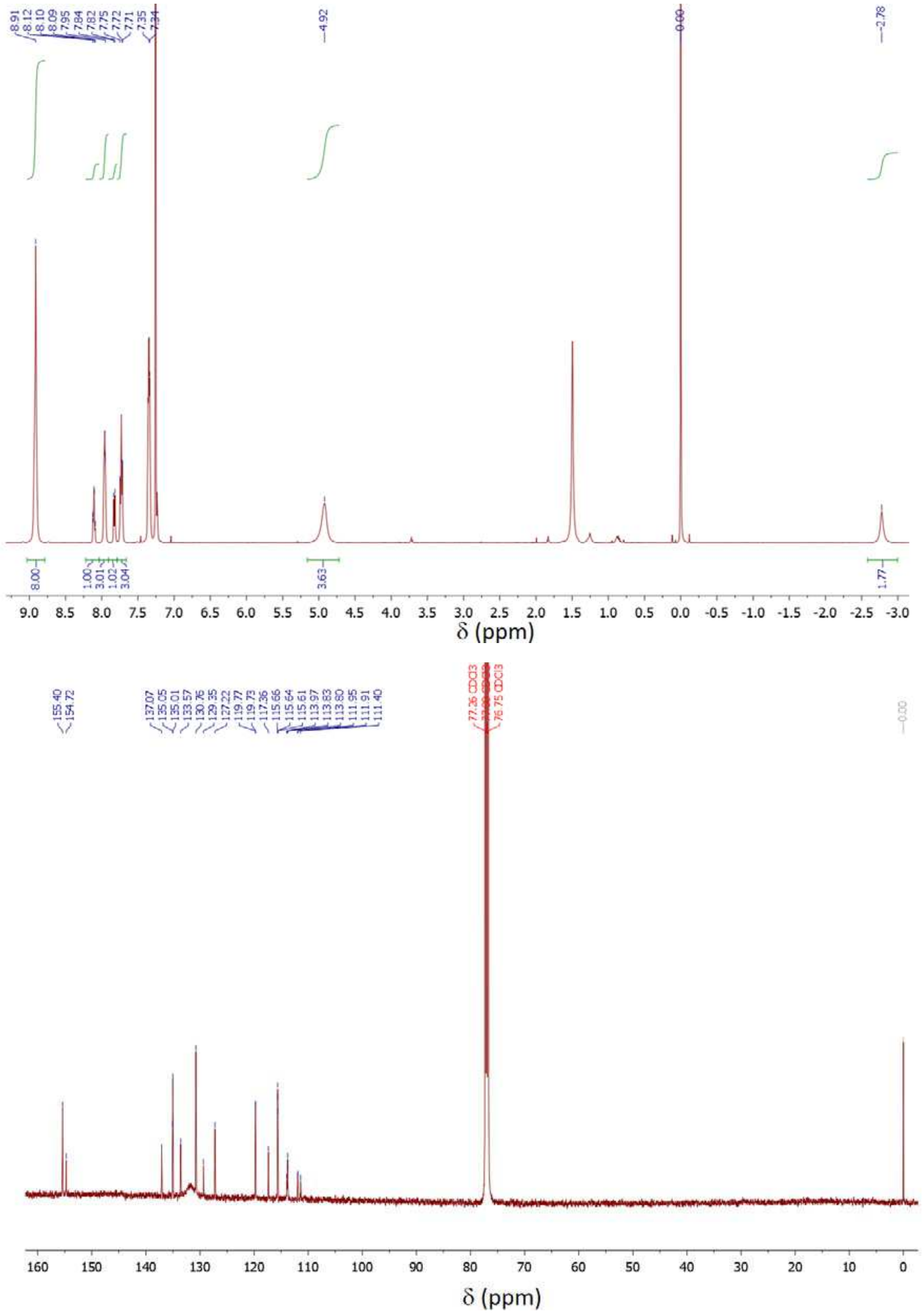

Figure S5 ^{1}H NMR (500 MHz, top) and ^{13}C NMR (126 MHz, bottom) spectra of compound **6** in $CDCl_3$.

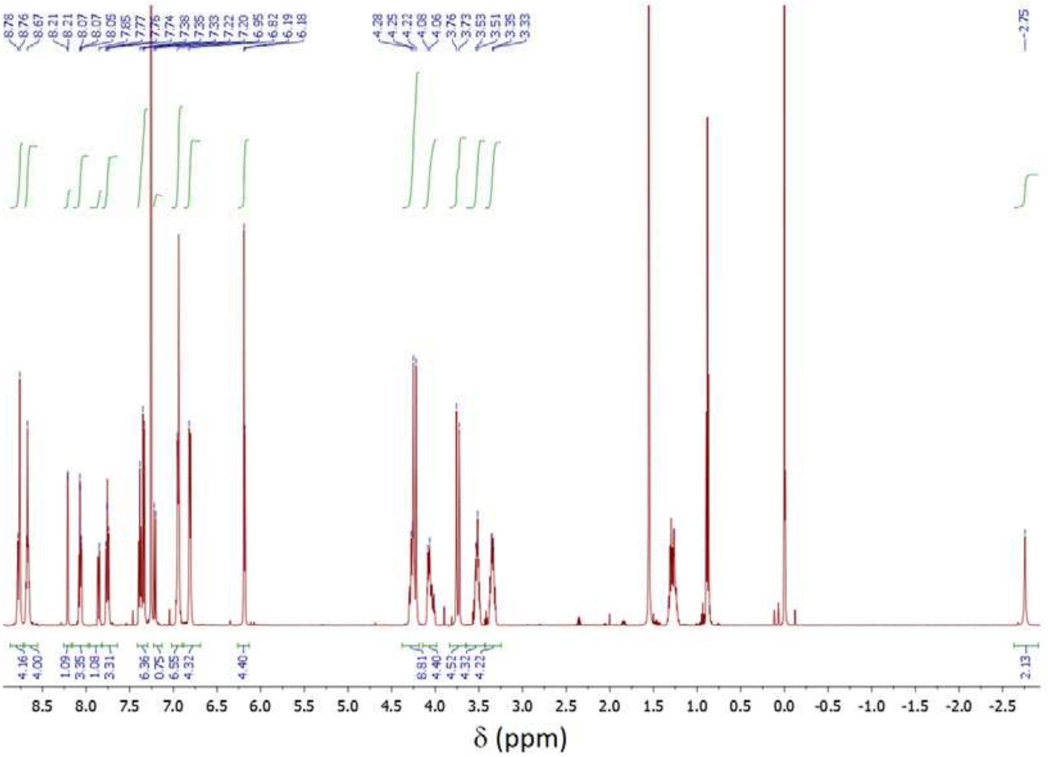

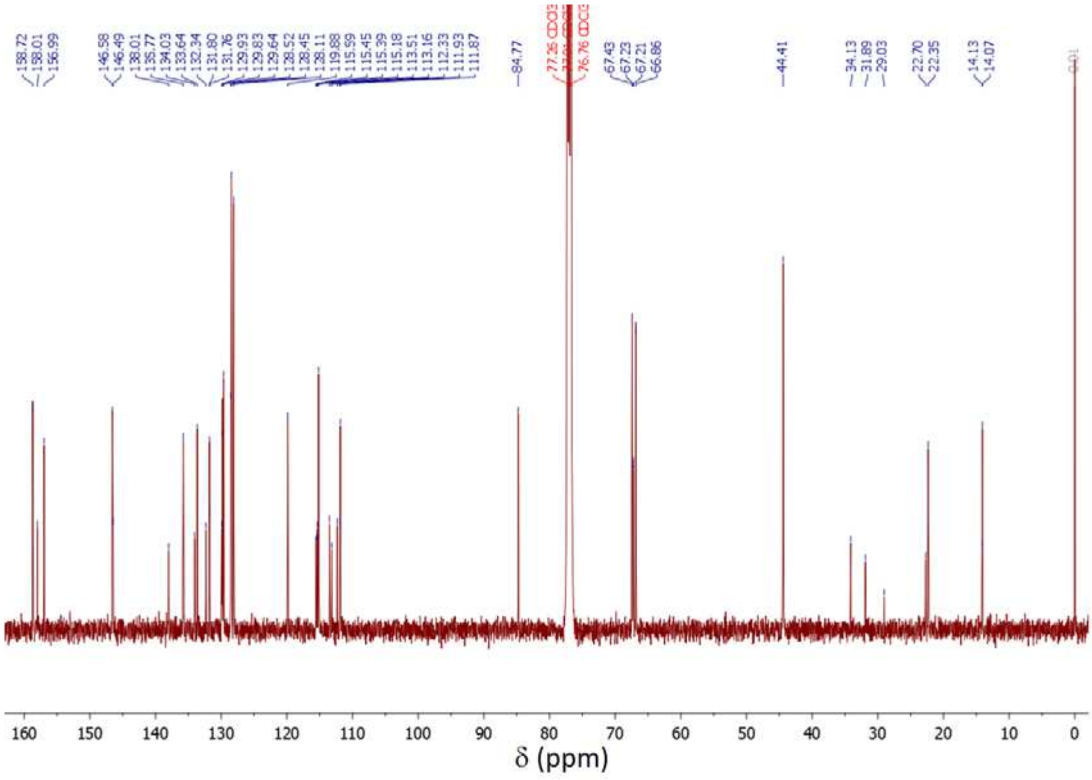

Figure S6 ^{1}H NMR (500 MHz, top) and ^{13}C NMR (126 MHz, bottom) spectra of compound **8** in $CDCl_3$.

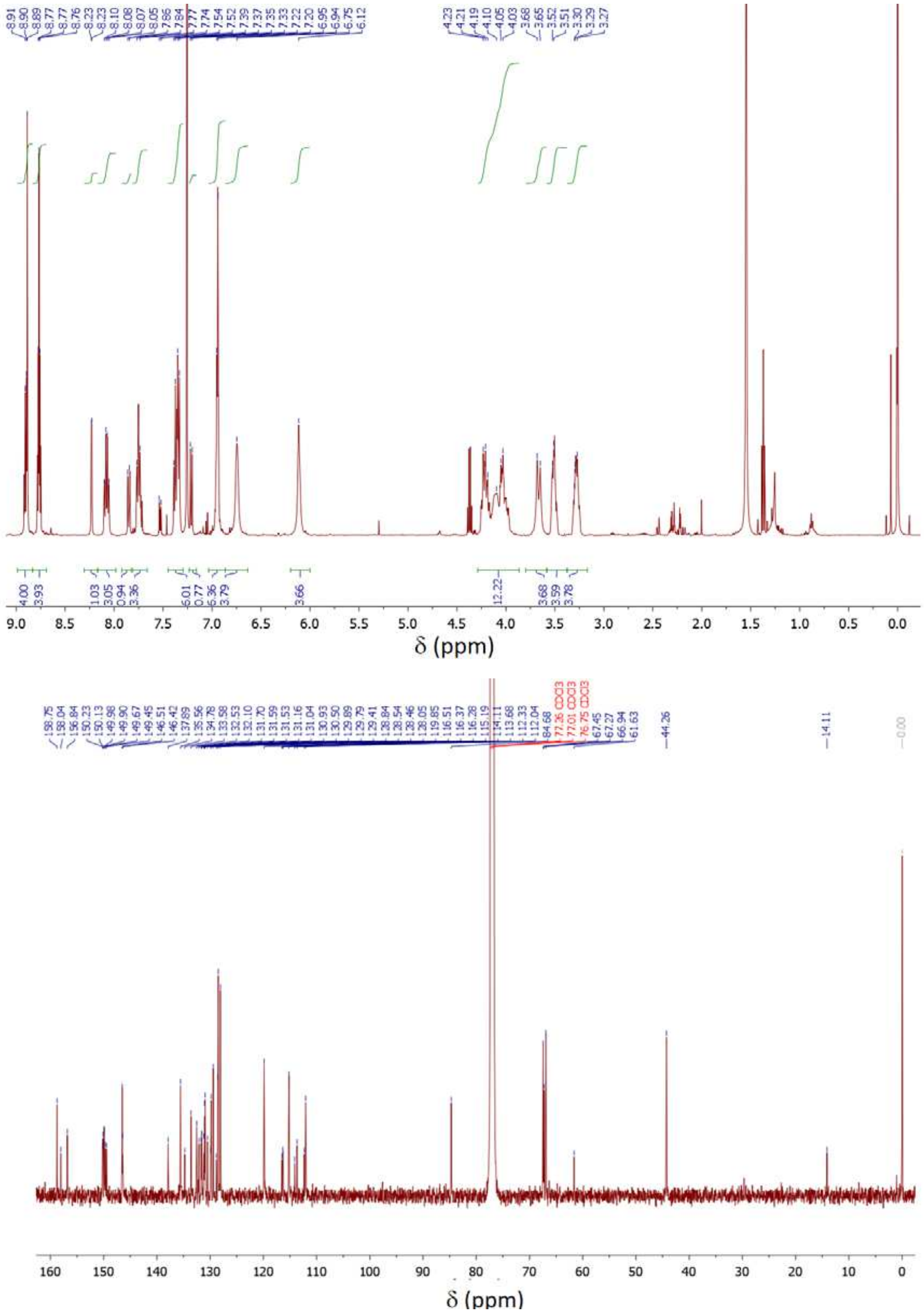

Figure S7 ^{1}H NMR (500 MHz, top) and ^{13}C NMR (126 MHz, bottom) spectra of compound **9** in $CDCl_3$.

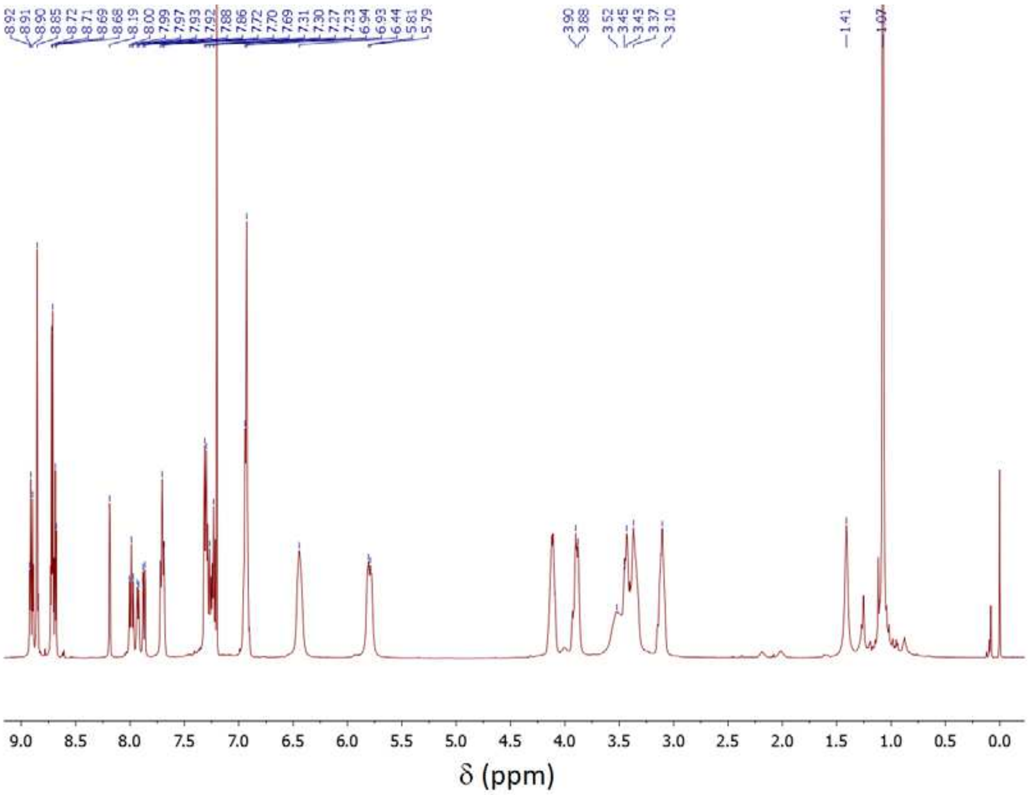
8.92
8.91
8.90
8.85
8.72
8.71
8.69
8.68
8.19
8.00
7.99
7.97
7.93
7.92
7.88
7.86
7.72
7.70
7.69
7.31
7.30
7.27
7.23
6.94
6.93
6.44
5.81
5.79
3.90
3.88
3.52
3.45
3.43
3.37
3.10
1.41
1.07
9.0
8.5
8.0
7.5
7.0
6.5
6.0
5.5
5.0
4.5
4.0
3.5
3.0
2.5
2.0
1.5
1.0
0.5
0.0
δ (ppm)

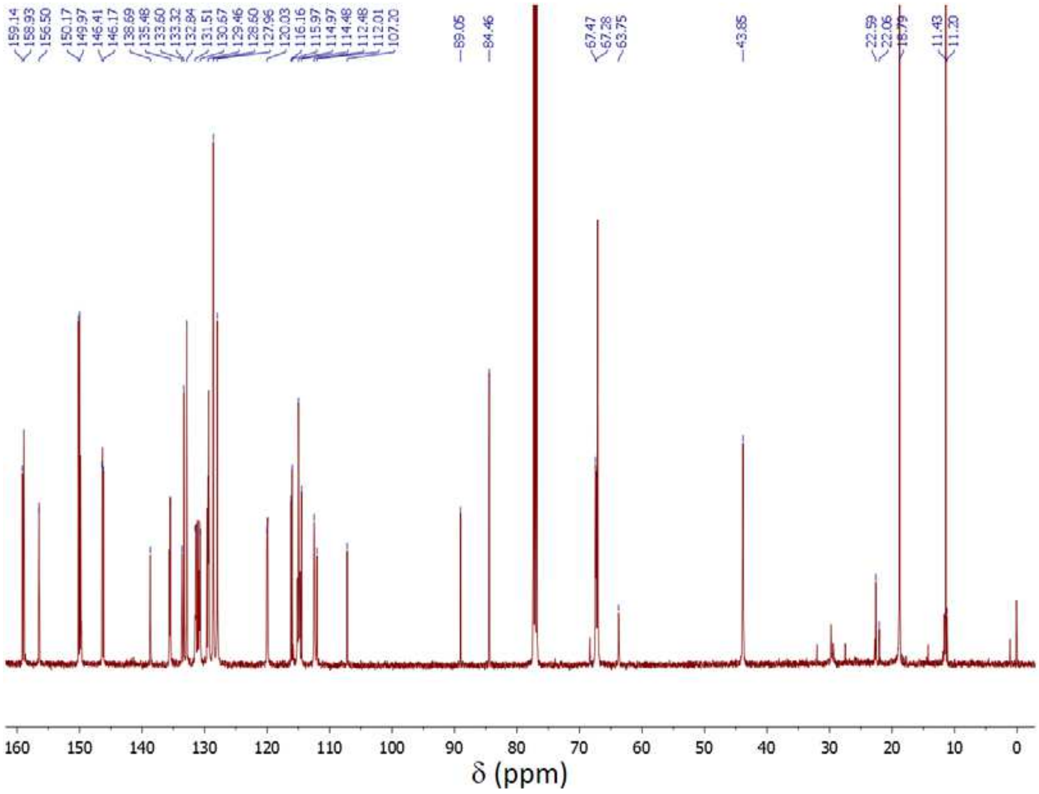
159.14
158.93
156.50
150.17
149.97
146.41
146.17
138.69
135.48
133.60
133.32
132.84
131.51
130.67
129.46
128.60
127.96
120.03
116.16
115.97
114.97
114.48
112.48
112.01
107.20
89.05
84.46
67.47
67.28
63.75
43.85
22.59
22.06
18.79
11.43
11.20
160
150
140
130
120
110
100
90
80
70
60
50
40
30
20
10
0
δ (ppm)

Figure S8 ^{1}H NMR (500 MHz, top) and ^{13}C NMR (126 MHz, bottom) spectra of compound **10** in $CDCl_3$.

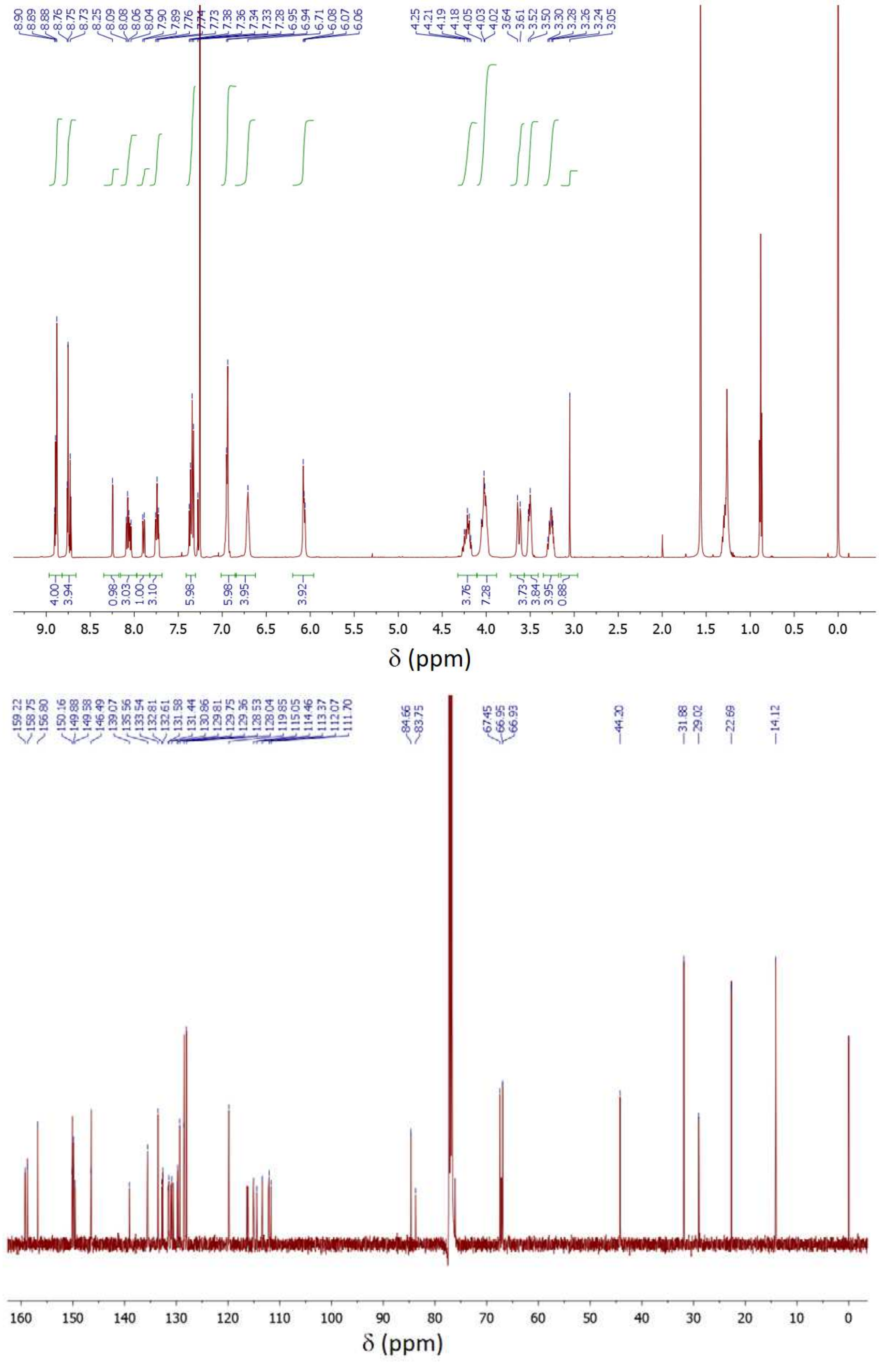

Figure S9 ^{1}H NMR (500 MHz, top) and ^{13}C NMR (126 MHz, bottom) spectra of compound **11** in $CDCl_3$.

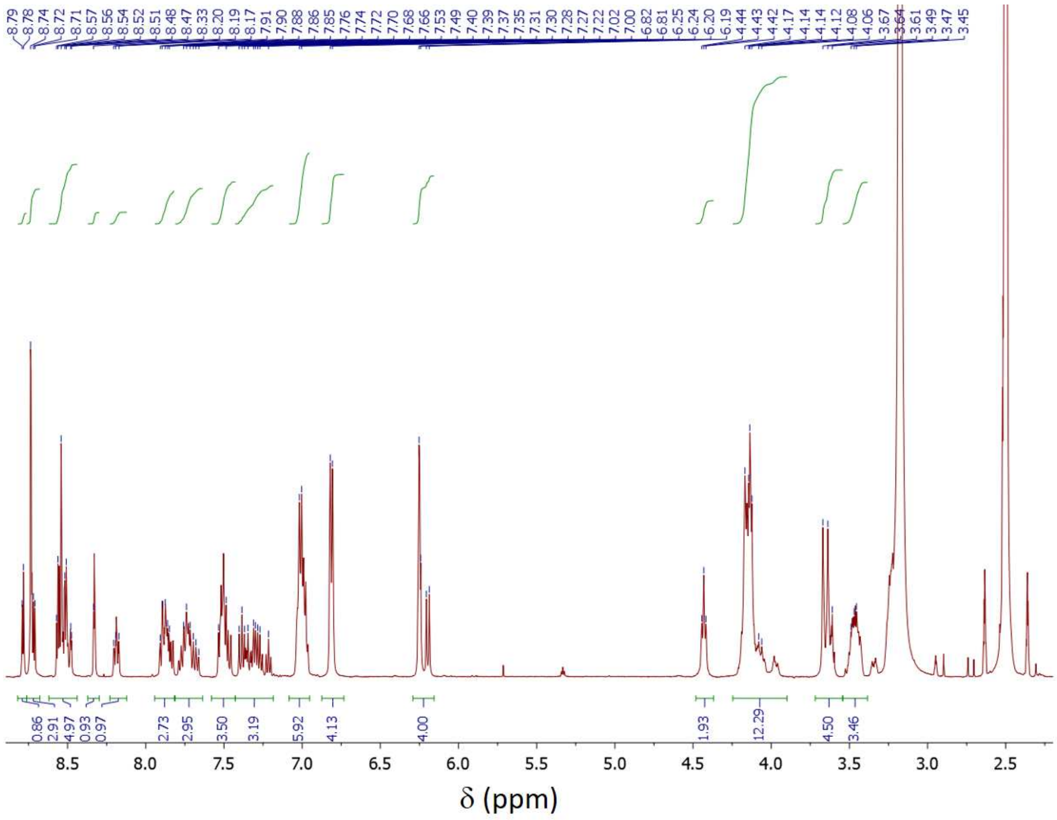

Figure S10 ^{1}H NMR spectrum of compound **Zn_2C_3DC** in DMSO-d6.

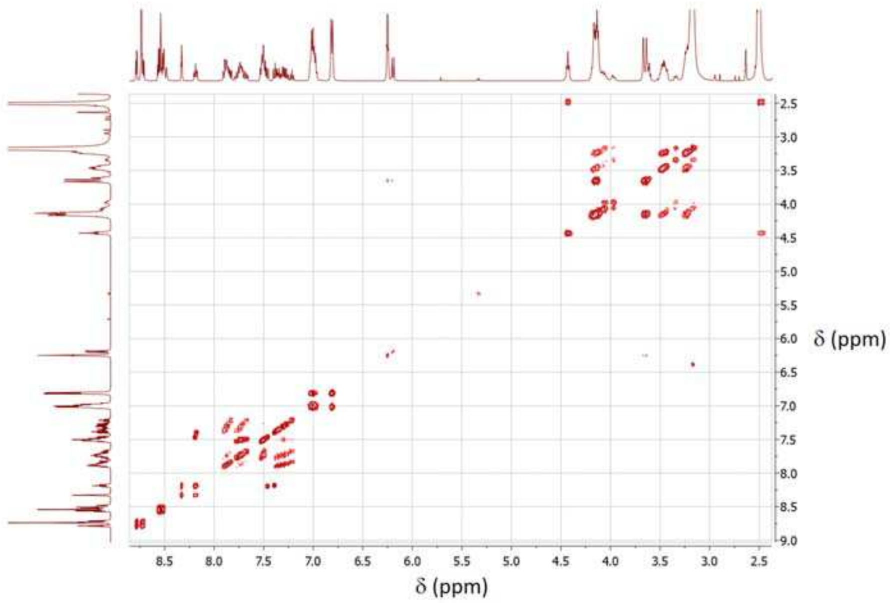

Figure S11 COSY spectrum of compound **Zn_2C_3DC** in DMSO-d6.

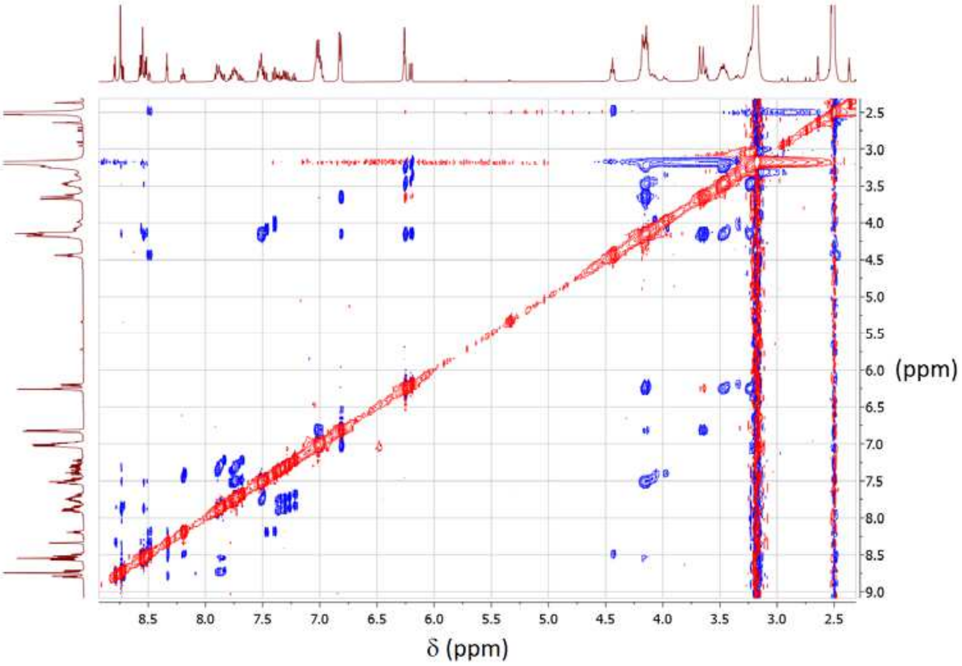

Figure S12 2D-ROESY spectrum of compound **Zn_2C_3DC** in DMSO-d6.

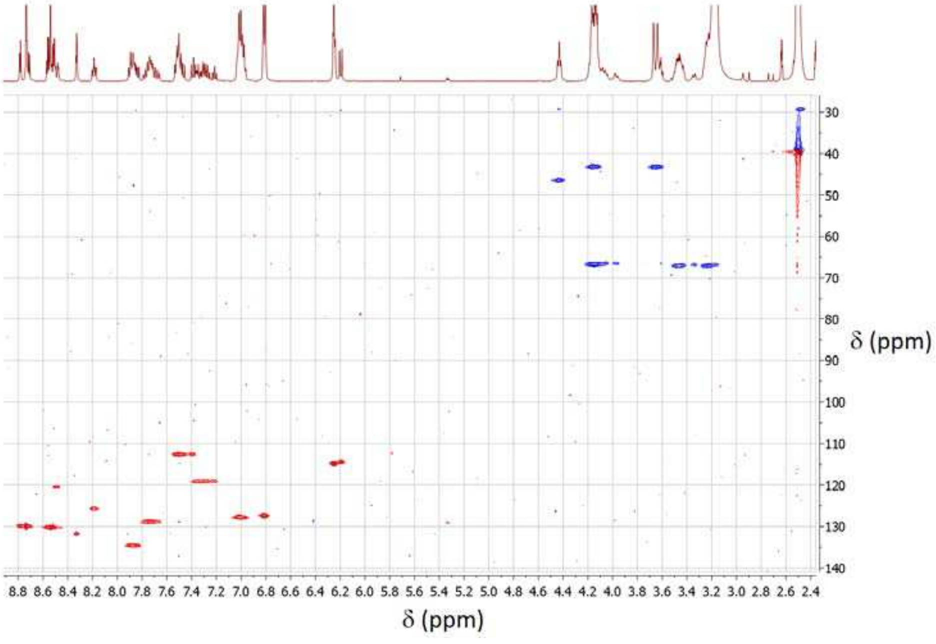

Figure S13 ^{1}H-^{13}C HSQC spectrum of compound **Zn_2C_3DC** in DMSO-d6 at 60°C.

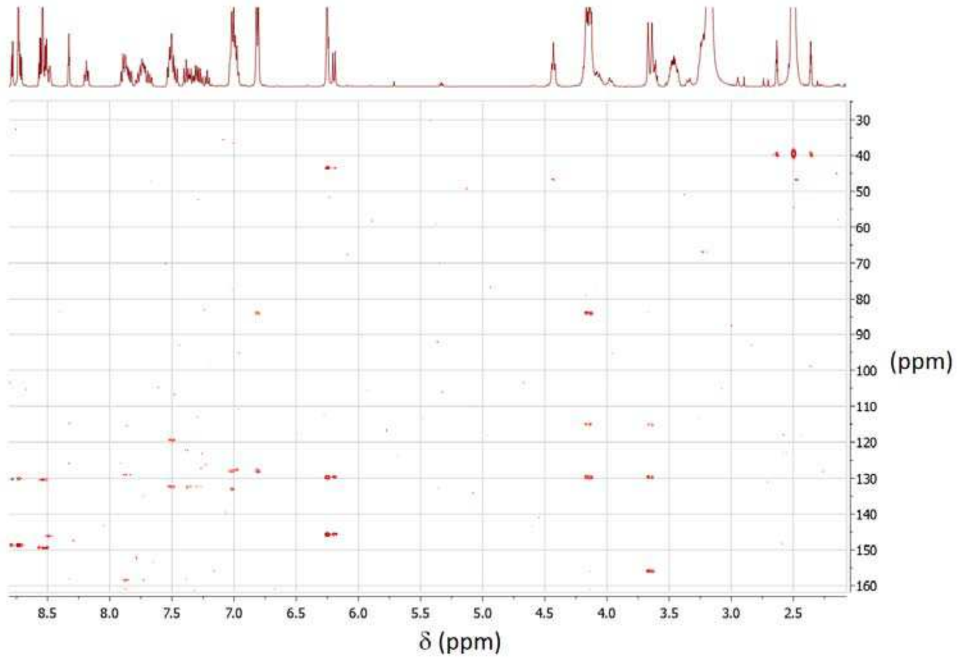

Figure S14 ^{1}H-^{13}C HMBC spectrum of compound **Zn_2C_3DC** in DMSO-d6 at 60°C.

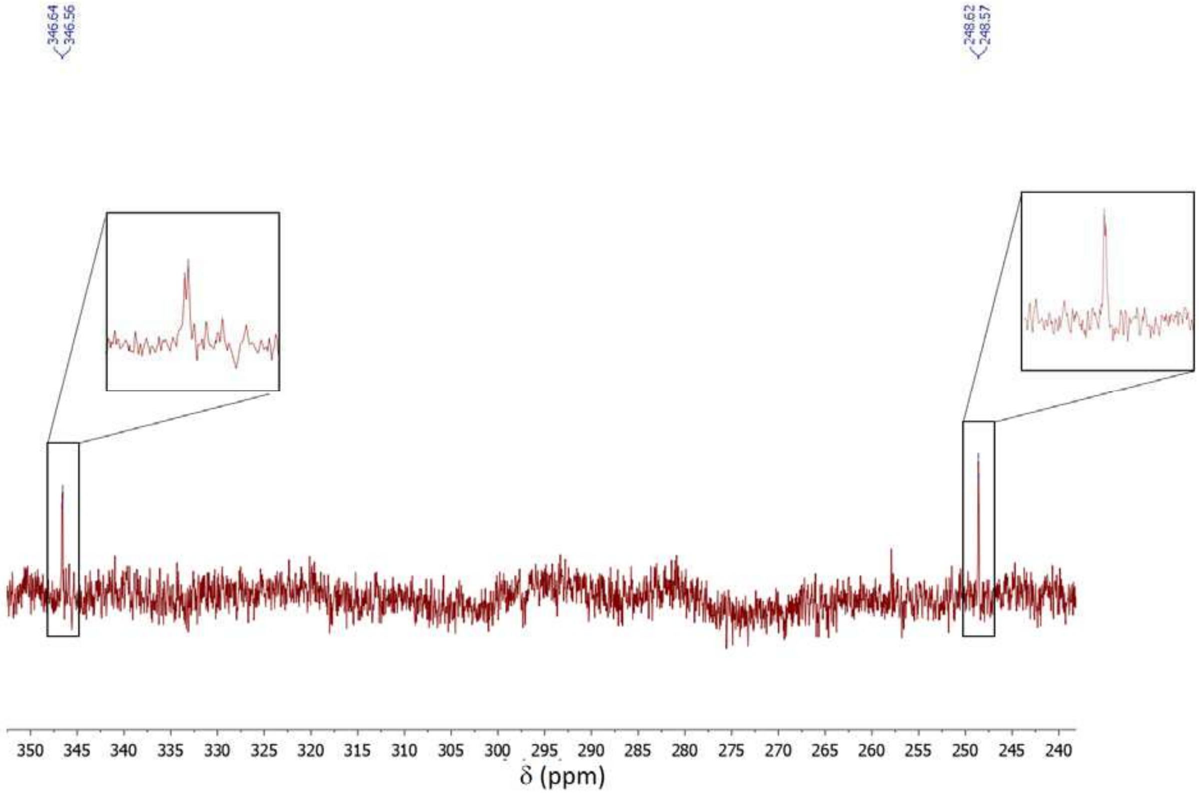

Figure S15 ^{15}N NMR spectrum of compound **Zn_2C_3DC** in DMSO-d6 at 60°C.

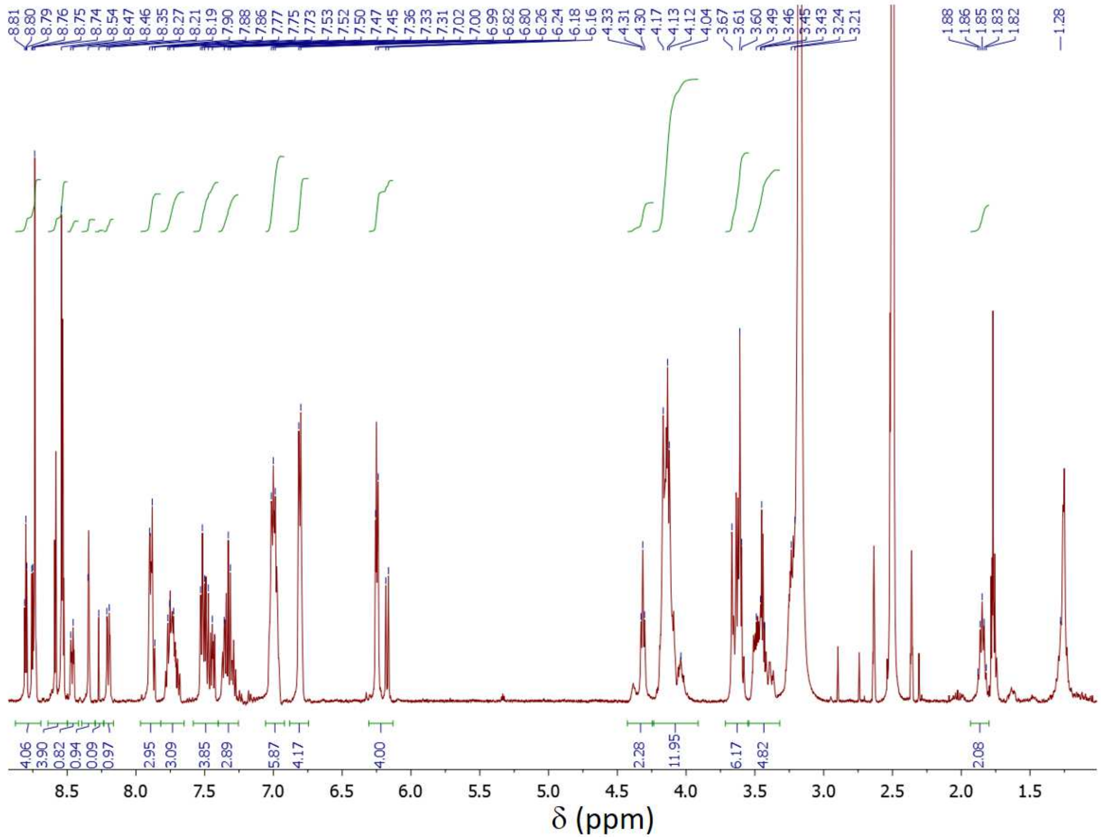

Figure S16 ^{1}H NMR spectrum of compound **Zn_2C_5DC** in DMSO-d6.

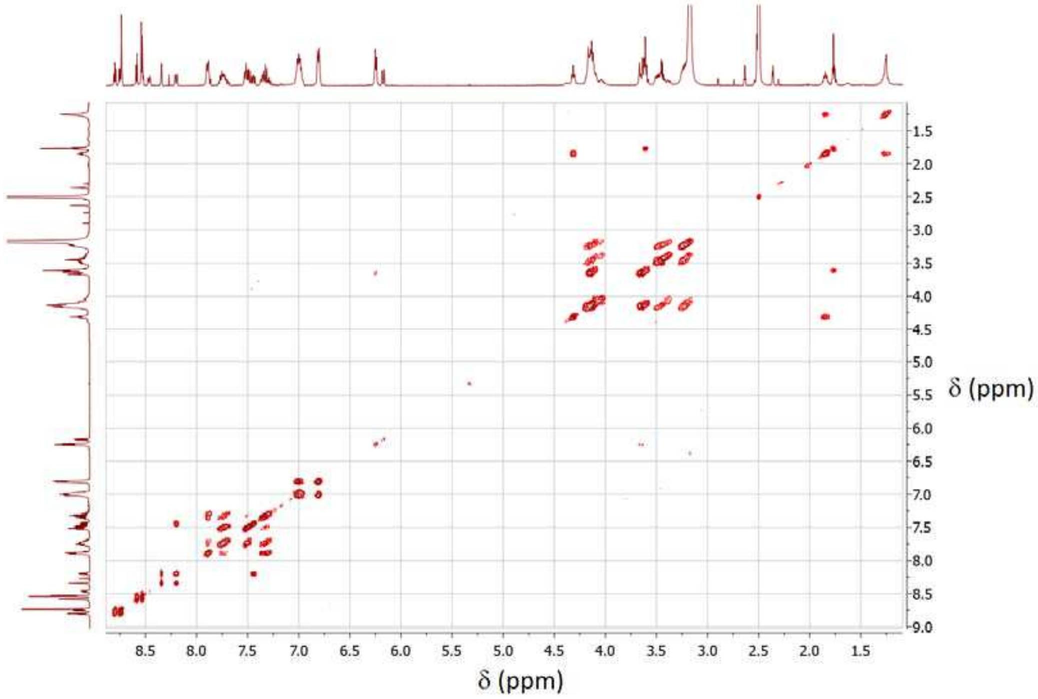

Figure S17 COSY spectrum of compound Zn_2C_5DC in DMSO-d6.

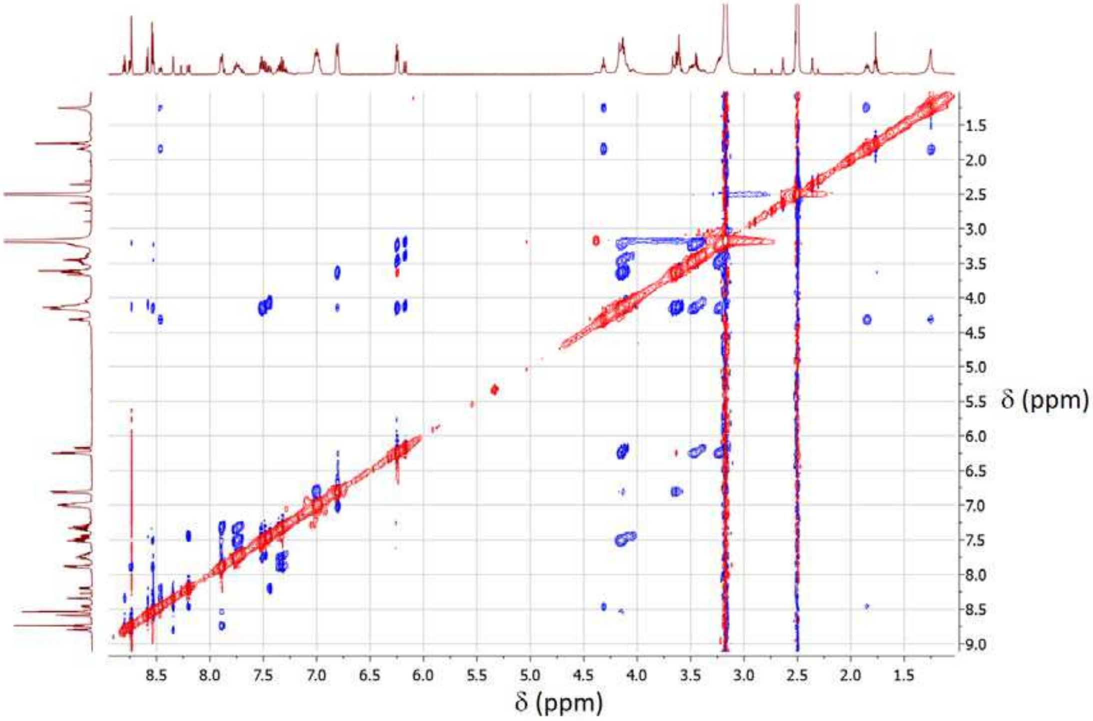

Figure S18 2D-ROESY spectrum of compound Zn_2C_5DC in DMSO-d6.

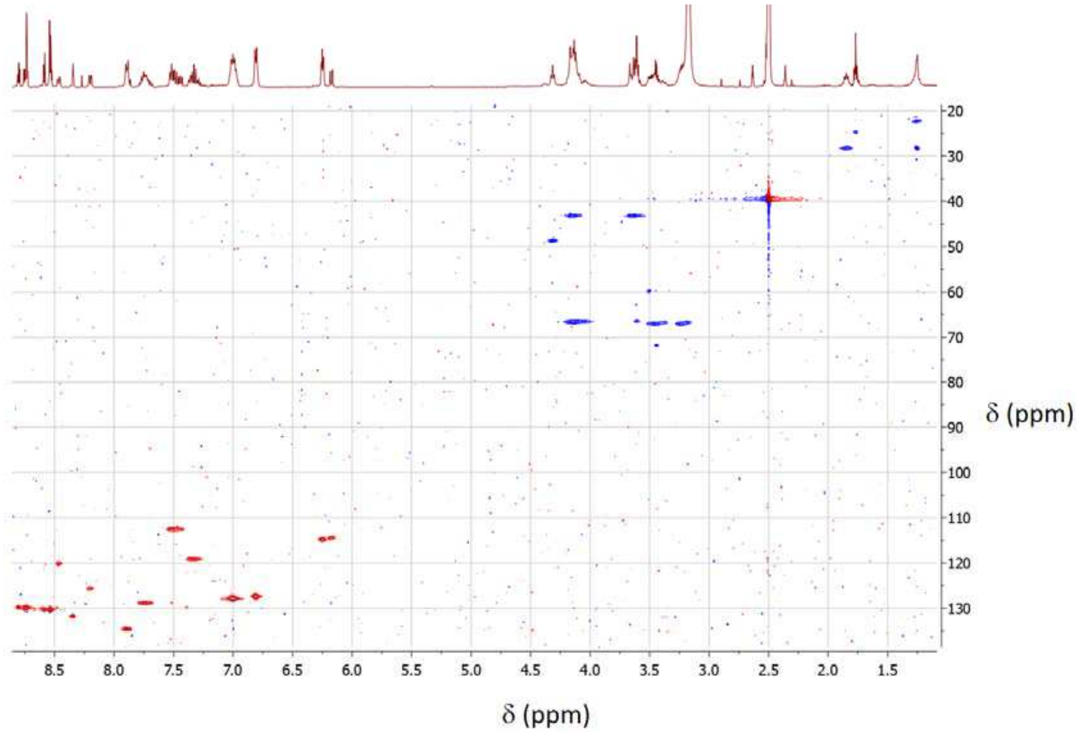

Figure S19 ^{1}H-^{13}C HSQC spectrum of compound **Zn_2C_3DC** in DMSO-d6 at 60°C.

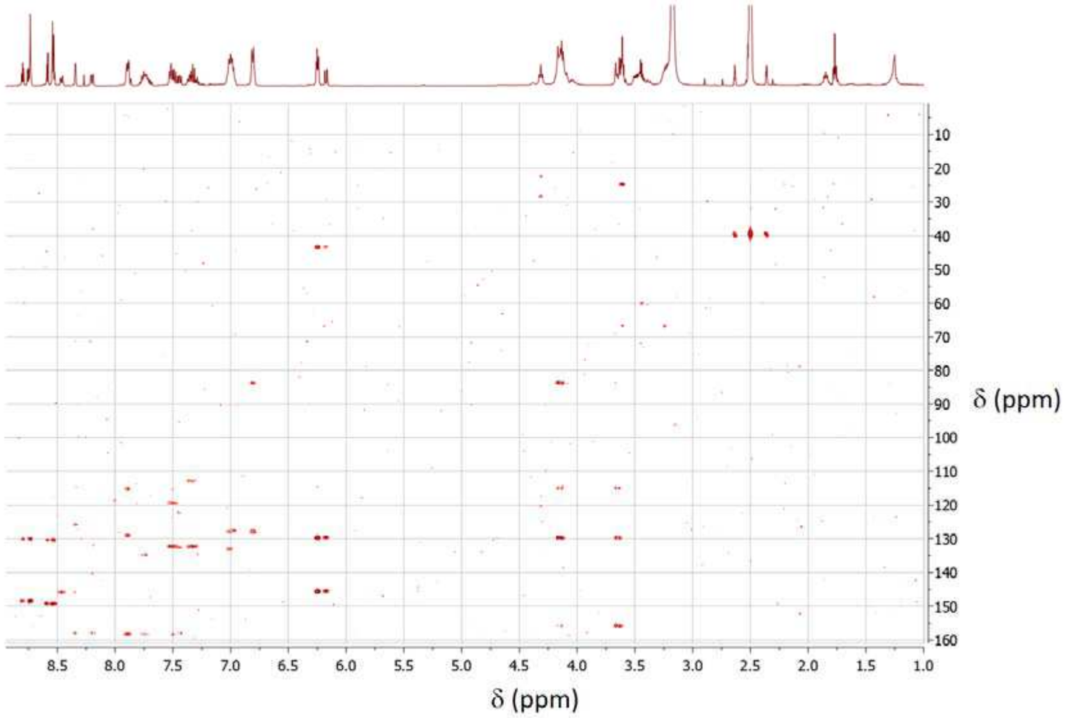

Figure S20 ^{1}H-^{13}C HMBC spectrum of compound **Zn_2C_5DC** in DMSO-d6 at 60°C.

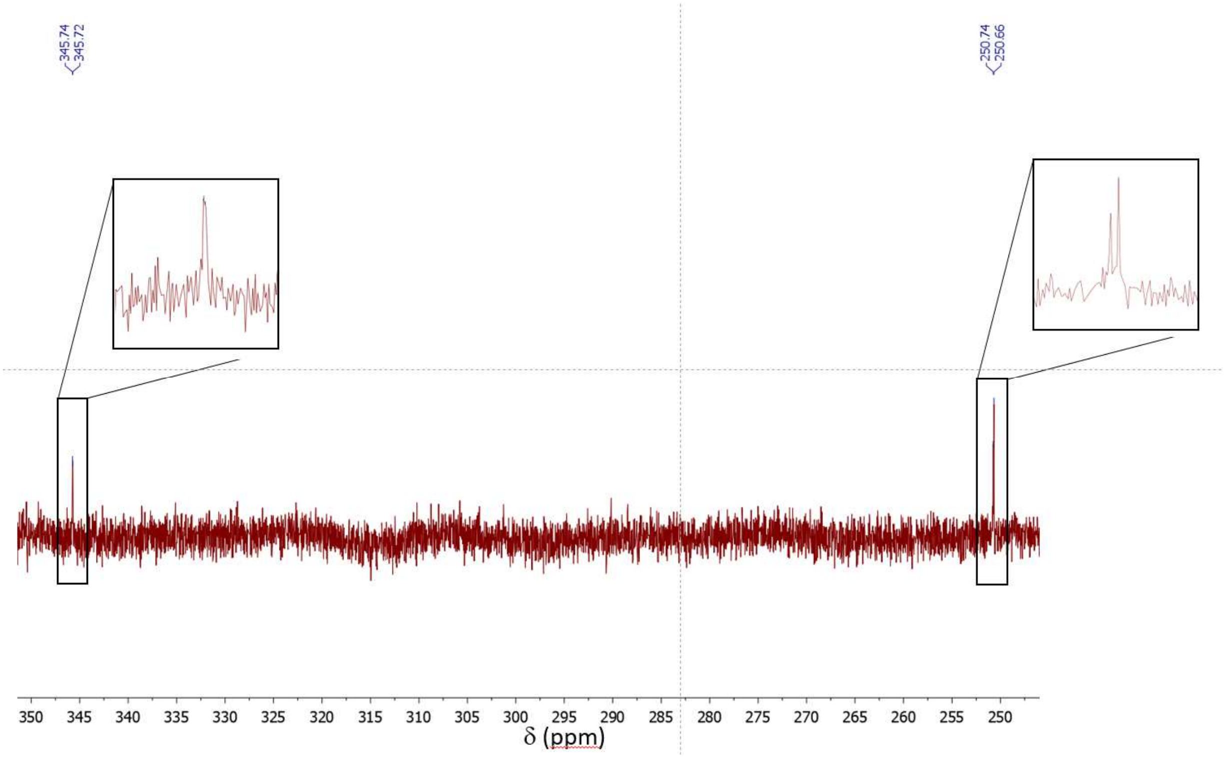

Figure S21 ^{15}N NMR spectrum of compound **Zn_2C_5DC** in DMSO-d6 at 60°C.

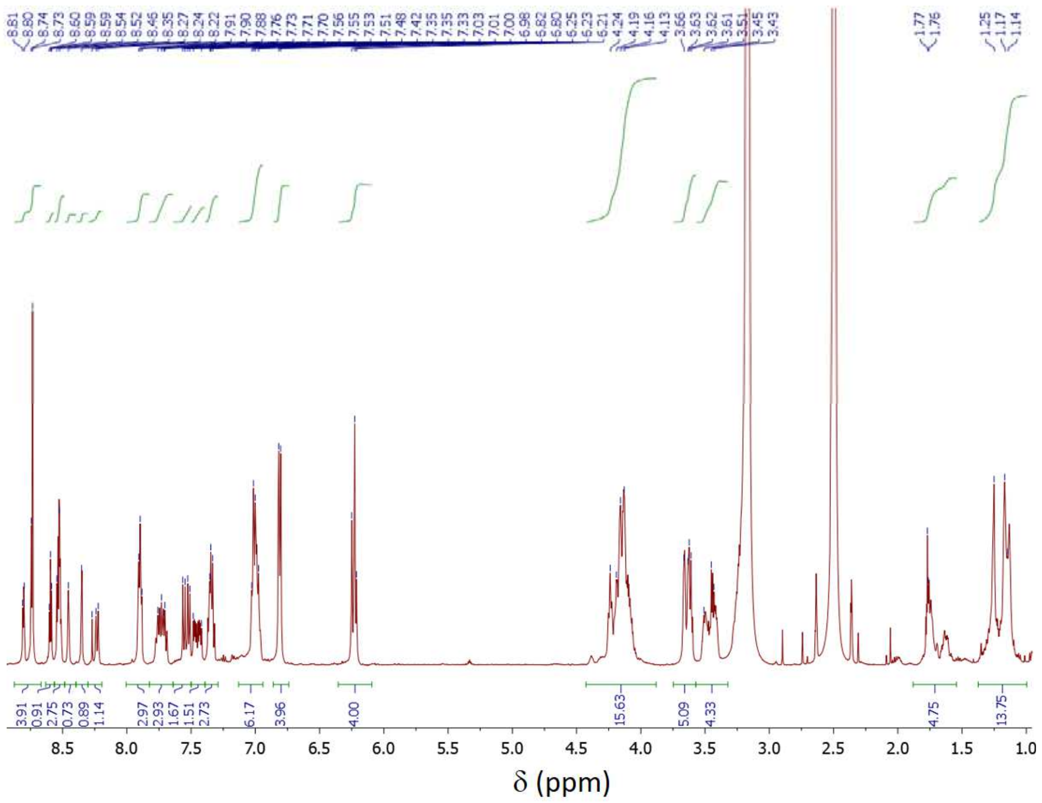

Figure S22 ^{1}H NMR spectrum of compound **$Zn_2C_{11}DC$** in DMSO-d6.

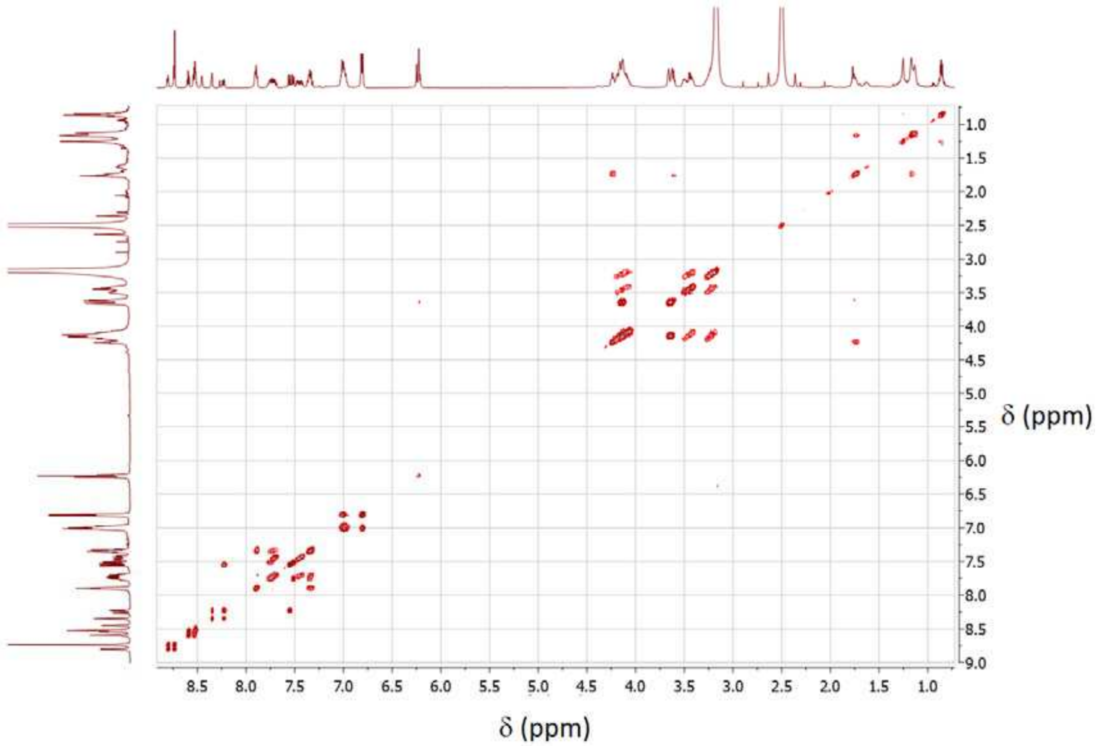

Figure S23 COSY spectrum of compound **$Zn_2C_{11}DC$** in DMSO-d6.

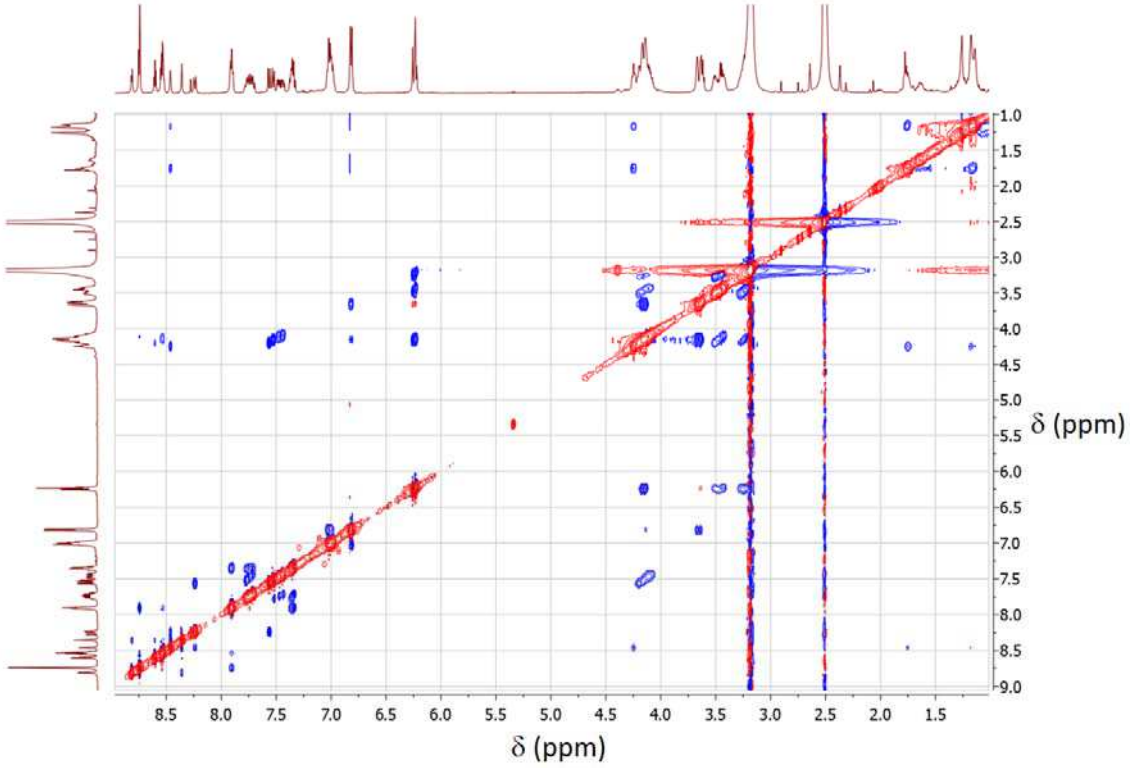

Figure S24 2D-ROESY spectrum of compound **$Zn_2C_{11}DC$** in DMSO-d6.

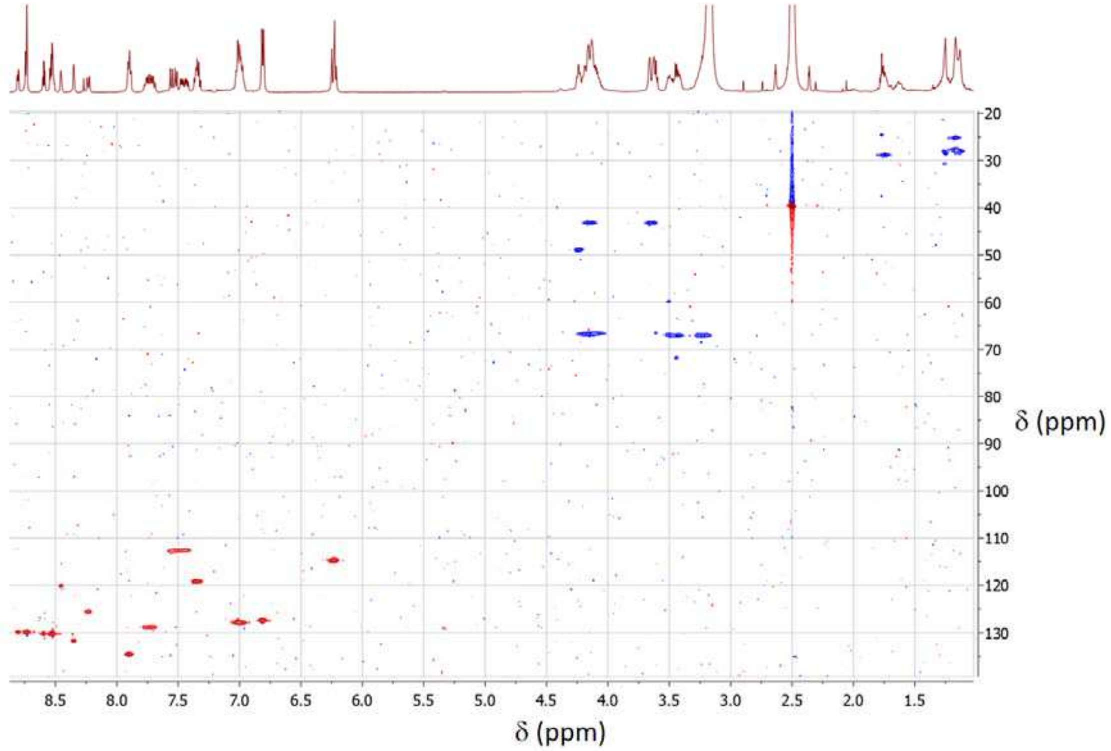

Figure S25 ^{1}H-^{13}C HSQC spectrum of compound **$Zn_2C_{11}DC$** in DMSO-d6 at 60°C.

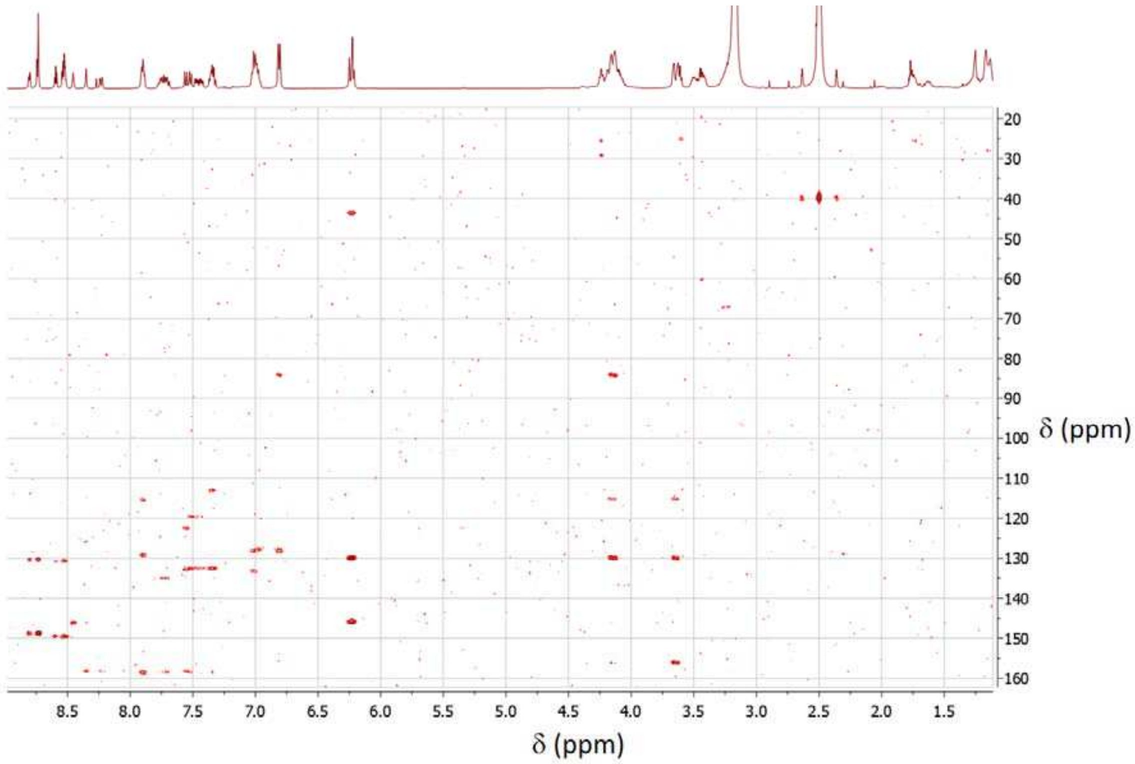

Figure S26 ^{1}H-^{13}C HMBC spectrum of compound **$Zn_2C_{11}DC$** in DMSO-d6 at 60°C.

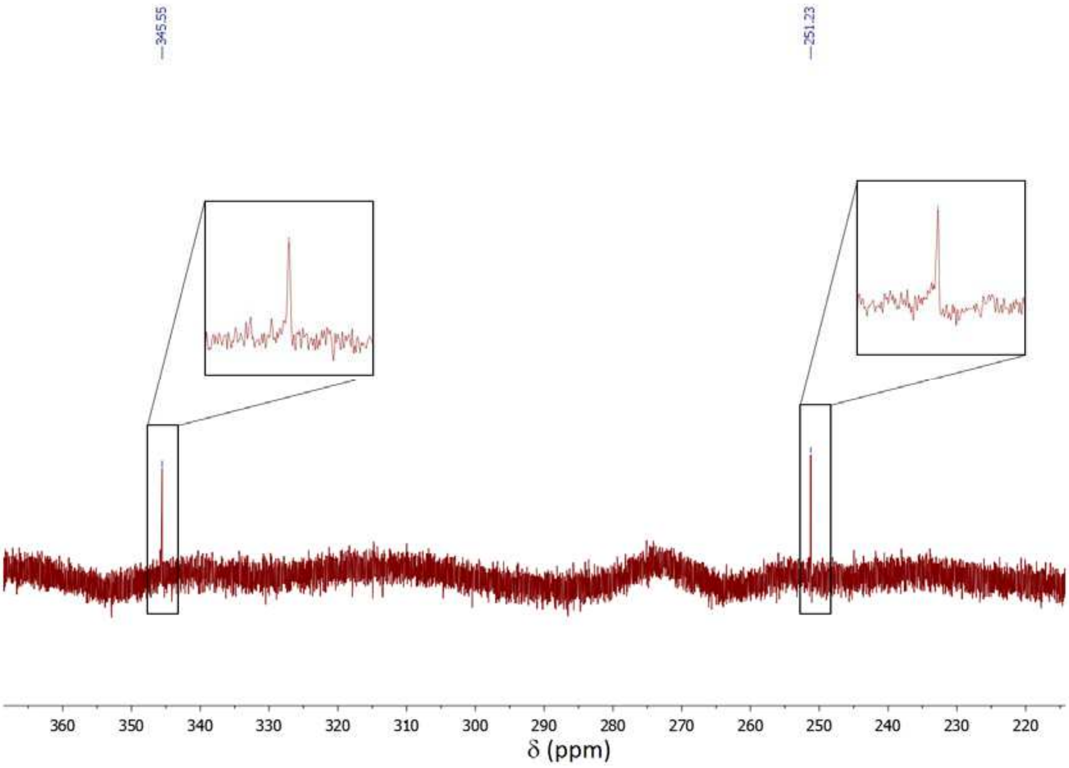

Figure S27 ^{15}N NMR spectrum of compound **$Zn_2C_{11}DC$** in DMSO-d6 at 60°C.

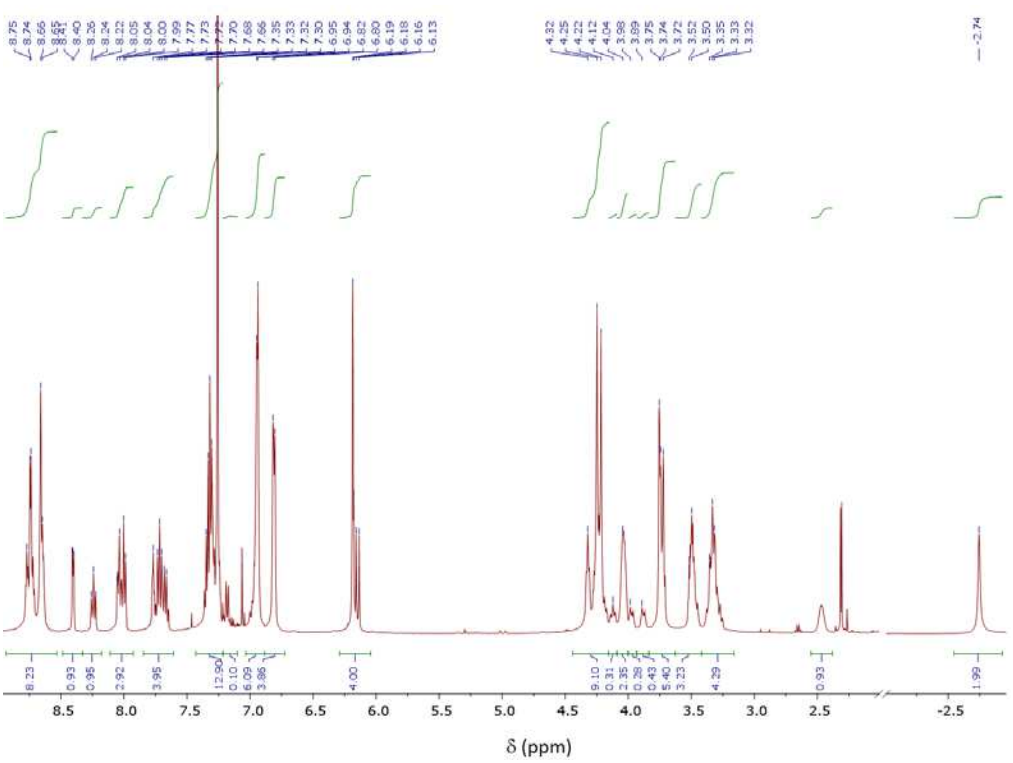

Figure S28 ^{1}H NMR spectrum of compound **H_4C_3DC** in $CDCl_3$.

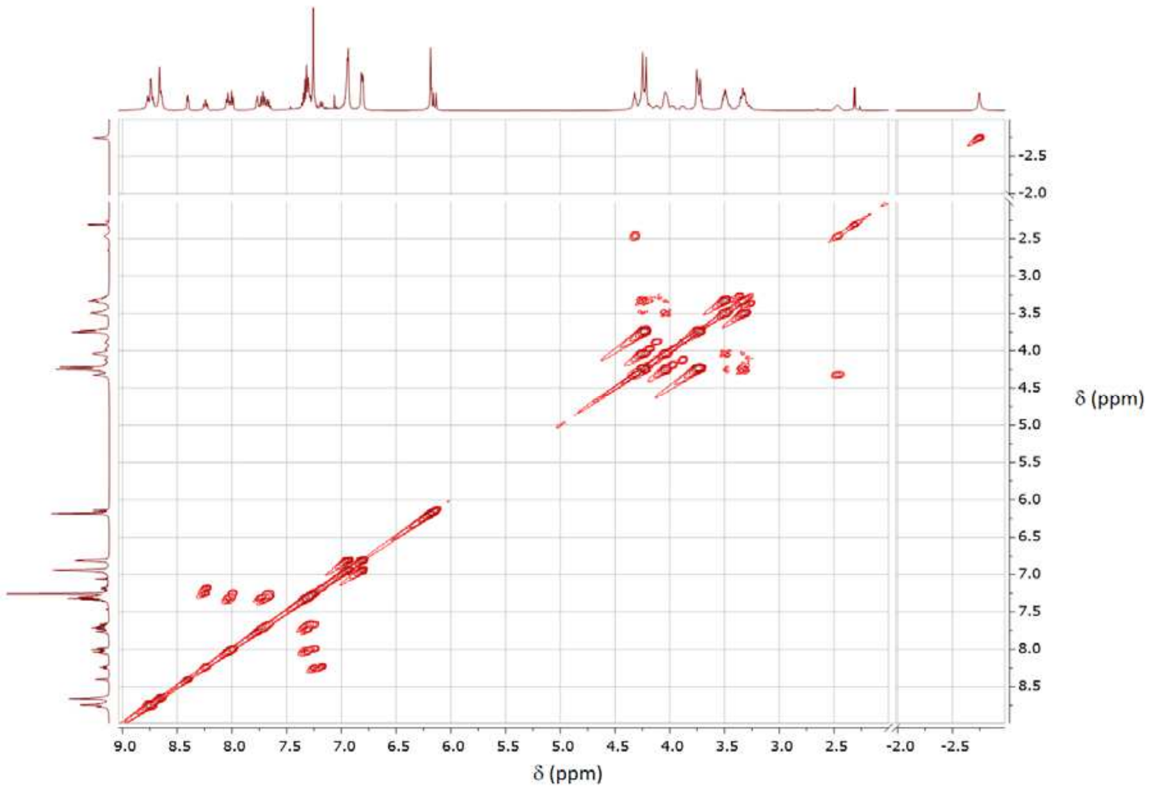

Figure S29 COSY spectrum of compound **H_4C_3DC** in $CDCl_3$.

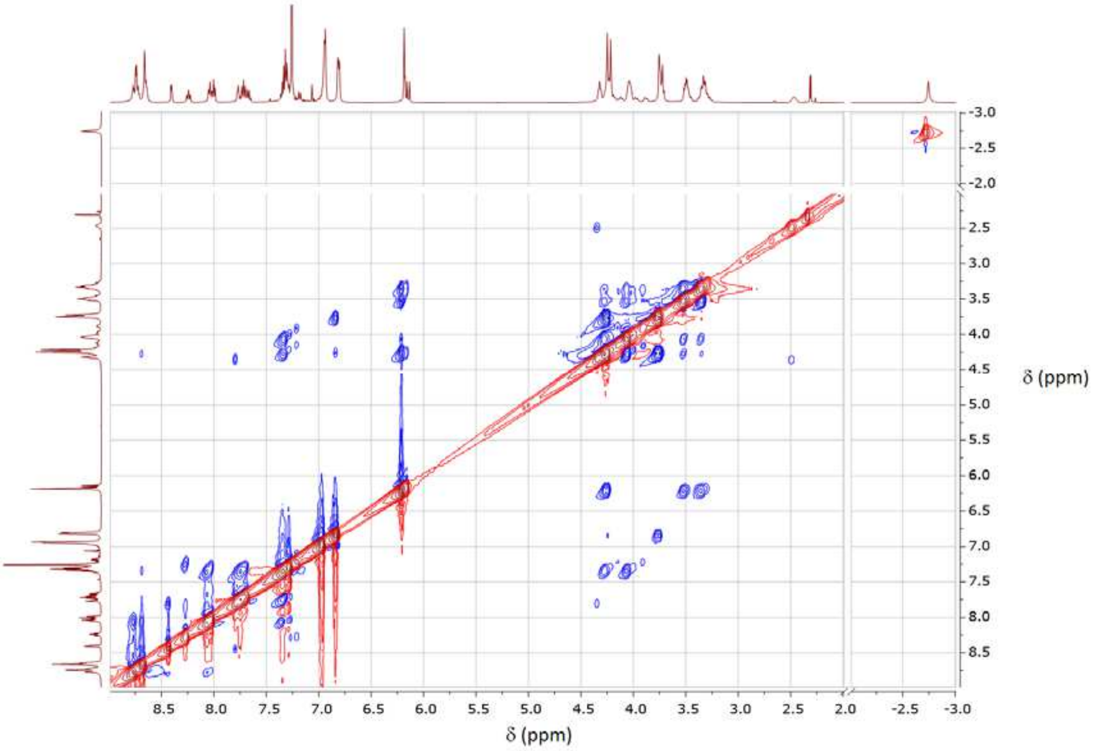

Figure S30 2D-ROESY spectrum of compound $\mathbf{H_4C_3DC}$ in $CDCl_3$.

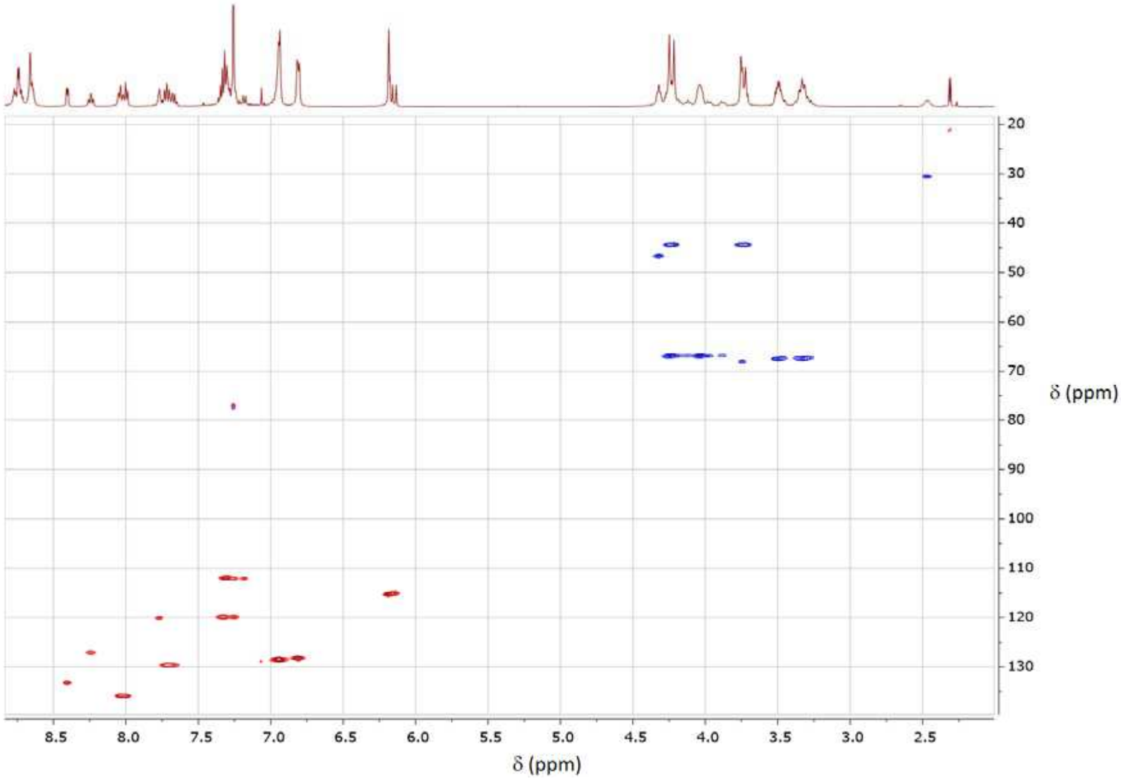

Figure S31 ^{1}H-^{13}C HSQC spectrum of compound $\mathbf{H_4C_3DC}$ in $CDCl_3$.

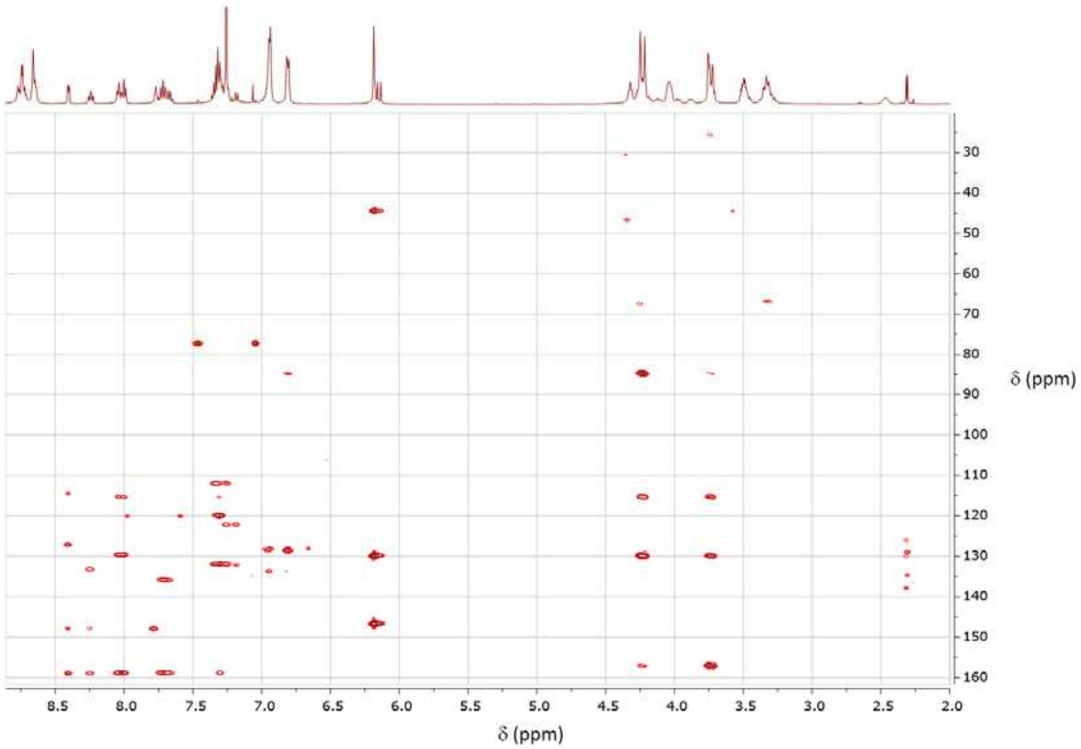

Figure S32 ^{1}H-^{13}C HMBC spectrum of compound **H_4C_3DC** in $CDCl_3$.

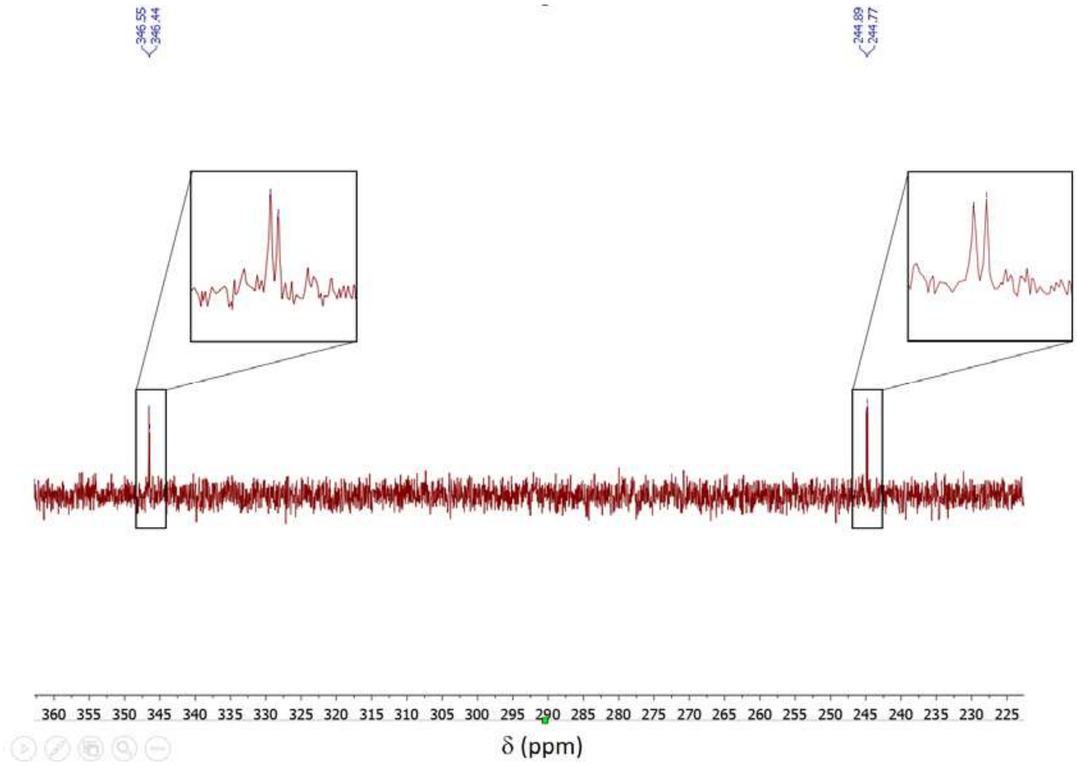

Figure S33 ^{15}N NMR spectrum of compound **H_4C_3DC** in $CDCl_3$.

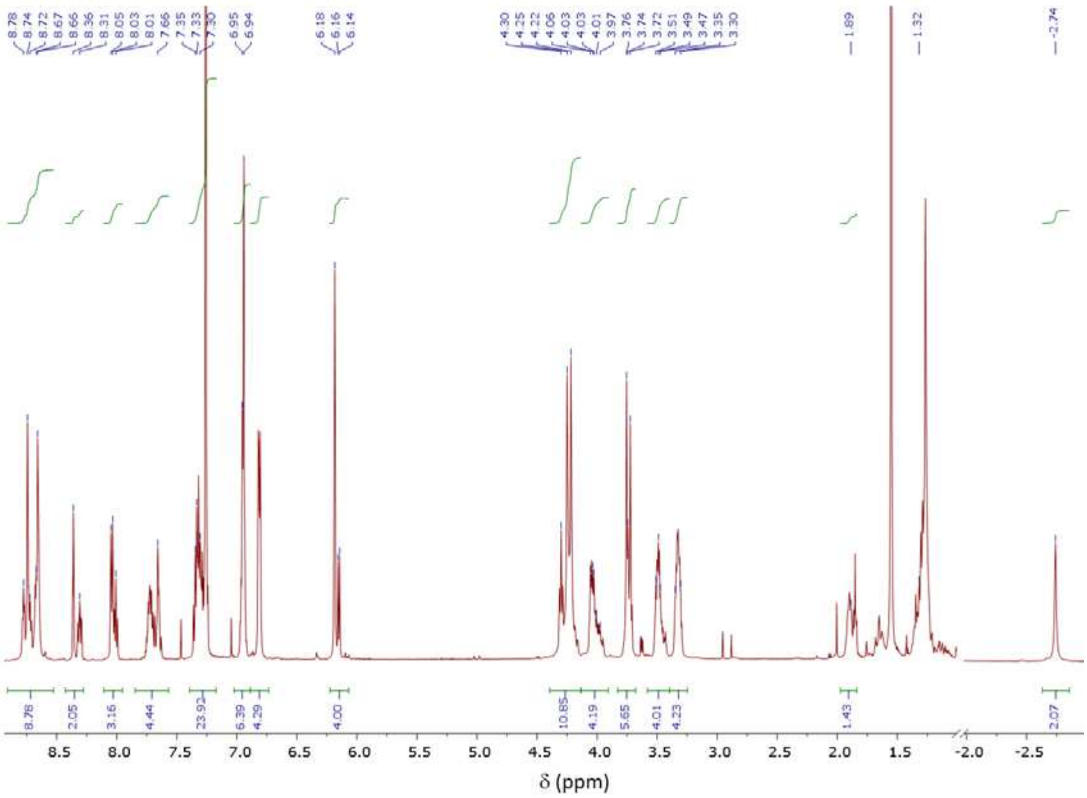

Figure S34 ^{1}H NMR spectrum of compound **H_4C_5DC** in $CDCl_3$.

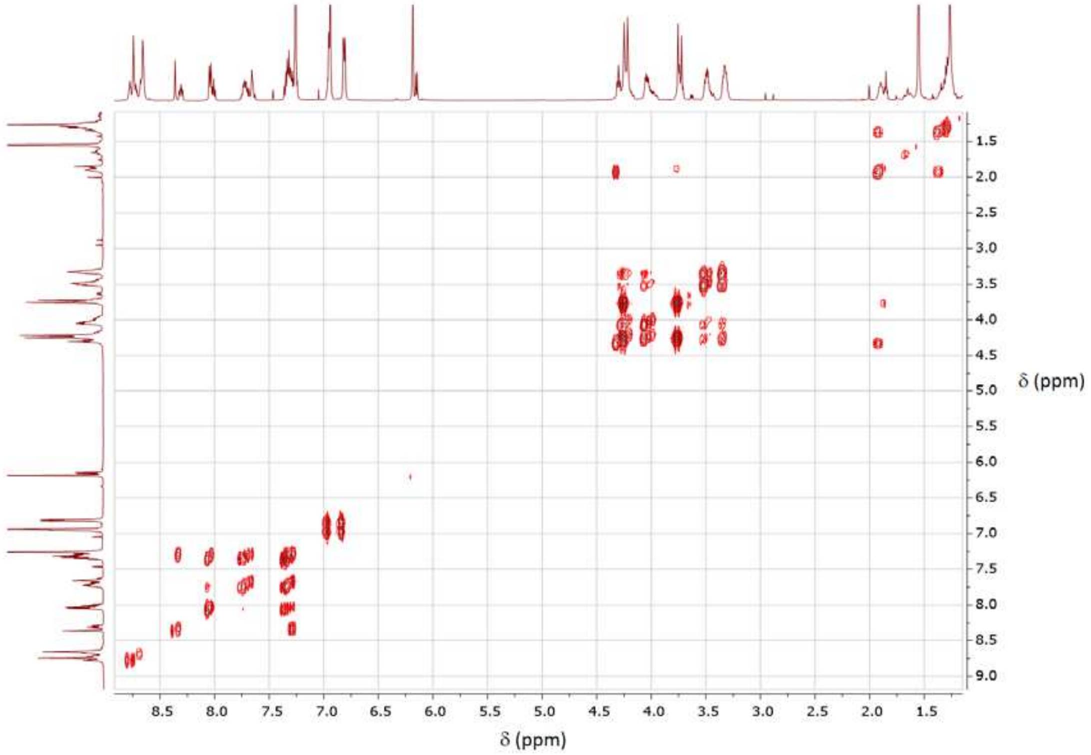

Figure S35 COSY spectrum of compound **H_4C_5DC** in $CDCl_3$.

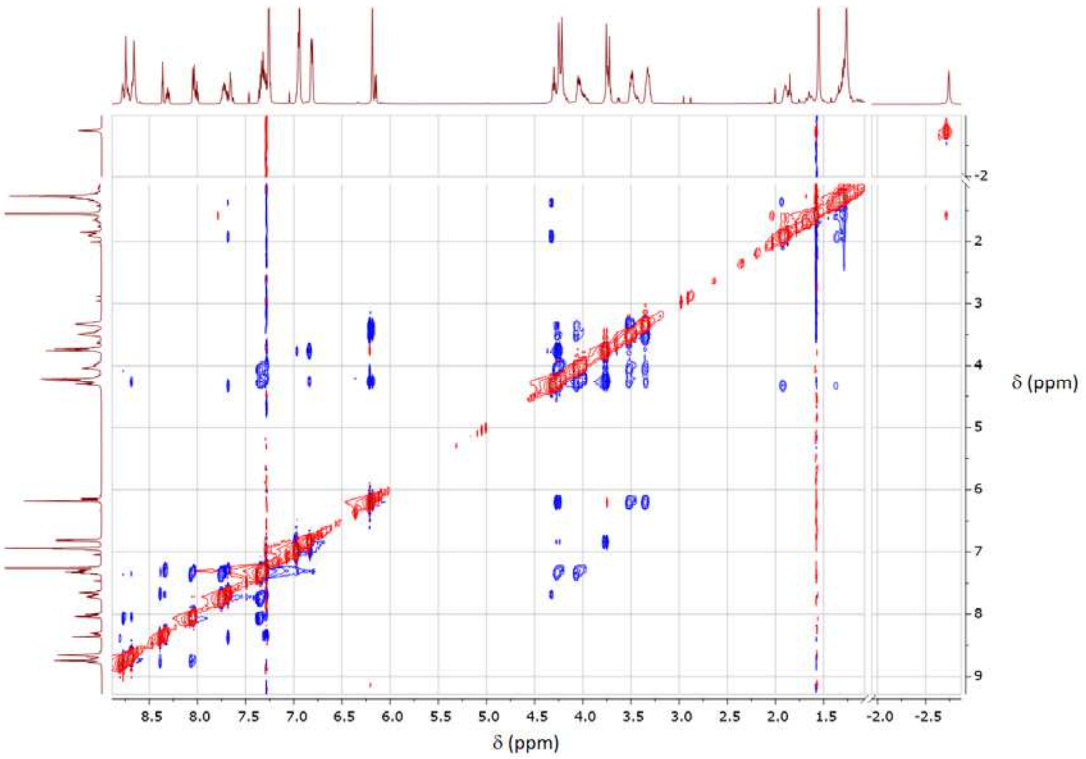

Figure S36 2D-ROESY spectrum of compound **H_4C_5DC** in $CDCl_3$.

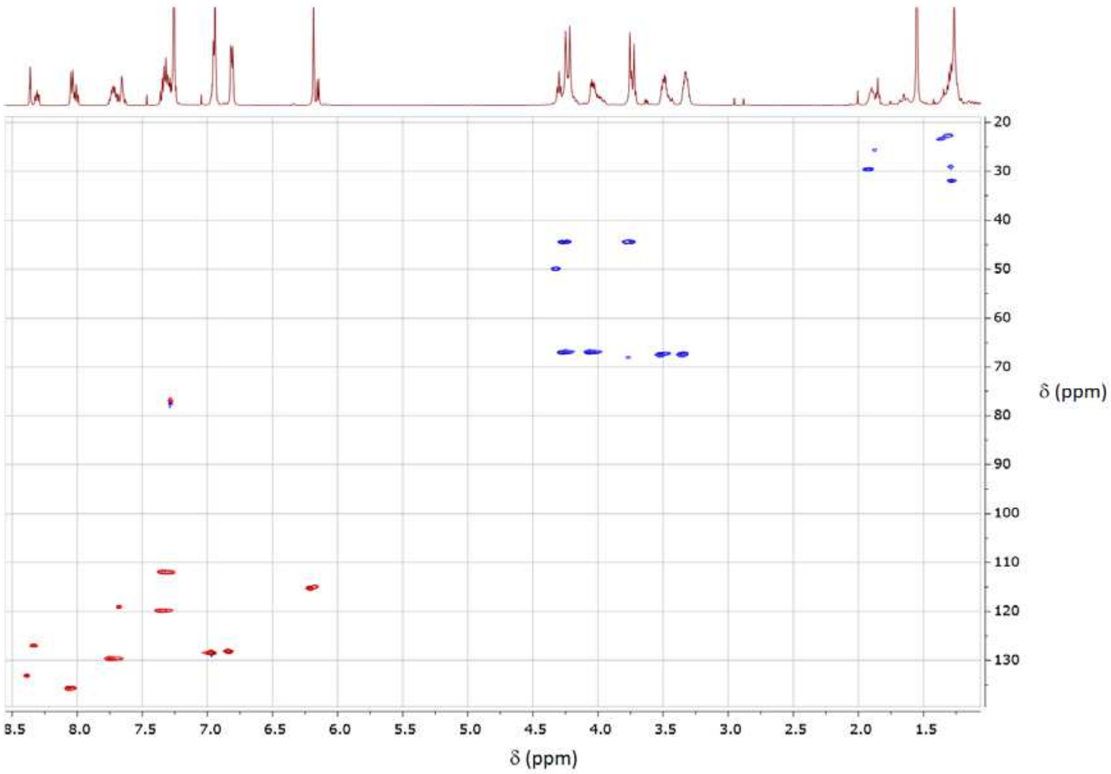

Figure S37 ^1H-^{13}C HSQC spectrum of compound **H_4C_5DC** in $CDCl_3$.

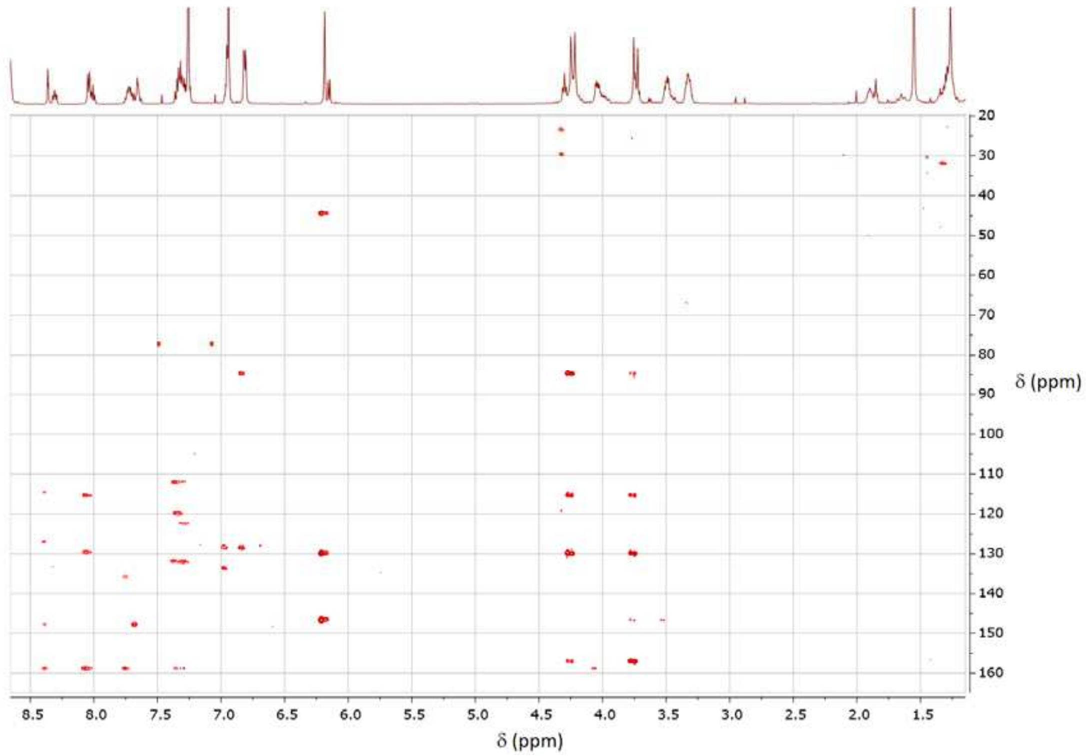

Figure S38 ^{1}H-^{13}C HMBC spectrum of compound **H_4C_5DC** in $CDCl_3$.

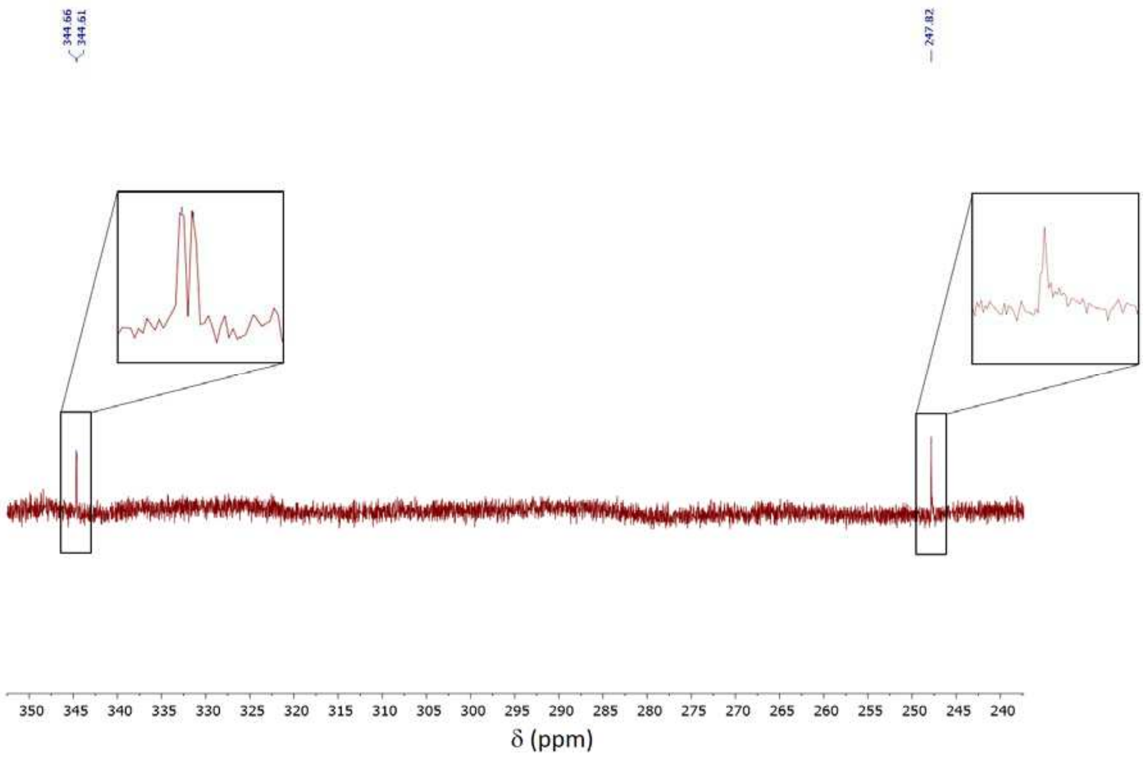

Figure S39 ^{15}N NMR spectrum of compound **H_4C_5DC** in $CDCl_3$.

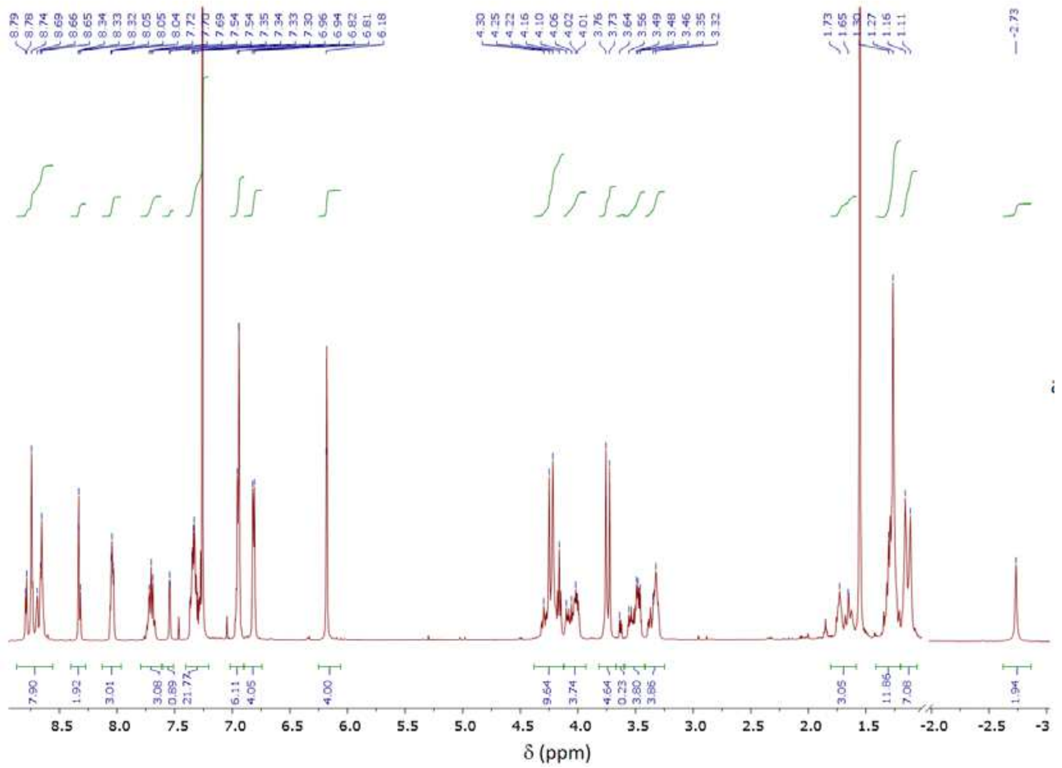

Figure S40 ^{1}H NMR spectrum of compound **$H_4C_{11}DC$** in $CDCl_3$.

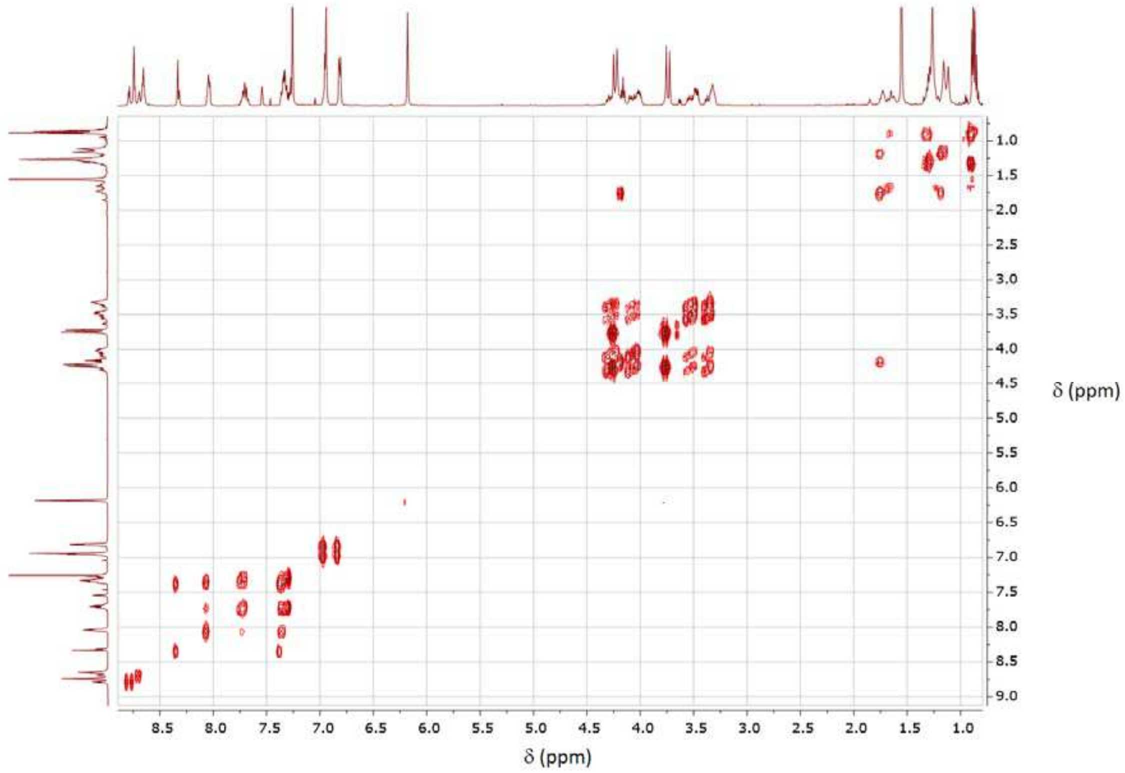

Figure S41 COSY spectrum of compound **$H_4C_{11}DC$** in $CDCl_3$.

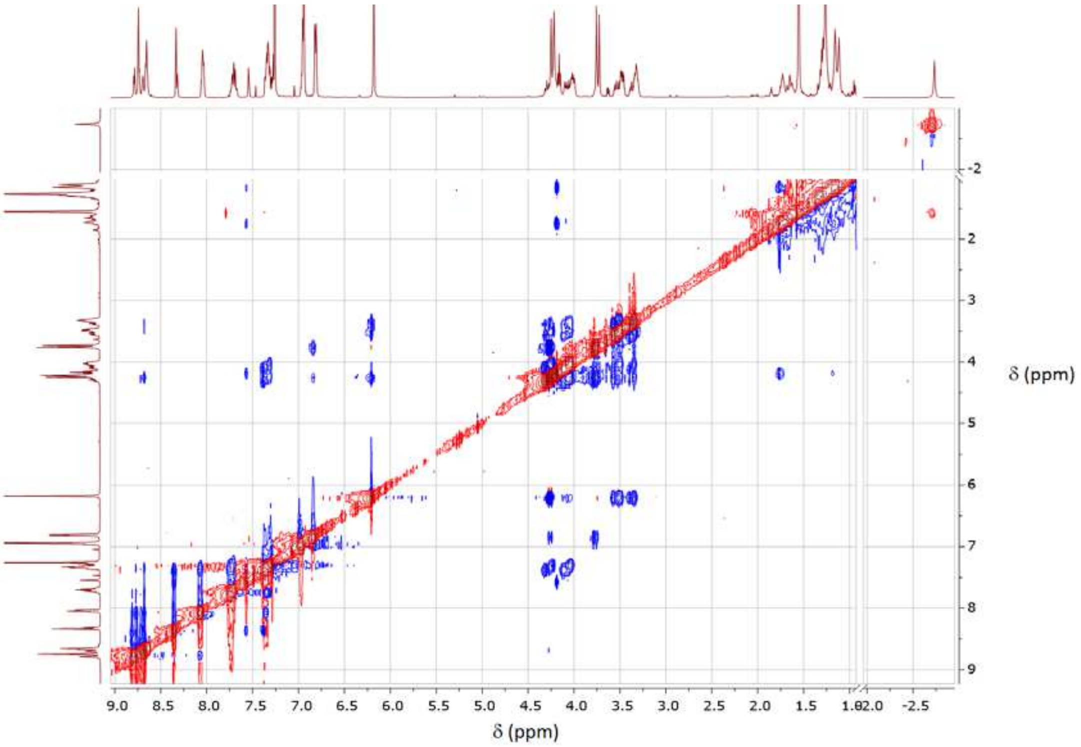

Figure S42 2D-ROESY spectrum of compound $\mathbf{H_4C_{11}DC}$ in $CDCl_3$.

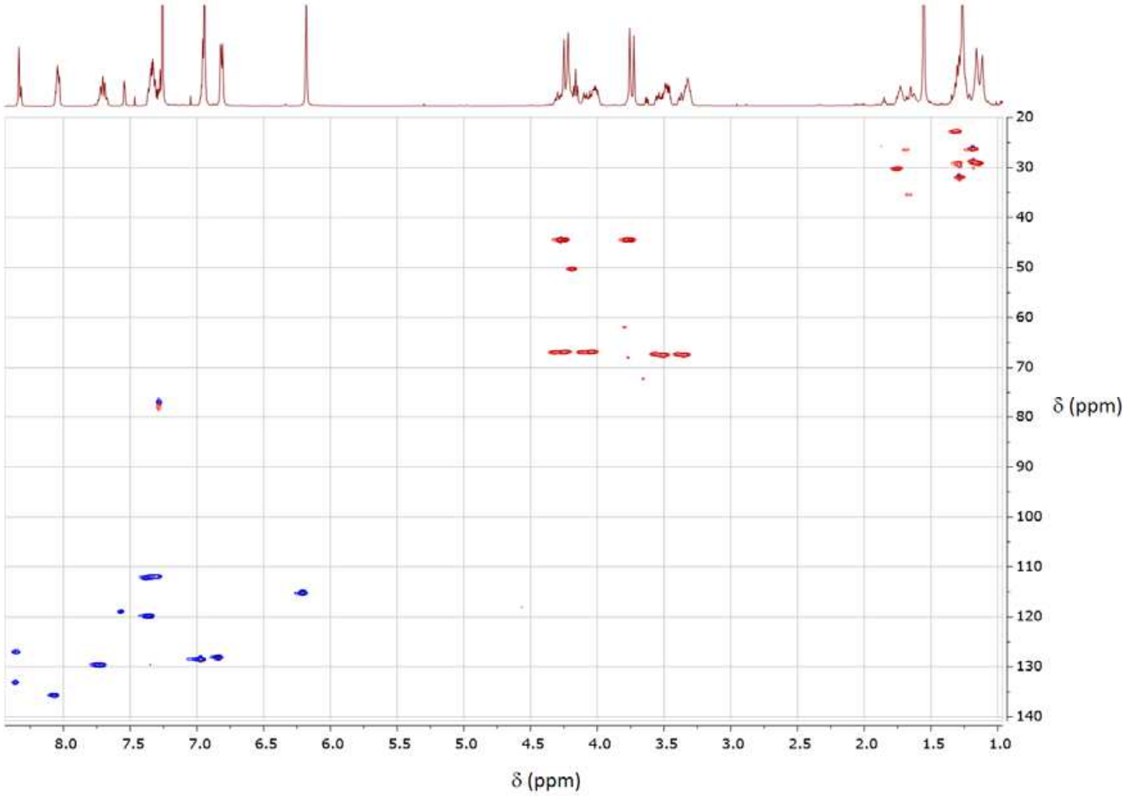

Figure S43 ^{1}H-^{13}C HSQC spectrum of compound $\mathbf{H_4C_{11}DC}$ in $CDCl_3$.

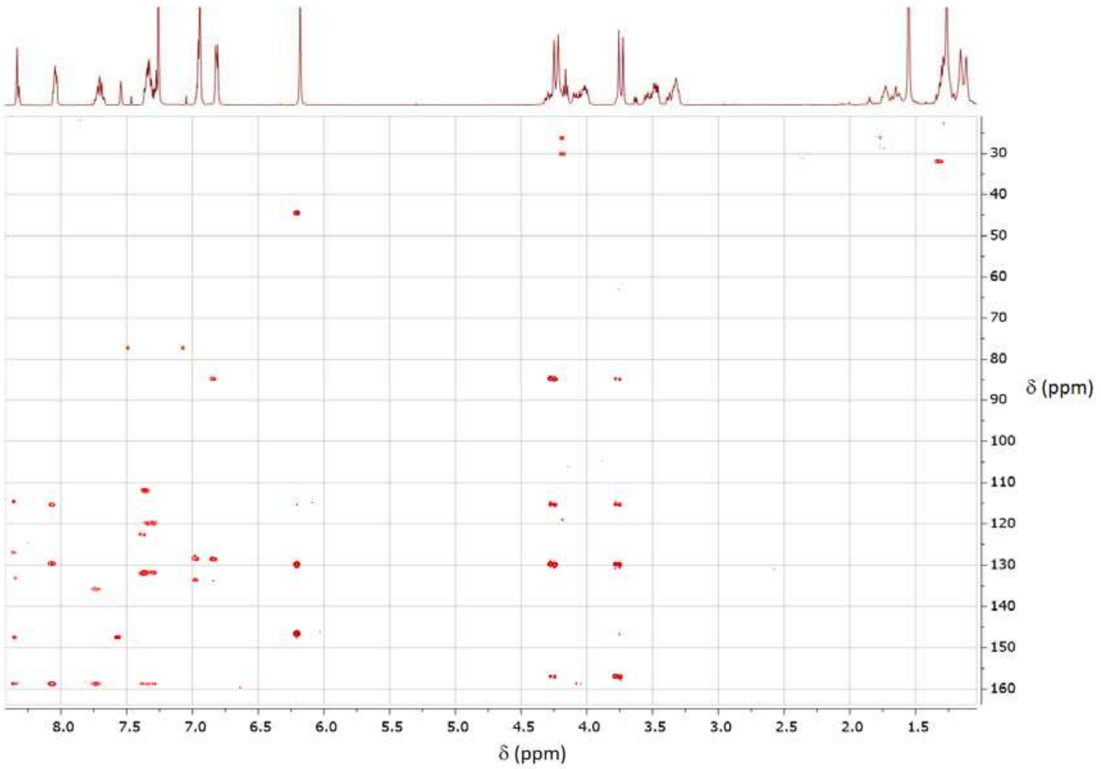

Figure S44 ^1H-^{13}C HMBC spectrum of compound **$H_4C_{11}DC$** in $CDCl_3$.

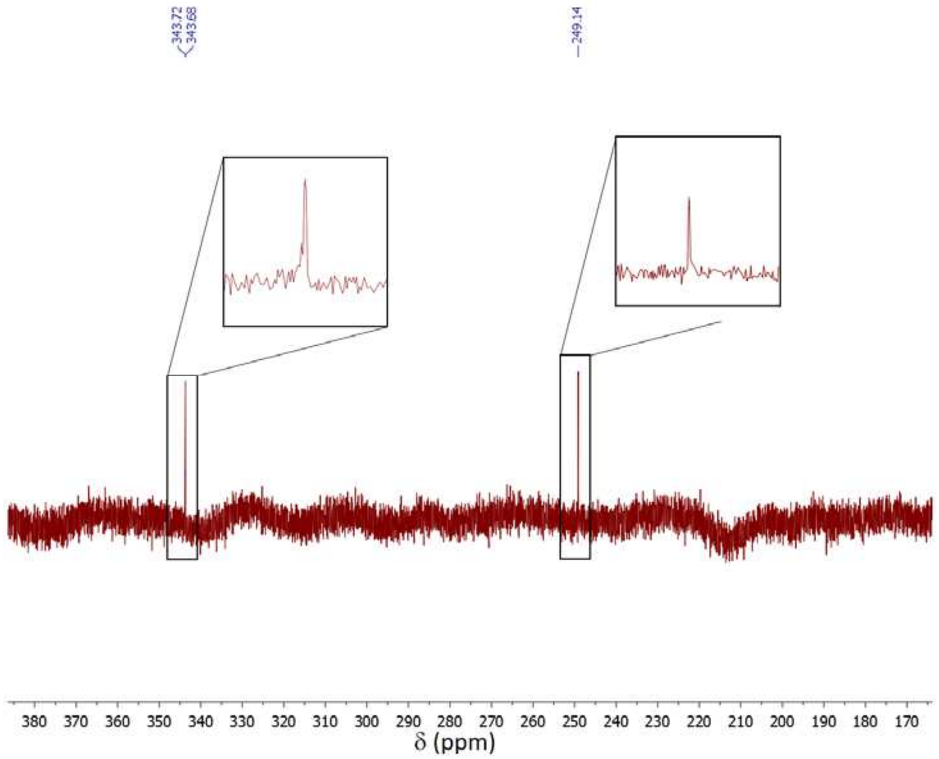

Figure S45 ^{15}N NMR spectrum of compound **$H_4C_{11}DC$** in $CDCl_3$.

Titration methods

2.1 UV-Vis titrations

All titrations were carried out in two-, tree- or fourfold. The 1:1 solvent mixtures were prepared by weighing the desired amounts of the two solvents in a closed Erlenmeyer flask. After thorough mixing of the solvents, the density of the solvent mixture was determined from the weight increase of a 50 mL volumetric flask.

A host stock solution was prepared by quantitatively weighing an amount of the host of between 1 and 2 mg, which was subsequently dissolved in 10 mL solvent or solvent mixture (HostStock). The HostStock was diluted to 1.5 µM (10 mL). All ligand solutions (10 mL) contained 1.5 µM host from diluting HostStock and 5.00^{-5} M (L1), 0.0001 M (L2), 0.0005 M (L3), 0.001 M (L4), 0.1 M (L5) and 1 M (L6) DABCO solution. Solution L6 was prepared by directly weighing the required amount of DABCO in 10 mL of solvent. Solution L5 was prepared from a 0.5 M DABCO stock solution. Solutions L4 and L3 were prepared by diluting solution L5. Solutions L2 and L1 were prepared by diluting solution L3. The volumes of solvents or solvent mixtures required for the host and host-ligand solutions were weighed on an analytical balance and the addition of the ligand solutions to the host solution was carried out with Gilson pipets (up to a volume of 1000 µL).

A UV-Vis spectrum was recorded after every addition of DABCO. The amounts of DABCO ligand solutions titrated to the host are given in Table S1.

Table S1: Table of titrated amounts of DABCO to a solution of ***Zn_2C_xDC*** *(1.5 µM)*

[DABCO] (M)	Equivalents	Volume to add (µL)	Total volume (µL)
**5.00^{-05}**	0	0,00	1500
	0,125	5,65	1505,65
	0,25	5,69	1511,34
**0.0001**	0,5	5,71	1517,05
	0,75	5,75	1522,80
	1	5,80	1528,60
	1,25	5,84	1534,44
	1,5	5,89	1540,32
	1,75	5,93	1546,26
	2	5,98	1552,23
	2,25	6,02	1558,26
	2,5	6,07	1564,33

	2,75	6,12	1570,45
	3	6,17	1576,62
	3,5	12,48	1589,09
	4	12,68	1601,77
**0.0005**	5	4,88	1606,65
	6	4,91	1611,56
	7	4,94	1616,50
	8	4,97	1621,47
	9	5,00	1626,47
	10	5,03	1631,50
	11	5,06	1636,56
	12	5,09	1641,65
	13	5,12	1646,78
	14	5,16	1651,93
	15	5,19	1657,12
**0.001**	17,5	6,38	1663,50
	20	6,43	1669,94
	22,5	6,48	1676,42
	25	6,53	1682,95
	37,5	33,40	1716,35
	50	34,80	1751,15
	75	74,00	1825,15
	100	80,50	1905,65
	200	409,00	2314,65
**0.1**	500	10,49	2325,14
	1000	17,70	2342,84
	2000	36,20	2379,04
	3000	37,40	2416,44
	4000	38,60	2455,04
	5000	39,80	2494,84
**1**	10000	19,00	2513,84
	20000	38,90	2552,74
	50000	124,20	2676,94
	100000	236,20	2913,14
	185000	514,00	3427,14
	218000	250,00	3677,14

The change in absorbance intensity of the Soret band was plotted against the number of equivalents of DABCO added, which gave titration curves that were fitted with the program Bindfit at http://supramolecular.org/ using a standard 1:2 binding model. This model involves two binding constants: $K_{1:1}$ for the formation of the 1:1

Zn_2C_xDC:DABCO sandwich complex and $K_{1:2}$ for the formation of the 1:2 **Zn_2C_xDC**:DABCO open complex. $K_{1:1}$ and $K_{1:2}$ are defined by equations (1) and (2), in which [H] is the concentration of free host, and [HL] and [HL_2] are the concentrations of host-ligand and host-ligand$_2$ complex, respectively.

$$K_{1:1} = \frac{[HL]}{[H][L]} \qquad (1)$$

$$K_{1:2} = \frac{[HL_2]}{[HL][L]} \qquad (2)$$

Equations (1) and (2) can be expressed in terms of absorption of the different Soret bands present in the sample and this yields the binding isotherm to which the titration curves can be fitted as equation (3), in which ΔA is the difference between the total absorption (A) and the initial absorption (A_0), $[H]_0$ is the total concentration of host, and $\Delta\varepsilon_{HL}$ and $\Delta\varepsilon_{HL_2}$ are the molar absorption coefficients in M^{-1}, which are defined from the molar absorption coefficients (ε) of the four individual species that are present during the titration (equations (4) and (5)).

$$\Delta A = A - A_0 = \frac{\Delta\varepsilon_{HL}(K_{11}[H]_0[L]) + \Delta\varepsilon_{HL_2}(K_{11}K_{12}[H]_0[L]^2)}{K_{11}K_{12}[L]^2 + K_{11}[L] + 1} \qquad (3)$$

$$\Delta\varepsilon_{HL} = \varepsilon_{HL} - \varepsilon_H - \varepsilon_L \qquad (4)$$

$$\Delta\varepsilon_{HL_2} = \varepsilon_{HL_2} - \varepsilon_H - \varepsilon_L \qquad (5)$$

For each of the Soret bands equation (3) is calculated, thereby iteratively varying both binding constants simultaneously to yield the smallest error between the fit and the experimentally acquired data per wavelength. Each fitted wavelength has its own set of molar absorption coefficients as these are iteratively varied during the fitting procedure.

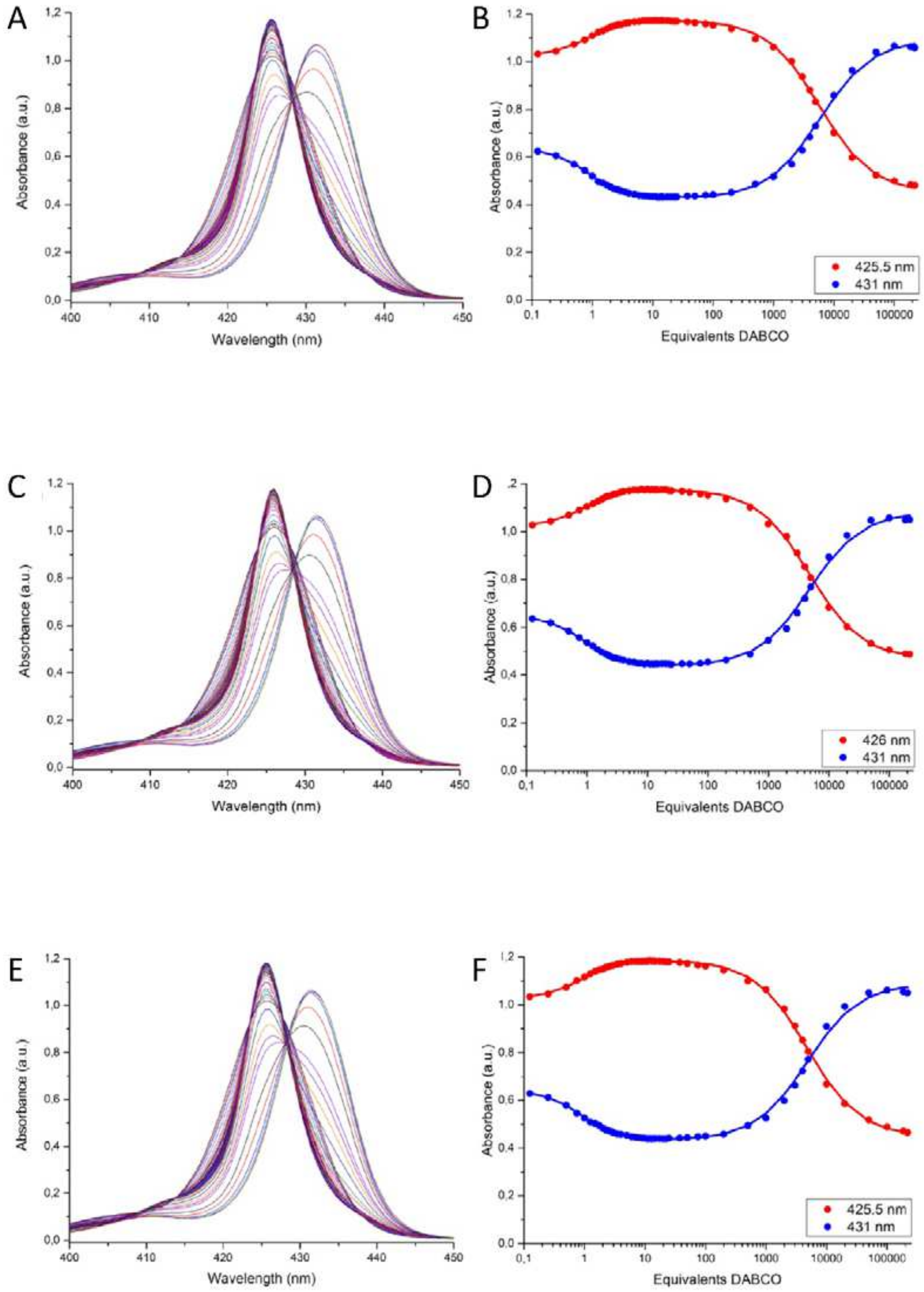

Figure S46 Triplo of UV-vis titrations of **Zn_2C_3DC** with **dabco** in $CHCl_3/CH_3CN$ 1:1 (v/v); A-C-E: UV-vis spectra during the titration; B-D-F: corresponding titration curves.

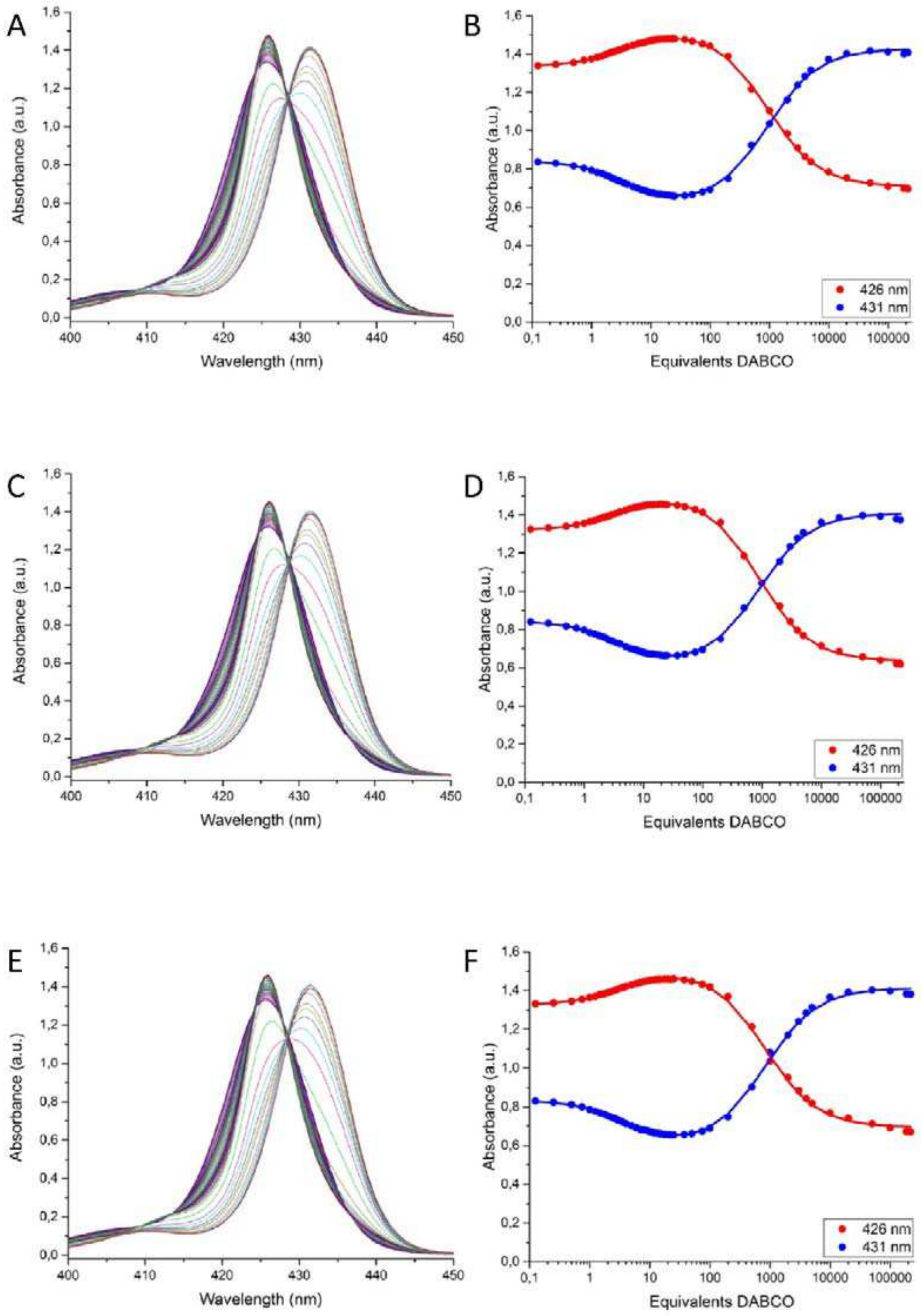

Figure S47 Triplo of UV-vis titrations of **Zn_2C_5DC** with **dabco** in $CHCl_3/CH_3CN$ 1:1 (v/v); A-C-E: UV-vis spectra during the titration; B-D-F: corresponding titration curves.

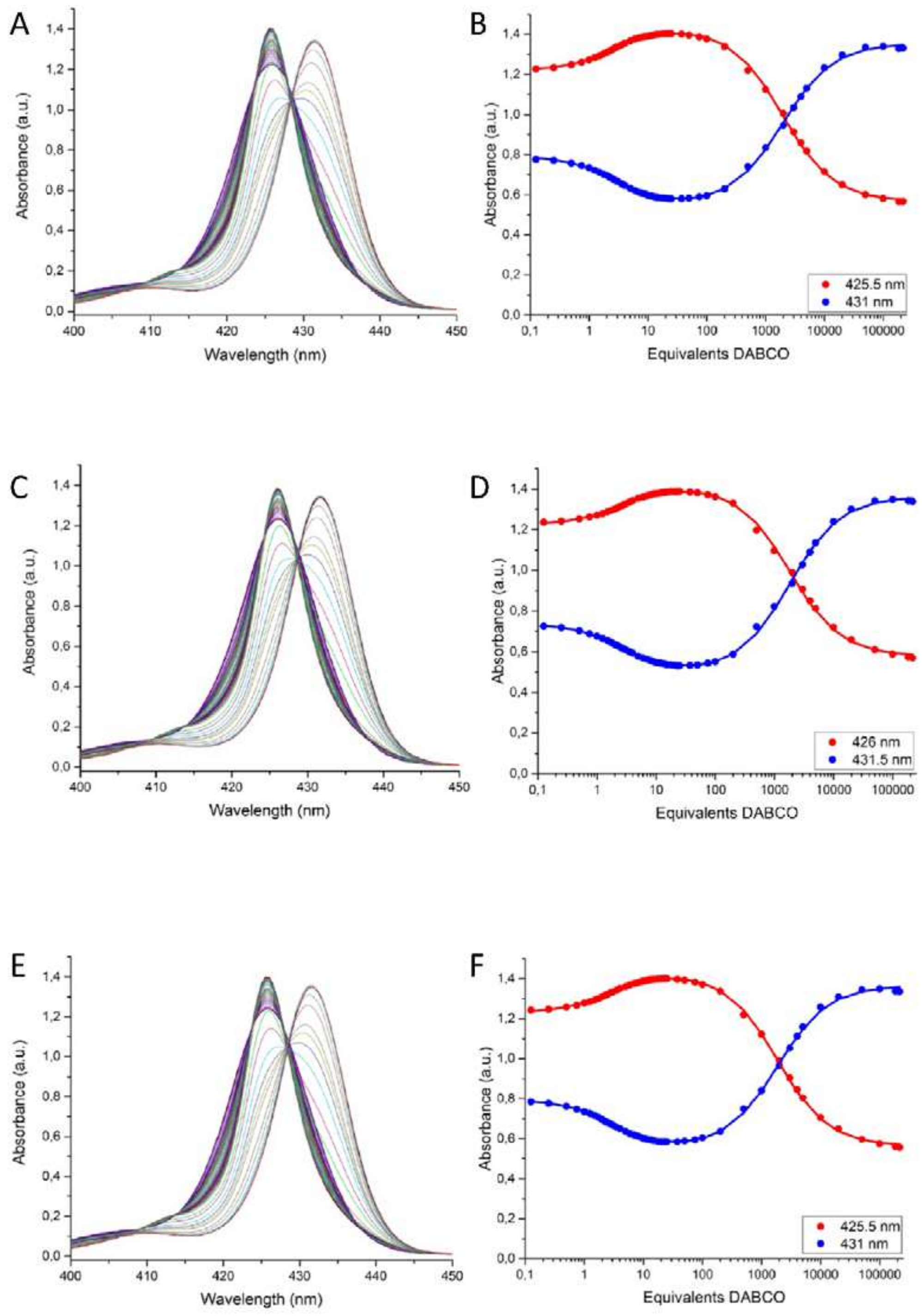

Figure S48 Triplo of UV-vis titrations of **Zn_2C_5DC** with **dabco** in $CHCl_3/CH_3CN$ 1:1 (v/v); A-C-E: UV-vis spectra during the titration; B-D-F: corresponding titration curves.

2.2 ^{1}H-NMR titrations

The 1:1 solvent mixture used for the NMR experiments was prepared by the addition of equal volumes of $CDCl_3$ and MeCN-d_3 with Gilson pipets (up to a volume of 1000 µL) in a test tube. After thorough mixing of the solvents, the desired volume was added to the weighed host (1 – 2 mg) to obtain a host solution with a concentration of about 1 mM. Small amounts of ligand solution (up to 134 µL) were added with a Gilson pipet to the host solution until up to 8 equivalents of ligand were present. After each addition of ligand, a ^{1}H-NMR spectrum was recorded after an equilibration time of 5 minutes at the desired temperature.
